# Metabolomics and quantitative analysis to determine differences in the geographical origins and species of Chinese dragon’s blood

**DOI:** 10.3389/fpls.2024.1427731

**Published:** 2024-09-18

**Authors:** Xiuting Sun, Qing Huang, Mingsong Wu, Liu He, Xiangsheng Zhao, Xinquan Yang

**Affiliations:** ^1^ Hainan Provincial Key Laboratory of Resources Conservation and Development of Southern Medicine, Hainan Branch of the Institute of Medicinal Plant Development, Chinese Academy of Medical Sciences and Peking Union Medical College, Haikou, China; ^2^ Institute of Medicinal Plant Development, Chinese Academy of Medical Sciences and Peking Union Medical College, Beijing, China; ^3^ College of Life Science, Sichuan University, Chengdu, China

**Keywords:** Chinese dragon’s blood, geographical origins, species, metabolomics, UHPLC-PDA, quality control

## Abstract

**Objective:**

The aim of this study was to comprehensively analyze the differences in Chinese dragon’s blood (CDB), specifically *Dracaena cochinchinensis* and *Dracaena cambodiana*, from different geographical origins.

**Methods:**

Metabolomic analysis of CDB was performed by liquid chromatography-tandem mass spectrometry (LC–MS/MS). A reliable ultrahigh-performance liquid chromatography method with a photodiode array detector (UHPLC-PDA) was developed and applied for the quantitative analysis of 12 phenolic compounds in 51 batches of samples.

**Results:**

A total of 1394 metabolites were detected, of which 467 were identified as differentially accumulated metabolites. Multivariate analysis revealed that both origin and species had an effect on the composition of CDB, with greater variation between species. 19 phenolic compounds were selected as quality markers to distinguish *D. cochinchinensis* (Hdsp) from *D. cambodiana* (Hdca), and oppositin and spinoflavanone a were identified as quality markers to discriminate *D. cochinchinensis* samples from Hainan (Hdsp) and Guangxi Provinces (Gdc). Quantitative analysis indicated that four phenolic compounds, including loureirin D, 4H-1-benzopyran-4-one,2,3-dihydro-3,5,7-trihydroxy-3-[(4-methoxyphenyl)methyl]-,(R)-, loureirin B, and pterostilbene, showed significant differences between Gdc and Hdsp. Additionally, five phenolic compounds, namely resveratrol, loureirin D, pinostilbene, 4H-1-benzopyran-4-one,2,3-dihydro-3,5,7-trihydroxy-3-[(4-methoxyphenyl)methyl]-, (R)-, and loureirin B, exhibited significant differences between Hdsp and Hdca.

**Conclusion:**

There are significant differences in the quality of CDB from different geographical origins and species, which lays the foundation for the in-depth development and utilization of different sources of CDB.

## 1 Introduction

Chinese dragon’s blood (CDB, *Longxuejie* in Chinese) is a rare and valuable traditional Chinese medicine (TCM) with a long history of medical use and is known as a “holy medicine for promoting blood circulation”. Previous phytochemical investigations have revealed that polyphenols, terpenes, steroids and steroidal saponins are major chemical constituents of this herb (Xu et al., 2010; Sun et al., 2017; Pang et al., 2018; Sun et al., 2019; Chen P. Q. et al., 2023). Among these constituents, phenolic constituents are considered the primary pharmacologically active constituents (Lang et al., 2020; Xu et al., 2022). At present, the Chinese Pharmacopoeia does not include CDB, and loureirin B are chosen as marker compounds to assess the quality (>0.4%, *m/m*) in the National Drug Standard [WS3-082(Z-016)-99(Z)] (Zheng et al., 2021). In recent years, the metabolites extracted from the resin have received increased attention for their extensive and promising bioactivities, such as antifungal (Niu et al., 2020), antibacterial (Wang et al., 2017; He et al., 2021), anticerebral ischemia (Krishnaraj et al., 2019; Liu T. et al., 2023), antithrombotic (Sun et al., 2019), antidiabetic (Liu Y. et al., 2021), anti-inflammatory and analgesic (Kuo et al., 2017; Sun et al., 2017; Pang et al., 2018) effects and the ability to promote epidermal repair (Chen et al., 2021; Sun et al., 2017).

The quality of TCMs is closely related to their metabolites (Wang et al., 2015; Lu and Cui, 2019). During production, these metabolites are susceptible to not only interference and changes caused by genetic factors but also various physiological and environmental factors, which may lead to differences in the pharmacological effects and activities of TCMs (Yang et al., 2020; Chen X. B. et al., 2023; Sha et al., 2023). Therefore, the study of variations in the metabolites of TCM has been a consistent focus. As a multiorigin TCM, CDB is derived from the resin of *Dracaena cochinchinensis* and *Dracaena cambodiana* in China. *D. cochinchinensis* is cultivated mainly in Guangxi and Hainan Provinces of China (Cai and Xu, 1979), whereas *D. cambodiana* is found primarily in Hainan Province, China (Yuan, 1991). To date, research on the influence of the plant species and geographical factors on the chemical components of CDB is relatively limited and has focused mainly on determining the differences in the contents of a few specific classes of metabolites (Qin et al., 2013; Wan, 2017; Chen et al., 2018). The chemical composition of TCMs is complex, and the current TCM quality evaluation model relies mainly on the determination of a few compounds, which makes accurate evaluate of the quality of TCMs difficult. Thus, there is an urgent need to identify additional metabolites of CDB to evaluate the overall chemical features of samples collected from different geographical origins and of different species.

Plant metabolomics, a branch of metabolomics, is a powerful tool designed to study the overall changes in many metabolites in plant samples and then perform deep data mining and bioinformatics to identify differences between samples (Abraham and Kellogg, 2021; Xiao et al., 2022). Liquid chromatography-tandem mass spectrometry (LC–MS/MS) is one of the most frequently used techniques for plant metabolomics analysis of TCMs because of its short analysis time, exact mass identification, and high selectivity and sensitivity (Alseekh et al., 2021; Waris et al., 2022). Plant metabolomics has been widely applied for source identification, authenticity identification, processing method evaluation and other quality control applications of TCM (Liang et al., 2018; Abraham and Kellogg, 2021; Yoon et al., 2022; Liu X. et al., 2023; Rivera-Pérez et al., 2023). This finding offers a valuable opportunity to understand the metabolite differences that influence the medicinal quality of CDB from different geographical origins and species. However, there are no studies on plant metabolomics for CDB. In addition, quantitative analysis based on ultrahigh-performance liquid chromatography-photodiode array detector (UHPLC-PDA) technology has become a widely used method for TCM quality control and quality standard improvement (Lee et al., 2010; Liao et al., 2021).

In this study, a total of 51 samples were collected, including samples of *D. cochinchinensis* from Guangxi Province (Gdc) and Hainan Province (Hdsp), and *D. cambodiana* from Hainan Province (Hdca). A plant metabolomic strategy based on LC–MS/MS was established to identify the differentially abundant metabolites (DAMs) in 21 samples. A UHPLC-PDA method was developed to quantitatively assess the variation of 12 phenolic compounds in 51 samples. This study enhances the understanding of the differences between the geographical origins and species of the CDB. The results will contribute to effective quality control measures and provide a theoretical basis for the rational and effective use of CDB.

## 2 Materials and methods

### 2.1 Samples

Fifty-one batches of wild *D. cochinchinensis* and *D. cambodiana* samples were collected from natural habitats in Hainan and Guangxi Provinces, China (**Supplementary Table S1**). The samples were identified by associate researcher Rongtao Li (Hainan Branch of the Institute of Medicinal Plant Development, Chinese Academy of Medicinal Sciences), and voucher specimens (No. Gdc-24, Hdsp-8, Hdca-2) were deposited in the herbarium of the Hainan Branch Institute of Medicinal Plant Development, Chinese Academy of Medical Sciences, Hainan, China. All the samples were utilized for content determination, and 21 samples were selected from the three groups for plant metabolomics analysis.

### 2.2 Solvents and chemicals

The reference material for CDB (*D. cochinchinensis*, collected from Guangxi Province) was purchased from the National Institutes of Food and Drug Control (Beijing, China). Reference standards of *p*-hydroxybenzyl alcohol (LOT: 6100) were purchased from Shanghai Shidande Standard Technical Service Co., Ltd. (Shanghai, China). Loureirin D (LOT: RFS-L02801907025) and loureirin C (LOT: RFS-L05901907025) were purchased from Chengdu Herbpurify Co., Ltd. (Chengdu, China). 4H-1-benzopyran-4-one,2,3-dihydro-5-hydroxy-3-[(4-hydroxyphenyl) methyl]-7-methoxy (LOT: CHB210112), liquiritigenin (LOT: CHB180609), pinostilbene (LOT: CHB210104) and 4H-1-benzopyran-4-one,2,3-dihydro-3,5,7-trihydroxy-3-[(4-methoxyphenyl) methyl]-,(R)- (LOT: CHB210113) were purchased from Chroma-Biotechnology Co., Ltd. (Chengdu, China). Resveratrol (LOT: 137-12-47), (3R)-5,7-dihydroxy-3-(4-hydroxybenzyl)-2,3-dihydro-4H-chromen-4-one (LOT: CHB210113), loureirin A (LOT: 0817-RB-0015), loureirin B (LOT: 0817-RB-0016) and pterostilbene (LOT: 0817-RB-0036) were purchased from Hainan Lead and Change Technology Co., Ltd. (Haikou, China). The purities of all standards were above 98%. HPLC-grade acetonitrile and acetic acid were purchased from Fisher Co., Ltd. (Emerson, IA, USA). Analytical-grade ethanol was purchased from Beijing Chemical Works (Beijing, China). Pure water was obtained from Wahaha (Hangzhou, China).

### 2.3 Plant metabolomic analysis

#### 2.3.1 Metabolite extraction

A total of 20 mg of sample was weighed into an eppendorf tube, and 1000 μL of extraction solution (methanol:water = 3:1, with an isotopically labeled internal standard mixture) was added. The sample was subsequently homogenized at 35 Hz for 4 min and sonicated for 5 min in an ice-water bath. The homogenization and sonication cycles were conducted in triplicate. The samples were subsequently tincubated for 1 h at -40°C and centrifuged at 12000 rpm for 15 min at 4°C. The supernatant was filtered through a 0.22 μm microporous membrane and transferred to a fresh glass vial for analysis. The quality control (QC) sample was prepared by mixing equal aliquots of the supernatants from all of the samples to evaluate the reproducibility and stability of the LC-MS/MS method.

#### 2.3.2 LC–MS/MS analysis

Sample analyses were performed on a UHPLC system (Vanquish, Thermo Fisher Scientific) with a UPLC HSS T3 column (2.1 mm × 100 mm, 1.8 μm) coupled to an Orbitrap Exploris 120 mass spectrometer (Orbitrap MS, Thermo), and the column temperature was 40°C. The mobile phase consisted of 5 mmol/L ammonium acetate and 5 mmol/L acetic acid in water (A) and acetonitrile (B) with flow 0.35 ml/min. The following gradient program was used: 0-0.7 min, 1% B; 0.7-9.5 min, 1%-99% B; 9.5-11.8 min, 99% B; 11.8-12 min, 99%-1% B; 12-14.6 min, 1% B; 14.6-14.8 min, 1% B; 14.8-15 min, 1% B. The autosampler temperature was 4°C, and the injection volume was 2 μL. An Orbitrap Exploris 120 mass spectrometer was used for its ability to acquire MS/MS spectra in information-dependent acquisition (IDA) mode via acquisition software (Xcalibur, Thermo). In this mode, the acquisition software continuously evaluates the full-scan MS spectrum. The electrospray ionization (ESI) source conditions were set as follows: sheath gas flow rate, 50 Arb; aux gas flow rate, 15 Arb; capillary temperature, 320°C; full MS resolution, 60000; MS/MS resolution, 15000; collision energy, 10/30/60 NCE; and spray voltages, 3.8 kV (positive) and -3.4 kV (negative).

### 2.4 Data processing and metabolite characterization

The raw data were converted to the mzXML format via ProteoWizard and processed with an in-house program, which was developed using R and based on XCMS, for peak detection, extraction, alignment, and integration. The criteria for the structural identification of metabolites are categorized into three levels. Level 1 is achieved by comparing information such as MS, MS/MS, and retention times of standards. Level 2 is achieved by comparing information such as MS and MS/MS from public databases, which include HMDB (https://hmdb.ca/), PubChem (https://pubchem.ncbi.nlm.nih.gov/), and KEGG (https://www.kegg.jp/). Finally, level 3 is achieved by comparing information such as MS, MS/MS, and retention time using a matching algorithm based on MetDNA2 (BiotreeDB) (Zhou et al., 2022). The cutoff for annotation was set at 0.3. Furthermore, principal component analysis (PCA) and orthogonal partial least squares discriminant analysis (OPLS-DA) were performed with SIMCA 16.0.2 software (Sartorius Stedim Data Analytics AB, Umea, Sweden). To evaluate the OPLS-DA model, 7-fold cross-validation was performed to calculate the R^2^ (model fitness) and Q^2^ (predictive ability) values, and a permutation test (200 times) was subsequently conducted.

### 2.5 Potential biomarker identification and metabolic pathway analysis

In this study, the metabolites with variable importance in the projection (VIP) > 1 and a P value < 0.05 (Student’s t test) were screened as potential differentially abundant metabolites. After the metabolites were identified, the metabolic enrichment pathways of potential DAMs were analyzed via MetaboAnalyst (http://metpa.metabolomics.ca) and the Kyoto Encyclopedia of Genes and Genomes (KEGG) pathway database (http://www.genome.jp/kegg/) with a map of the same plant family, *Asparagus officinalis*. Through comprehensive analysis of the pathways in which the DAMs were located, the most important pathways that correlated with the metabolite differences were screened.

### 2.6 UHPLC-PDA analysis

#### 2.6.1 Preparation of stock and mixed standards

Each standard was accurately weighed and dissolved in 70% aqueous ethanol (*v/v*) to obtain a stock solution. An initial stock solution was prepared as a mixture of the above stock solutions. A series of working solutions of the analytes were obtained by diluting the mixed stock solution with 70% aqueous ethanol to the appropriate concentration.

#### 2.6.2 Sample preparation

A total of 0.2 g of the sample was extracted with 10 mL of 70% ethanol (*v/v*) in an ultrasonic water bath for 30 min and then centrifuged at 13000 rpm for 10 min. The supernatant solution was filtered through a 0.22 μm micropore membrane before injection into the UHPLC system. Each sample was analyzed in triplicate.

#### 2.6.3 UHPLC-PDA conditions

The quantitative analysis of 12 phenolic compounds was performed on a UHPLC system (Waters Corporation, USA) coupled with a photodiode array detector (PDA). The analytes were separated on a Waters Acquity HSS T3 column (2.1 mm × 100 mm, 1.8 µm), and the column temperature was maintained at 40°C. The mobile phase consisted of acetonitrile (A) and water containing 0.3% acetic acid (B) with a flow rate of 0.3 mL/min. The following gradient program was used: 0-5 min, 20-25% A; 5-30 min, 25-30% A; 30-62 min, 30-38% A; 62-75 min, 38-95% A; and 75-76 min, 95-20% A. The PDA detector was set to monitor the signal at 278 nm. The sample injection volume was 5.0 μL.

#### 2.6.4 Method validation

The developed UHPLC-PDA method was validated by determining its linearity, limit of detection (LOD), limit of quantification (LOQ), precision (intra- and interday), repeatability, stability and accuracy. The mixed standard containing 12 analytes was diluted with 70% aqueous ethanol to obtain a range of appropriate concentrations for calibration curves, which were constructed by plotting the peak area (y) versus the corresponding concentration (x, μg/ml). The LODs and LOQs were determined at signal-to-noise (S/N) ratios of 3 and 10, respectively. The precision of the intra- and inter-day was tested by performing six replicates of a mixed standard solution within a single day and repeating the experiment on three consecutive days. Repeatability was tested by preparing six replicates of *D. cochinchinensis*. The stability of all analytes was evaluated by injecting samples at 0, 2, 4, 8, 12 and 24 h. The relative standard deviation (RSD) value was used to assess precision, repeatability and stability. The accuracy of the method was determined via a recovery test by spiking an equal amount of standard mixture to a known amount of sample (0.1 g) of *D. cochinchinensis* (n=6). The spiked samples were subsequently extracted and analyzed via the proposed method.

### 2.7 Statistical analysis

The data were analyzed via SPSS 26.0 (SPSS Inc., Chicago, IL, USA). All values are expressed as the means ± SDs of at least three independently performed experiments. The results were considered statistically significant when p<0.05. Figure processing was performed with GraphPad Prism 8.0 software(GraphPad Software, Inc., San Diego, CA, USA).

## 3 Results

### 3.1 Metabolomic analysis

#### 3.1.1 Identification and analysis of differentially abundant metabolites

To better understand the metabolic differences between geographical origins and species, LC–MS/MS was employed to detect CDB metabolites in three different groups: Gdc, Hdsp and Hdca. Mass spectral data were processed by alignment of all data sets from the samples in the CDB group. Metabolite annotation was performed by comparing accurate *m/z* values (**Supplementary Figure S1**). A total of 1237 metabolites were annotated (**Supplementary Table S2**), the most abundant of which were shikimates and phenylpropanoids (49.475%), followed by terpenoids (16.168%), fatty acids (8.892%), polyketides (5.74%) and alkaloids (5.416%) (**Supplementary Figure S2**).

**Figure 1:**
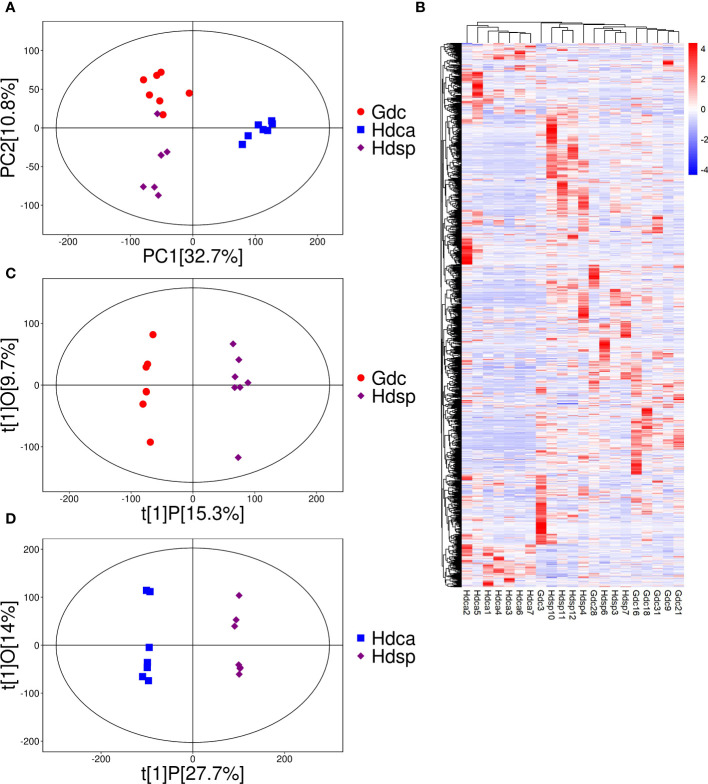
Principal component analysis (PCA) **(A)**, heatmap of hierarchical clustering analysis (HCA) **(B)**, OPLS-DA loading plots **(C, D)** of metabolites detected in different geographical origins and species.

PCA was used as an unsupervised method to study the differences in metabolic profiles between two geographical origins and two species of CDB. The PCA results revealed significant separation of three different groups of samples, which reflected significant differences in metabolite levels in different sources, with 43.5% of the differences explained by the first two principal components (32.7% for PC1 and 10.8% for PC2). In addition, Hdca samples were significantly separated from Hdsp samples in the PCA, whereas the Gdc and Hdsp samples partially overlapped (**Figure 1A**). A correlation analysis of the multisource metabolome revealed strong correlations between the biorepeats within the same group. In different groups of samples, Hdca and Hdsp samples could be distinguished from each other, but this was not true for some Gdc and Hdsp samples, similar to the PCA results (**Supplementary Figure S3**). The heatmap of the hierarchical clustering analysis (HCA) results illustrate the differences in metabolite accumulation among the three groups of samples. These samples were divided into two large clusters, with significant differences between Hdca and Hdsp and Gdc (**Figure 1B**), preliminarily revealing that the difference in metabolites between the two species was greater than the difference between the two geographical origins.

**Figure 2:**
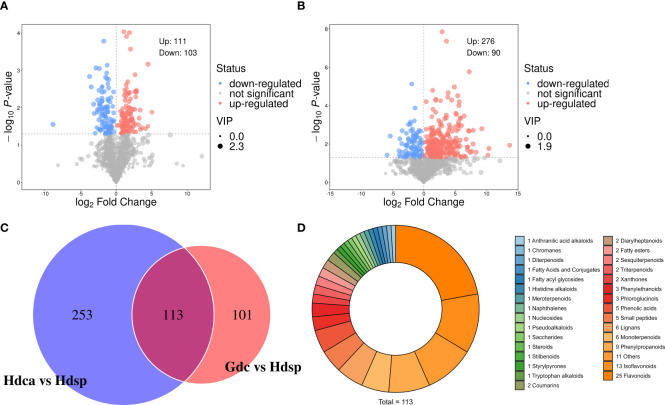
Volcano plots (red, blue and grey dots mean the metabolites were thought to be up-, down-regulated and inapparent.) of differentially accumulated metabolites in different geographical origins **(A)** and different species **(B)**. Venn diagram built to discriminate CDB samples from two geographical origins (Gdc vs Hdsp) and two species (Hdsp vs Hdca) **(C)**. Pie chart illustrating the classification of the 113 potential biomarkers **(D)**.

To investigate the differences in metabolites between different species and different geographical origins of CDB, OPLS‐DA was performed. The Hdsp and Gdc groups were significantly separated on the first principal component, with P1 values of 15.3% and 9.7%, respectively (**Figure 1C**). Hdsp and Hdca were also significantly separated on the first principal component, with P1 values of 27.7% and 14.0%, respectively (**Figure 1D**). The R^2^Y and Q^2^ values of CDB from different origins were 0.96 and 0.59, respectively (**Supplementary Figure S4A**), whereas the R^2^Y and Q^2^ values of the samples from different species were 0.84 and 0.94, respectively (**Supplementary Figure S4B**), both of which did not exceed the true value (horizontal line), implying that there was no overfitting. DAMs were identified via Student’s t test and variable importance in projection (P <0.05; VIP>1). Volcano plots revealed 214 DAMs in samples from different geographical origins (**Figure 2A**), and 366 DAMs in samples from different species (**Figure 2B**), which also suggested that the differences in the metabolome between the two species were greater than the differences between the two geographical origins.

#### 3.1.2 Metabolite biomarkers differentiating geographical origins and species of CDB

When the DAMs between different species and different geographical origins were compared, 113 DAMs were common to the two comparison groups, and 253 and 101 DAMs were specific to Hdsp *vs* Hdca and Gdc *vs* Hdsp, respectively (**Figure 2C**).

The 113 shared compounds could be divided into 31 categories (**Figure 2D**), including 25 flavonoids. For binary logistic regression, the top 10 DAMs from the 113 DAMs (*P* < 0.05, VIP > 1) were selected as candidate combined biomarkers for receiver operating characteristic (ROC) curve analysis. The ROC curve results revealed that these combined biomarkers had excellent sensitivity and high specificity (AUC = 1) (**Supplementary Figure S5**), effectively distinguishing CDB from different geographical origins and species. Therefore, these 10 DAMs can discriminate *D. cochinchinensis* from the Guangxi and Hainan origins and different species of *D. cochinchinensis* and *D. cambodiana*. Additionally, individual ROC analyses of these 10 DAMs also demonstrated high sensitivity and specificity (AUC > 0.96) (**Supplementary Figure S5**), allowing them to be used as biomarkers alone to screen for different geographical origins and species.

**Figure 3:**
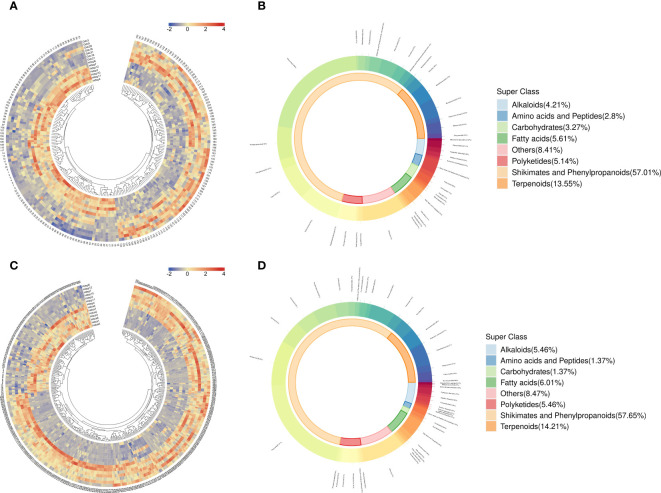
Hierarchical clustering analysis **(A)** (red colors show high abundance, whereas low abundance is presented by blue) and donut plot **(B)** for CDB samples from different geographical origins. Hierarchical clustering analysis **(C)** and donut plot **(D)** for CDB samples from different species. The names of the compounds represented by each number in **Figures 2A, C** are listed in **Supplementary Table S7**.

The differences in metabolites between *D. cochinchinensis* collected from Guangxi and Hainan Provinces were compared (Gdc *vs* Hdsp). The volcano plot (**Figure 2A**) and HCA (**Figure 3A**) revealed that among 214 DAMs between Gdc and Hdsp, 111 were upregulated, with a greater relative abundance in Gdc, and 103 were downregulated, with a greater relative abundance in Hdsp. These findings indicate the promising potential of these metabolites to distinguish CDB from different geographical origins. These DAMs can be broadly classified into 8 categories: shikimates and phenylpropanoids (57.01%), terpenoids (13.55%), fatty acids (5.61%), polyketides (5.14%), alkaloids (4.21%), amino acids and peptides (2.8%), carbohydrates (3.27%) and others (8.41%) (**Figure 3B**). Phenolic compounds, particularly flavonoids and stilbenoids, which are abundant and primary pharmacological components, are the active ingredients in CDB. Therefore, these metabolites were selected for the significance analysis of relative abundance and significance between different geographical origins. A total of 101 unique compounds were identified in the comparative groups. Further screening revealed no unique stilbenoid compounds, whereas there were 22 unique flavonoid compounds. Among these 22 flavonoids, 15 had a relatively high relative abundance in the Gdc samples, whereas 7 had a relatively high relative abundance in the Hdsp samples, indicating that the relative abundance of flavonoids in the Gdc samples was greater than that in Hdsp samples (**Supplementary Table S3**). Additionally, oppositin and spinoflavanone a showed more than a 10-fold difference in relative abundance between the two groups, confirming their role as crucial biomarkers for tracking CDB from different origins.

Two species of *D. cochinchinensis* and *D. cambodiana* growing in Hainan Province were compared to identify differences. A total of 366 compounds were identified as potentially DAMs between Hdca and Hdsp. The volcano plot (**Figure 2B**) and HCA (**Figure 3C**) illustrate that among these metabolites, 276 were upregulated, with a greater relative abundance in Hdsp. Additionally, 90 metabolites were downregulated, exhibiting a greater relative abundance in Hdca. These findings indicate the promising potential of these metabolites in distinguishing CDB from *D. cochinchinensis* and *D. cambodiana*. These DAMs can be broadly classified into 8 categories: shikimates and phenylpropanoids (57.65%), terpenoids (14.21%), fatty acids (6.01%), polyketides (5.46%), alkaloids (5.46%), amino acids and peptides (1.37%), carbohydrates (1.37%) and others (8.47%) (**Figure 3D**). Additionally, because flavonoids and stilbenoids are the main active ingredients of CDB, these metabolites were selected for the analysis of their relative abundance and significance between the different species. In the comparisons between the different CDB species, there were 253 unique compounds, including 53 flavonoids and 9 stilbenoids. Among these 53 flavonoids, 28 had greater relative abundance in Hdsp, and another 15 in Hdca; of the 10 stilbenoids, 8 were more abundant in Hdsp and 1 was more abundant in Hdca, indicating higher relative abundance of these two main compound classes in Hdsp samples (**Supplementary Table S4**). In addition, the relative abundance of 19 DAMs, including naringin, sanggenol l, 6-hydroxy-2-(4-methoxyphenyl)-4h-chromen-4-one, artonol c, (2s)-4’-hydroxy-5,7,3’-trimethoxyflavan, 7-methoxyflavonol, brosimacutin i, farrerol, 5,7-dihydroxy-2-(4-hydroxyphenyl)-8-[3,4,5-trihydroxy-6-(hydroxymethyl)oxan-2-yl]-6-(3,4,5-trihydroxyoxan-2-yl)chromen-4-one, 7-hydroxy-2-(4-hydroxy-3,5-dimethoxyphenyl)-5-[(2s,3r,4s,5s,6r)-3,4,5-trihydroxy-6-(hydroxymethyl)oxan-2-yl]oxychromen-4-one, sempervirenoside b, robinin, didymin, 2’-methoxyflavone, cardamonin, angoletin, trans-resveratrol, pinosylvin and 3,4,5-trihydroxystilbene, exhibited more than a 10-fold difference between the two groups, and these metabolites were identified as important biomarkers for tracking different species of CDB.

#### 3.1.3 Metabolic pathway analysis

**Figure 4:**
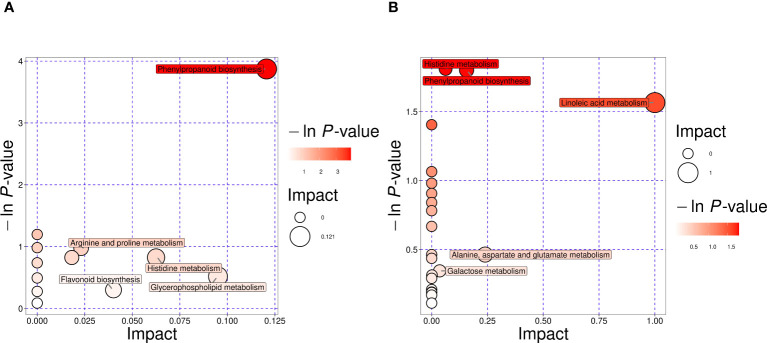
Pathway analysis of differential metabolites. Gdc vs Hdsp **(A)**. Hdsp vs. Hdca **(B)**. Each bubble represents one pathway, the abscissa and the size of the bubble reflect the influencing factors of the pathway.

To elucidate the metabolic activities in CDB obtained from different geographical origins and species, we mapped the DAMs to KEGG pathways. After comprehensive consideration, the significant pathways were marked with large bubbles and dark colors. Phenylpropanoid biosynthesis, arginine and proline metabolism, histidine metabolism, flavonoid biosynthesis, and glycerophospholipid metabolism were the main pathways enriched between Gdc and Hdsp, and the results are shown in **Figure 4A**. In addition, pathways such as histidine metabolism; phenylpropanoid biosynthesis; linoleic acid metabolism; alanine, aspartate and glutamate metabolism and galactose metabolism were enriched predominantly between Hdsp and Hdca (**Figure 4B**). Among these major enrichment pathways, histidine metabolism and phenylpropanoid biosynthesis were two pathways common to both comparison groups. It can be inferred that the aforementioned biosynthetic pathways act as critical pathways and play a significant role in the quality variation in CDB from different geographical origins and species.

To explore how geographical origins and species quantitatively affect the chemical components of CDB, 12 phenolic compounds, namely, p-hydroxybenzyl alcohol, resveratrol, 4H-1-benzopyran-4-one,2,3-dihydro-5-hydroxy-3-[(4-hydroxyphenyl)methyl]-7-methoxy-, liquiritigenin, pinostilbene, (3R)-5,7-dihydroxy-3-(4-hydroxybenzyl)-2,3-dihydro-4H-chromen-4-one, 4H-1-benzopyran-4-one,2,3-dihydro-3,5,7-trihydroxy-3-[(4-methoxyphenyl)methyl]-,(R)-, loureirin A, loureirin B, loureirin D, loureirin C, and pterostilbene, were selected as the analytes for UHPLC-PDA analysis. These chemicals were not only identified as major chemical components but also reported to be the major bioactive chemical components in CDB.

### 3.2 UHPLC-PDA analysis

#### 3.2.1 UHPLC-PDA conditions

In this study, the extraction conditions (extraction solvent, extraction time) and chromatographic conditions (mobile phase, gradient program) were optimized for the 12 phenolic compounds because of their different chemical properties.

To achieve the optimal extraction conditions, the extraction solvent (50%, 70% and 100% ethanol, *v/v*) and extraction time (15, 30, 60, and 90 min) were investigated. Aqueous ethanol was found to be more suitable for the samples since it provided the highest amounts of the target analytes. The extraction efficiency did not significantly increase with ethanol concentrations above 70%. Furthermore, the extraction time was also investigated, and the extraction efficiency did not significantly increase when the extraction time exceeded 60 min (**Supplementary Figure S6**).

To separate the compounds and improve the sensitivity, the chromatographic and detector conditions were systemically optimized. First, gradient elution was chosen because of the wide polarity range of the 12 analytes selected. The most suitable elution solvents were acetonitrile (A) and 0.3% acetic acid. The proportions of acetonitrile and acetic acid, as well as the gradient elution time, were carefully adjusted based on the various schemes outlined in **Supplementary Table S5**. Remarkably, Scheme 4 demonstrated exceptional separation results in a relatively short timeframe. Consequently, under the established optimal conditions, a representative chromatogram (**Supplementary Figure S7**) was obtained, effectively illustrating the successful separation and elution of the target compounds.

#### 3.2.2 Method validation

The optimized UHPLC-PDA method was validated by evaluating its linearity, LOD, LOQ, precision, repeatability, stability and accuracy. The results are summarized in **Table 1**. Notably, excellent linearity was observed in the regression equations of all analytes, with a coefficient of determination (R^2^) ≥ 0.9983. The LODs and LOQs of the 12 analytes ranged from 0.021 to 0.031 μg/mL and 0.069 to 0.089 μg/mL, respectively. The overall RSDs of the intraday and interday variations were not greater than 3.47% and 4.92%, respectively. The repeatability and stability did not exceed 2.99% and 2.82%, respectively. The recovery of the method was determined to be between 93.70% and 104.91%, with RSDs (%) ranging from 1.06 to 5.16 (**Table 2**). All these results showed that the established method has good linearity, precision, stability, reproducibility and accuracy and can be used for the quantification of 12 phenolic compounds in CDB.

**Table 1:** Calibration curves,testrange,LOD,LOQ,precision,stability and repeatability for the 12 analytes.

Analytes	Linearrange (μg·mL^−1^)	Calibration curves	R^2^	LOQ (μg·mL^−1^)	LOD (μg·mL^−1^)	Precision(RSD, %)		Stability (RSD,%)	Reproducibility (RSD,%,n=6)
						intraday (n=6)	Interday (n=6)		
p-hydroxybenzyl alcohol	0.67~33.33	y=16859x+2199.4	0.9983	0.083	0.027	2.04	4.92	1.73	2.99
resveratrol	0.6~30.00	y=109,961x-14,874.5	1.0000	0.072	0.024	0.21	0.98	1.15	2.17
liquiritigenin	0.53~26.67	y=99799x+4093.5	1.0000	0.070	0.021	0.45	3.11	0.50	2.17
4H-1-benzopyran-4-one,2,3-dihydro-5-hydroxy-3-[(4-hydroxyphenyl) methyl]-7-methoxy-	0.78~39.00	y=121263x+4635.3	1.0000	0.087	0.031	3.01	4.50	0.62	2.60
loureirin D	0.70~35.00	y=81439x+1910.8	0.9999	0.088	0.028	0.22	0.86	2.70	1.19
loureirin C	0.66~33.00	y=88,723x+939.6	1.0000	0.081	0.026	0.19	1.46	2.45	1.68
(3R)-5,7-Dihydroxy-3-(4-hydroxybenzyl)-2,3-dihydro-4H-chromen-4-one	0.62~31.00	y=97163x+2408.9	0.9999	0.073	0.025	0.52	3.81	2.62	2.25
pinostilbene	0.64~32.00	y=105306x-25996.7	0.9999	0.074	0.026	0.57	2.84	1.06	2.98
4H-1-benzopyran-4-one,2,3-dihydro-3,5,7-trihydroxy-3-[(4-methoxyphenyl) methyl]-,(R)-	0.59~29.33	y=88730x+2171.2	1.0000	0.069	0.023	3.47	4.27	2.49	2.08
loureirin A	0.77~38.33	y=110474x+2046.7	1.0000	0.089	0.031	2.44	4.16	2.82	2.44
loureirin B	0.77~38.33	y=95,204x-220.9	1.0000	0.086	0.031	1.09	3.12	2.75	2.46
pterostilbene	0.64~32.00	y=96782x-35754.5	0.9998	0.079	0.026	2.58	2.51	2.65	2.13

**Table 2:** Experimental results of sample addition recovery rate (n=6).

Analytes	Original (mg)	Added (mg)	Found (mg)	Recovery (%)	Recovery (%)	RSD (%,n=6)
p-hydroxybenzyl alcohol	0.0034	0.0169	0.0211	104.60	104.91	1.85
	0.0034	0.0169	0.0210	103.90		
	0.0034	0.0169	0.0206	101.74		
	0.0034	0.0169	0.0213	106.02		
	0.0034	0.0169	0.0215	107.21		
	0.0034	0.0169	0.0213	106.01		
resveratrol	0.0366	0.0160	0.0533	104.62	104.85	1.69
	0.0366	0.0160	0.0532	104.16		
	0.0366	0.0160	0.0532	103.83		
	0.0366	0.0160	0.0532	103.91		
	0.0366	0.0160	0.0532	104.13		
	0.0366	0.0160	0.0539	108.42		
liquiritigenin	0.0016	0.0136	0.0155	101.91	100.58	1.26
	0.0016	0.0136	0.0151	99.03		
	0.0016	0.0136	0.0154	101.04		
	0.0016	0.0136	0.0153	100.37		
	0.0016	0.0136	0.0151	99.23		
	0.0016	0.0136	0.0155	101.90		
4H-1-benzopyran-4-one,2,3-dihydro-5-hydroxy-3-[(4-hydroxyphenyl)methyl]-7-methoxy-	0.0022	0.0218	0.0230	95.23	93.70	1.34
	0.0022	0.0218	0.0225	93.00		
	0.0022	0.0218	0.0227	93.75		
	0.0022	0.0218	0.0226	93.30		
	0.0022	0.0218	0.0223	91.93		
	0.0022	0.0218	0.0230	95.01		
loureirin D	0.0094	0.0152	0.0250	102.57	101.59	1.61
	0.0094	0.0152	0.0247	100.30		
	0.0094	0.0152	0.0249	101.52		
	0.0094	0.0152	0.0247	100.32		
	0.0094	0.0152	0.0247	100.44		
	0.0094	0.0152	0.0253	104.38		
loureirin C	0.0170	0.0170	0.0337	98.31	97.87	1.49
	0.0170	0.0170	0.0335	96.77		
	0.0170	0.0170	0.0338	98.48		
	0.0170	0.0170	0.0334	96.41		
	0.0170	0.0170	0.0335	96.96		
	0.0170	0.0170	0.0341	100.31		
(3R)-5,7-Dihydroxy-3-(4-hydroxybenzyl)-2,3-dihydro-4H-chromen-4-one	0.0151	0.0156	0.0310	101.63	97.79	2.44
	0.0151	0.0156	0.0301	96.26		
	0.0151	0.0156	0.0303	97.09		
	0.0151	0.0156	0.0300	95.55		
	0.0151	0.0156	0.0302	96.45		
	0.0151	0.0156	0.0307	99.79		
pinostilbene	0.0115	0.0172	0.0282	97.05	101.43	2.77
	0.0115	0.0172	0.0286	99.49		
	0.0115	0.0172	0.0295	104.19		
	0.0115	0.0172	0.0291	102.31		
	0.0115	0.0172	0.0289	101.25		
	0.0115	0.0172	0.0295	104.26		
4H-1-benzopyran-4-one,2,3-dihydro-3,5,7-trihydroxy-3-[(4-methoxyphenyl)methyl]-,(R)-	0.0022	0.0143	0.0159	95.47	93.72	1.24
	0.0022	0.0143	0.0156	93.53		
	0.0022	0.0143	0.0156	93.90		
	0.0022	0.0143	0.0155	93.02		
	0.0022	0.0143	0.0154	92.06		
	0.0022	0.0143	0.0157	94.34		
loureirin A	0.0042	0.0228	0.0265	97.59	98.14	1.06
	0.0042	0.0228	0.0264	97.11		
	0.0042	0.0228	0.0268	98.88		
	0.0042	0.0228	0.0266	98.10		
	0.0042	0.0228	0.0265	97.33		
	0.0042	0.0228	0.0270	99.84		
loureirin B	0.0505	0.0196	0.0702	100.74	102.42	2.21
	0.0505	0.0196	0.0706	102.40		
	0.0505	0.0196	0.0707	103.27		
	0.0505	0.0196	0.0702	100.28		
	0.0505	0.0196	0.0704	101.36		
	0.0505	0.0196	0.0714	106.47		
pterostilbene	0.0084	0.0160	0.0235	94.08	103.80	5.16
	0.0084	0.0160	0.0249	103.18		
	0.0084	0.0160	0.0250	103.35		
	0.0084	0.0160	0.0253	105.31		
	0.0084	0.0160	0.0256	107.31		
	0.0084	0.0160	0.0260	109.60		

#### 3.2.3 Quantitative results

The developed UHPLC-PDA method was applied to simultaneously determine the amount of 12 phenolic compounds in 51 batches of CDB samples collected from different sources (**Supplementary Table S6**).

Among the 51 batches of CDB samples, the contents of the 12 analytes ranged from 0.01 to 21.05 mg/g. Notably, loureirin B, loureirin C, p-hydroxybenzyl alcohol and resveratrol had the highest average contents of 3.63, 3.48, 3.21 and 3.12 mg/g, respectively, suggesting a potentially crucial role for these four phenolic compounds in CDB. Furthermore, loureirin B has been used as a chemical marker for quality control with CDB. Among the 51 samples tested, only 13 batches of loureirin B met the 0.4% limit. More precisely, only 5 of 31 batches of Gdc samples were qualified, 8 of 12 batches of Hdsp samples were qualified, and none of the Hdca samples met the criteria. In other words, almost 75% of the samples did not meet the expected quality standards, and the Hdca samples have a particularly prominent quality issue, highlighting the need for further investigation into the reasons and implementation of appropriate measures to ensure product quality and safety.

#### 3.2.4 Quantitative analysis of CDB from differentiating geographical origins and species

**Figure 5:**
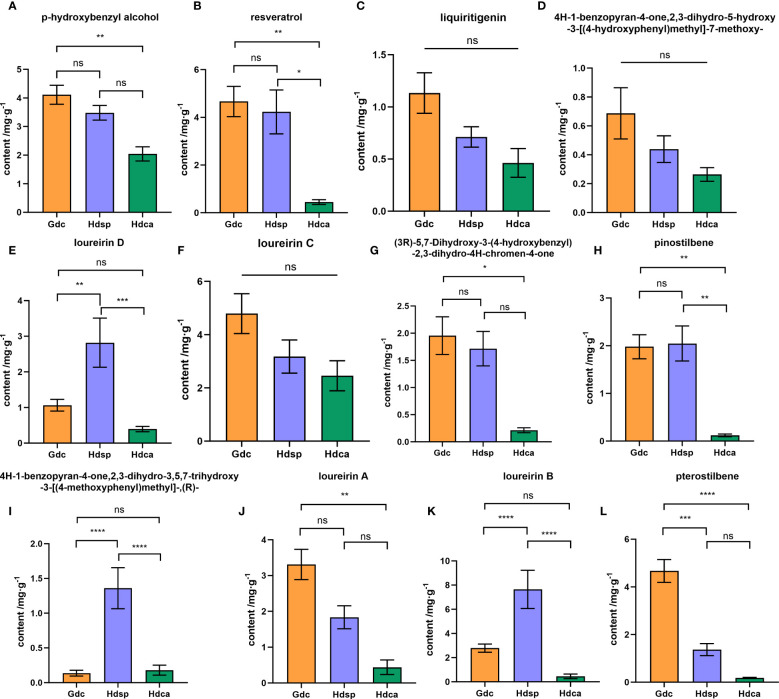
Results of quantitative analysis of 12 phenolic components **(A-L)** in Chinese dragon's blood samples from Gdc (D. cochinchinensis from Guangxi Province), Hdsp (D. cochinchinensis from Hainan Province) and Hdca (D. cambodiana from Hainan Province). *P < 0.05, **P < 0.01, ***P < 0.001, ****P < 0.0001, ns means no significance. p-hydroxybenzyl alcohol **(A)**, resveratrol **(B)**, liquiritigenin **(C)**, 4H-1-benzopyran-4-one,2,3-dihydro-5-hydroxy-3-[(4-hydroxyphenyl)methyl]-7-methoxy- **(D)**, loureirin D **(E)**, loureirin C **(F)**, (3R)-5,7-dihydroxy-3-(4-hydroxybenzyl)-2,3-dihydro-4H-chromen-4-one **(G)**, pinostilbene **(H)**, 4H-1-benzopyran-4-one,2,3-dihydro-3,5,7-trihydroxy-3-[(4-methoxyphenyl)methyl]-,(R)- **(I)**, loureirin A **(J)**, loureirin B **(K)**, and pterostilbene **(L)**.

Notable variations were observed in the comparative analysis of 12 phenolic compounds in the CDB samples obtained from different geographical origins. The Hdsp samples presented relatively high contents of loureirin D, pinostilbene, 4H-1-benzopyran-4-one,2,3-dihydro-3,5,7-trihydroxy-3-[(4-methoxyphenyl) methyl]-, (R)-, and loureirin B. Conversely, the Gdc samples presented higher contents of the remaining eight components. Statistical analysis (P < 0.05) revealed significant difference between the two geographical origins in China for loureirin D, 4H-1-benzopyran-4-one,2,3-dihydro-3,5,7-trihydroxy-3-[(4-methoxyphenyl) methyl]-, (R)-, loureirin B, and pterostilbene (**Figure 5**).

Furthermore, when comparing the CDB samples from the different species, significant differences were observed in the average contents of the 12 phenolic compounds. Specifically, the contents of all 12 compounds were greater in Hdsp than in Hdca. In addition, the analytical results (**Figure 5**) revealed that the contents of components such as resveratrol, loureirin D, pinostilbene, 4H-1-benzopyran-4-one,2,3-dihydro-3,5,7-trihydroxy-3-[(4-methoxyphenyl) methyl]-, (R)- and loureirin B significantly differed (P < 0.05).

## 4 Discussion

CDB is a rare and valuable traditional medicine that is found mainly in Hainan and Guangxi, China, and is extracted from the fat-containing wood of *D. cochinchinensis* and *D. cambodiana*. Previous studies have shown that the main components of CDB are phenolic compounds with antibacterial, anti-inflammatory, analgesic, antiplatelet aggregation, blood circulation and epidermal repair properties (Zheng et al., 2021). The intrinsic quality of TCM is closely associated with its secondary metabolites. The types, quantities and activities of these secondary metabolites are in turn affected by factors such as the growth environment, climatic conditions and species of medicinal materials (Guo et al., 2023). Therefore, it is highly important to study and elucidate the influence of these external factors on the secondary metabolites of TCM to ensure and improve its quality. In this study, LC–MS/MS technology combined with UHPLC–PDA quantitative analysis was used to conduct in-depth metabolomics analysis to systematically study the differences in the metabolite composition and content of CDB from different geographical origins and species.

In-depth analyses of the compounds shared by the two comparison groups of CDB from different origins and species were performed via Venn diagrams, and we identified 10 metabolites with significant differences in abundance. Furthermore, these 10 shared metabolites were analyzed via ROC curve analysis, and they exhibited very high AUC values (>0.96) in distinguishing between different groups of samples, whether analyzed in combination or individually, which fully demonstrated their excellent performance in identifying and distinguishing between different samples. The results of the available pharmacological studies indicate that coniferyl alcohol has significant anti-inflammatory and analgesic activities and can act as a signaling agent to modulate the phenylpropane pathway in several ways (Wang et al., 2021; Guan et al., 2022). 4-acetamidobutanoate is a product of the urea cycle, and its levels increase with renal dysfunction and are altered in inflammatory disorders such as rheumatoid arthritis. In addition, it is involved in several key metabolic pathways including arginine and proline metabolism, histidine metabolism, lysine degradation, and phenylalanine metabolism (Zhang K. et al., 2023; Kui et al., 2022). Artoindonessianin a exhibited cytotoxic activity against murine leukemia (Jamil et al., 2014). The compound (1E,4Z,6E)5-hydroxy-1-7-bis(4-hydroxy-3-methoxyphenyl)hepta-1,4,6-trien-3-one, which is commonly known as curcumin (enol form), possesses antioxidant, anti-inflammatory, antiviral, antibacterial, antihypertensive, and insulin sensitizer activities; is cytotoxic to cancer cell lines; and can modulate apoptosis (Arenaza-Corona et al., 2023). The unique properties and wide range of biological activities of these compounds reveal their great potential and broad prospects for the future medical research and clinical applications of CDB.

### 4.1 Influence of different origins on *D. cochinchinensis*

Flavonoids are the major components of CDB and are associated with the phenylpropanoid biosynthetic pathway (Nabavi et al., 2020; Liu W. et al., 2021; Xu et al., 2022). Through metabolomics analyses, 2 significantly different flavonoid metabolites were identified as ideal chemomarkers for distinguishing CDB from different origins, with spinoflavanone a showing higher relative abundance in Gdc and oppositin showing higher relative abundance in Hdsp. These two DAMs may serve as useful quality markers for the identification of Gdc and Hdsp.

Among several pathways associated with DAMs enrichment between Gdc and Hdsp, the phenylpropanoid pathway and flavonoid biosynthesis are vital pathways for the production of important secondary metabolites (Petrussa et al., 2013; Du et al., 2022). During the formation of CDB, enzymes in the phenylpropanoid pathway are induced to metabolize phenylalanine to loureirin A and loureirin B, which are the main active ingredients in CDB (Su et al., 2020). Histidine metabolism is not only involved in the plant response to environmental stress and signal transduction but also acts as a chelator and transporter of metal ions, helping plants maintain metal ion homeostasis in different environments (Stepansky and Leustek, 2006). Arginine metabolism helps balance N availability for anabolic processes in a fluctuating environment (Kiss et al., 2023). Proline biosynthesis and accumulation are common responses to unfavorable environments in many plants (Yan et al., 2021). The increased metabolism of arginine and proline promotes root development, increases plant tolerance to osmotic stress, and improves crop resistance to salt stress. These findings suggest that under different geographic environments *D. cochinchinensis* may adapt to environmental changes by fine-tuning these key metabolic pathways to maintain a balance between their growth and survival.

Secondary metabolites are more affected by external stressors, and their metabolic profiles can vary depending on their growth environment even in genetically identical plants (Erb and Kliebenstein, 2020; Yoon et al., 2022). Quantitative analysis revealed that 8 of the 12 phenolic compounds were more abundant in Gdc group than in Hdsp, and the other 4 were more abundant in Hdsp. The difference in phenolic compound content is considered important because each compound has different biological activities (Zhao et al., 2018; Gu et al., 2019). When CDB is used for pharmacological purposes, its purpose may vary depending on the content of phenolic compounds. The main phenolic compounds have been extensively studied for their biological activity. According to the literature, *p*-hydroxybenzyl alcohol, resveratrol, loureirin A, loureirin B, loureirin D, pinostilbene, 4H-1-benzopyran-4-one,2,3-dihydro-3,5,7-trihydroxy-3-[(4-methoxyphenyl)methyl], (R)- and pterostilbene have antibacterial (Zheng et al., 2021), ischemic stroke (Jiang et al., 2020), antioxidant (Hu et al., 2020; Liu Y. S. et al., 2023), analgesic (Wan et al., 2019), antiaging (Allen et al., 2018) and anticancer (Chen et al., 2020) activities. These findings indicate that the different components have multiple biological activities and are source dependent. Changes in these components result in differences in nutritional or pharmacological functions that are related to quality. Therefore, these data can be used to select the origin most suitable for the expected activity.

### 4.2 Differences between the species of *D. cochinchinensis* and *D. cambodiana*

Species genetically determine the differences in the types and contents of plant metabolites, resulting in differences in clinical effects (Xiao et al., 2019). Using metabolomics approaches, 16 flavonoid metabolites were found to exhibit significant differences in abundance between the Hdca and Hdsp groups, among which only naringin showed higher relative abundance in Hdca. Naringin possesses antioxidant, neuroprotective, anti-inflammatory, antiapoptotic, antiulcer, antiosteoporotic, and anticancer properties (Ravetti et al., 2023). The remaining 15 flavonoids presented higher relative abundances in Hdsp, among them, didymin has anticancer, antioxidant, anti-inflammatory, and neuroprotective, hepatoprotective, and cardiovascular effects (Yang et al., 2023). Farrerol has therapeutic effects on pathological conditions such as cancer, muscular dystrophy, inflammation, microbial infections, and oxidative stress (He et al., 2023). Cardamonin has significant anticancer, anti-inflammatory, and antioxidant activities and neuroprotective effects and can attenuate cerebral ischemia/reperfusion injury through activation of the HIF-1α/VEGFA pathway (Ni et al., 2022). Robinin has significant anti-inflammatory, analgesic and antitumor effects (Zhang W. et al., 2023). Sanggenol l possesses a wide range of biological activities such as neuroprotective, anti-inflammatory, and antitumor effects (Fu et al., 2024). Combretastatin A4 is a potent mitotic inhibitor of a variety of tumor cells that inhibits mitosis in a variety of tumor cells, including multidrug-resistant cancer cell lines. It also acts on proliferative endothelial cells and ruptures tumor blood vessels (Yang et al., 2022). Natural stilbenoids are secondary metabolites produced by plants to protect themselves against stressful conditions such as UV irradiation, overheating and fungal or bacterial infections (Medrano-Padial et al., 2021). Compared with the Hdca and Hdsp groups, three significantly different stilbenoid metabolites, trans-resveratrol, pinosylvin, and 3,4,5-trihydroxystilbene, presented greater relative abundances. Among these metabolites, resveratrol, often found in its trans form, possesses pharmacological activities, such as anticancer, anti-inflammatory, cardiovascular protection, and antioxidant effects, and inhibits platelet aggregation (Basholli-Salihu et al., 2016). Pinosylvin possesses a variety of biological properties, such as antimicrobial, anti-inflammatory, anticancer, antioxidant, neuroprotective and antiallergenic effects (Bakrim et al., 2022). Thus, our results suggest that differences in both the composition and abundance of flavonoids and stilbenoids may explain the variation in therapeutic effects observed in herbs from different species. These discriminating metabolites may be useful quality markers for differentiating *D. cochinchinensis* and *D. cambodiana*.

Among the several pathways associated with DAMs enrichment between Hdsp and Hdca, amino acid metabolism is crucial for plant growth and stress resistance (Huang and Jander, 2017). Among them, alanine, aspartate and glutamate metabolism are resistant to pathogens and provide nitrogen for growth when exposed to external stresses, reducing the toxic effects of nitrate in crops (Martinelli et al., 2007). In addition, alanine metabolism plays an important role in the synthesis and defense of the cell wall (Qiao et al., 2018). Furthermore, galactose metabolism plays a coordinating role in cellular metabolism. These differences in metabolic pathways reflect the diversity of genetic backgrounds, environmental adaptations and metabolic requirements of different species, providing an important basis for understanding the uniqueness of different species of CDB.

Quantitative analysis based on UHPLC-PDA revealed that the content of 12 phenolic compounds in the Hdca samples was consistently lower than that in the Hdsp samples. Owing to the differences in pharmacological activities corresponding to different metabolite types and contents, it is inferred that CDB extracts from Hdsp possesses more potent pharmacological activities. Therefore, it is highly important to distinguish CDB samples from different sources in practical applications to ensure the consistency of efficacy and batch reproducibility.

However, importantly, relying solely on component concentrations is insufficient to draw definitive conclusions regarding the quality of medicinal materials such as CDB. Pharmacological experiments are essential to further validate the analytical findings and provide a more comprehensive understanding of the therapeutic.

## 5 Conclusion

In this study, plant metabolomics approaches (LC-MS/MS) combined with a developed UHPLC-PDA method were used to systematically investigate the differences of CDB from different geographical origins and species. The results revealed that origin and species had significant effects on the chemical composition and content of CDB, with species showing particularly pronounced differences. Comprehensive metabolite analysis and quantitative assessment revealed that *D. cochinchinensis* exhibits outstanding performance in compound diversity, relative abundance, and content of phenolic compounds, making it an ideal source of CDB. Furthermore, considering the significant potential of *D. cambodiana*, additional research is necessary to fully explore its considerable potential.

## Data Availability

The original contributions presented in the study are included in the article/**Supplementary Material**. Further inquiries can be directed to the corresponding authors.
