# Supplementary material for: Metabolomics and quantitative analysis to determine differences in the geographical origins and species of Chinese dragon’s blood
Source: Front Plant Sci. 2024 Sep 18;15:1427731. doi: 10.3389/fpls.2024.1427731 (PMC11445005; doi:10.3389/fpls.2024.1427731)
Supplement: Supplementary file 1 [file DataSheet1.docx]

Supplementary Material

Metabolomics and quantitative analysis to determine differences in the geographical origins and species of Chinese dragon's blood

First Author*, Xiuting Sun

*** Correspondence:** Xiangsheng Zhao: [xiangshengzhao@hotmail.com;](mailto:xiangshengzhao@hotmail.com;) Xinquan Yang: [xqyang@implad.ac.cn](mailto:xqyang@implad.ac.cn)

# 1 Supplementary Figures and Tables

## 1.1 Figures


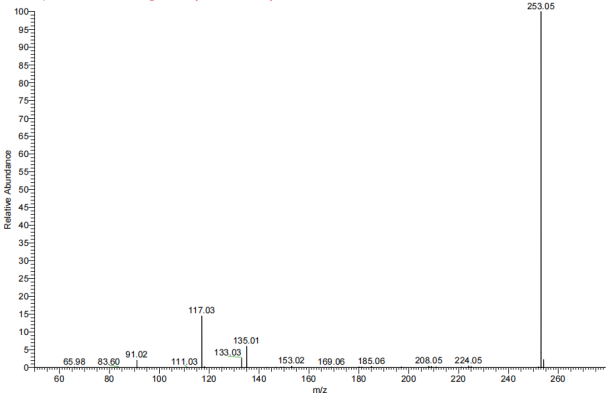

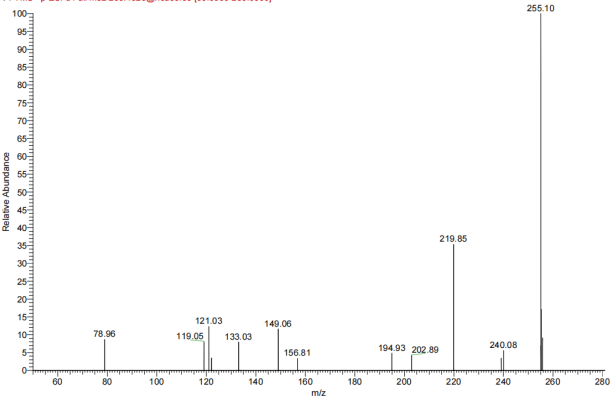


**B**

**A**


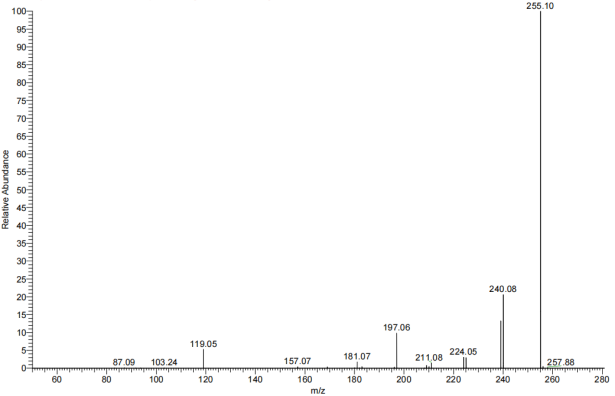

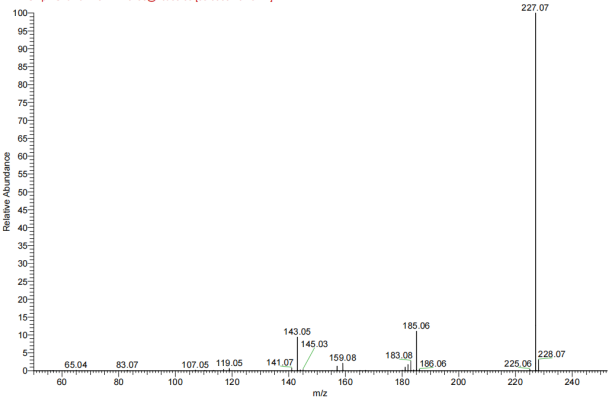


**D**

**C**

**Figure S1.** The MS^2^ spectra of the representative metabolites. 7,4'-dihydroxyflavone (A), Liquiritigenin (B),trans-pterostilbene(C), trans-resveratrol (D).

**
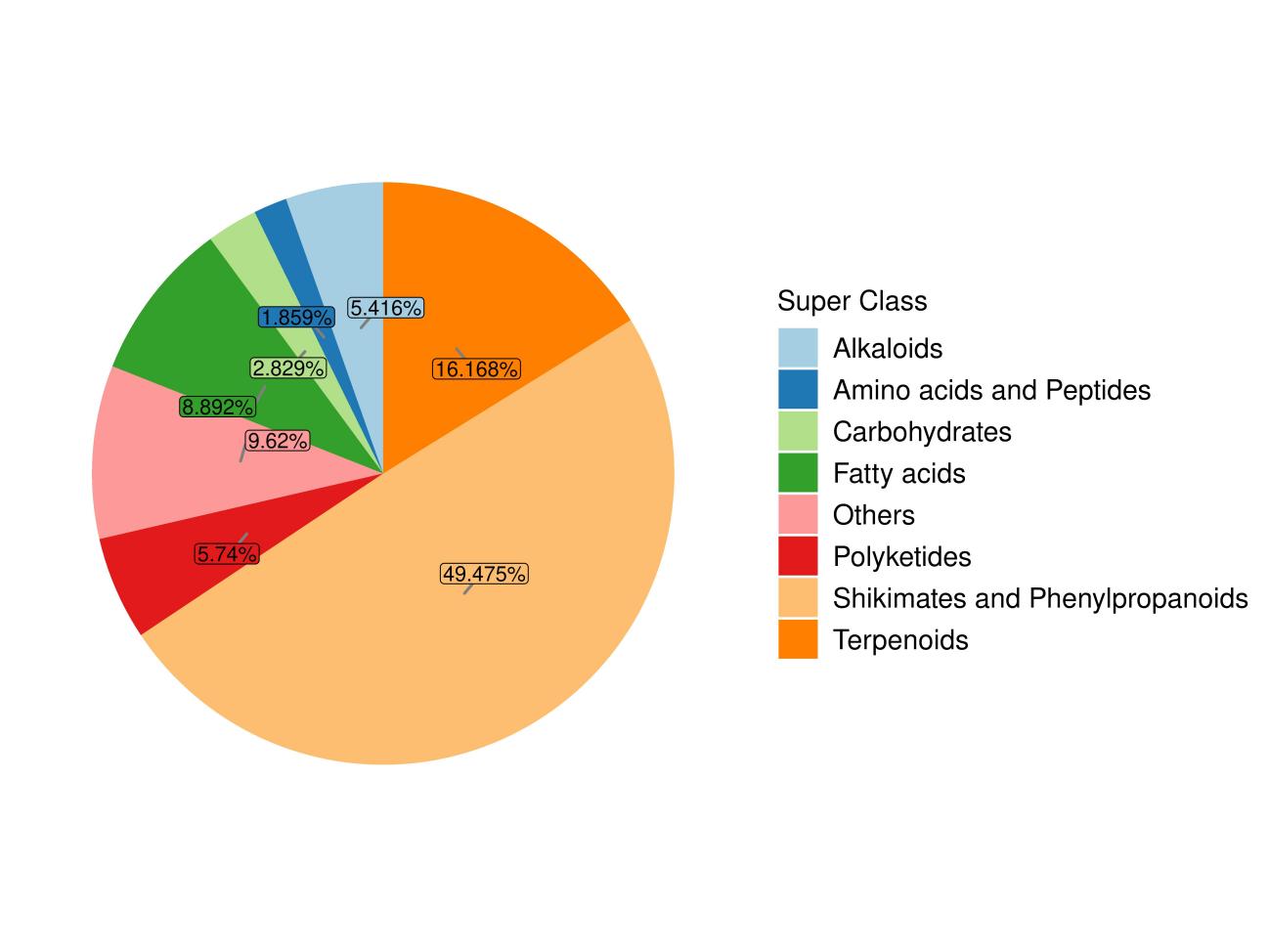
**

**Figure S2.** Pie charts showing the classification and proportion of annotated metabolites.

**
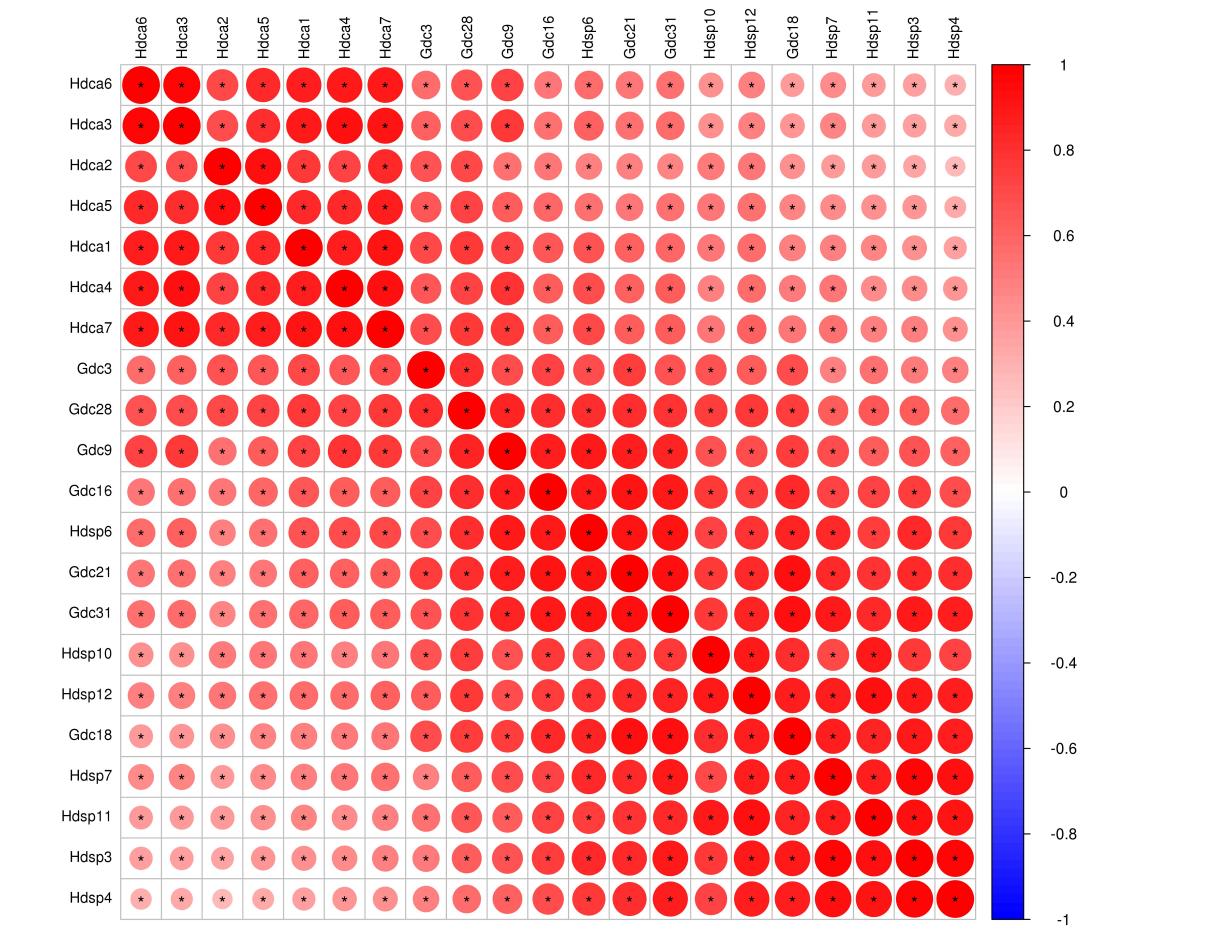
**

**Figure S3.** correlation analysis of metabolites detected in different geographical origins and species.

**
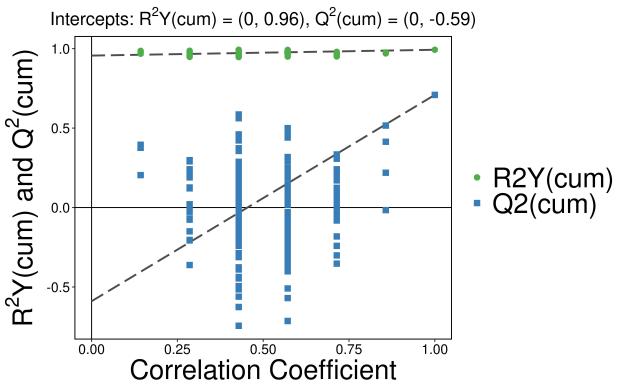

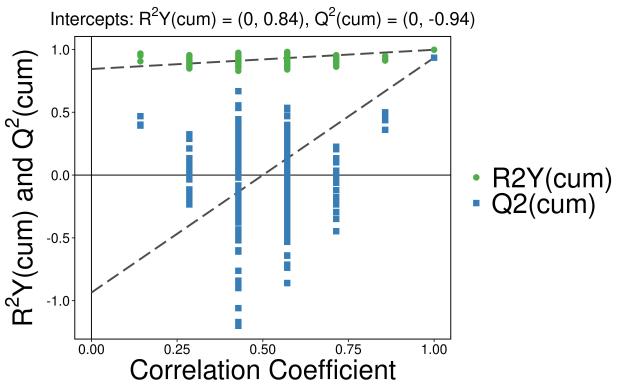
**

**B**

**A**

**Figure S4.** 200 permutations test plots for the 21 samples of CDB from different geographical origins (Gdc and Hdsp) (A) and species (Hdsp and Hdca) (B).

**
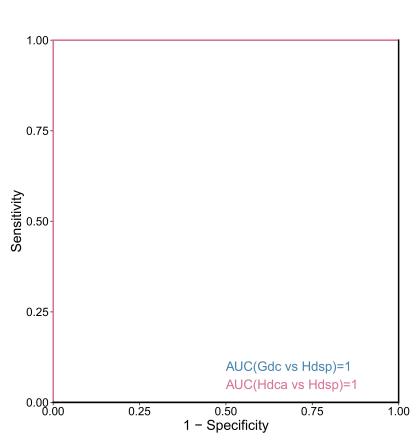

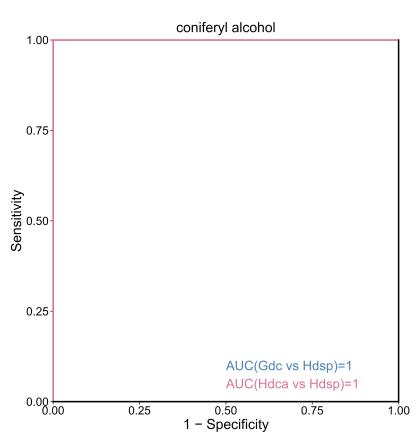

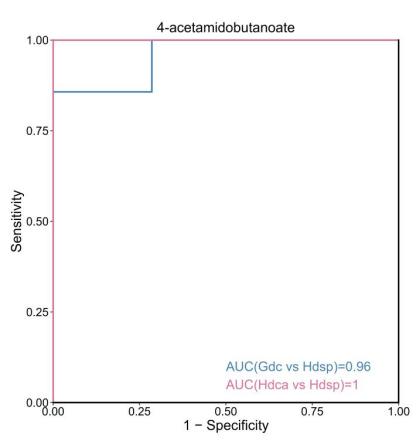

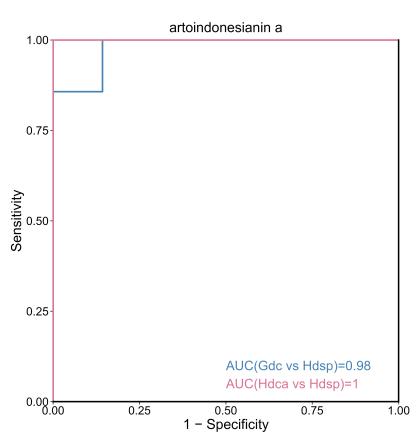

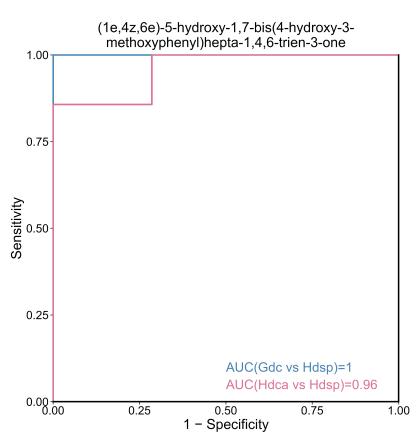

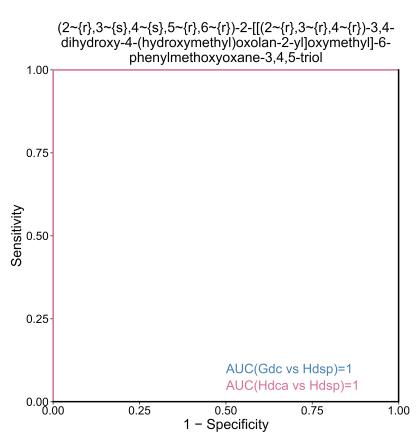

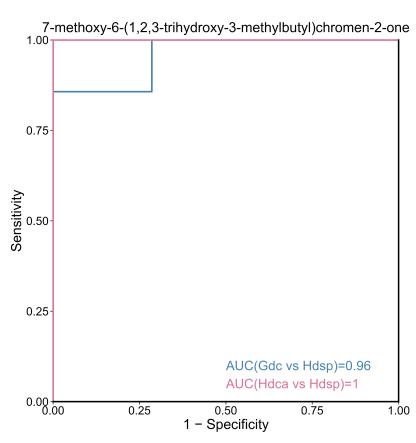

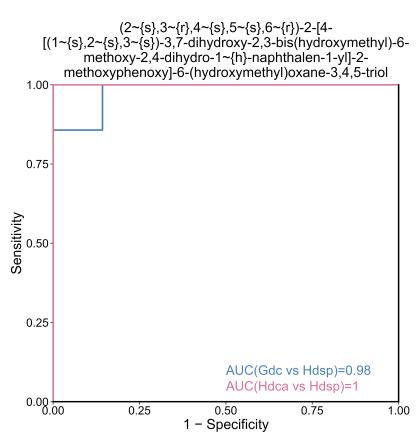

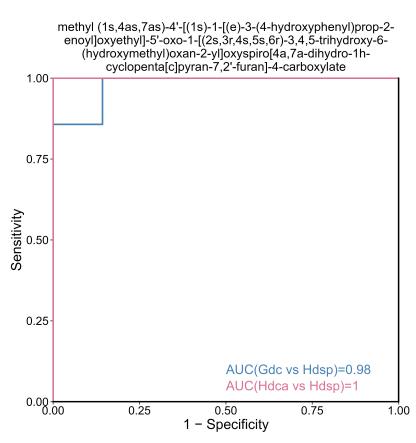
**

**
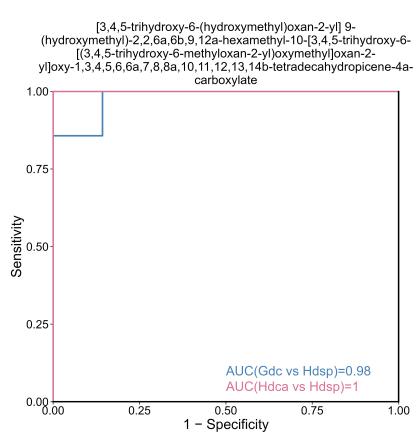

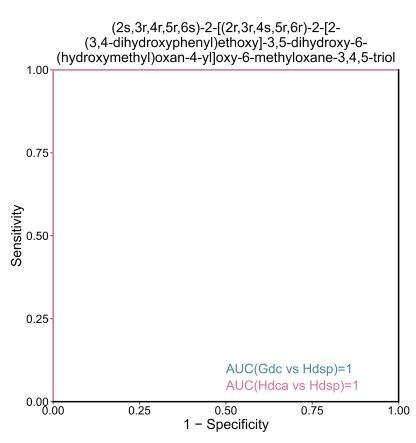
**

**Figure S5.** Assessment of potential biomarkers based on receiver operating characteristic (ROC) curves.

AUC=0.5, it was the same as the follower guess, the model had no predictive value; 0.5 < AUC < 1, it was better than random guessing, and the model had predictive value; AUC = 1, the prediction model made perfect predictions.


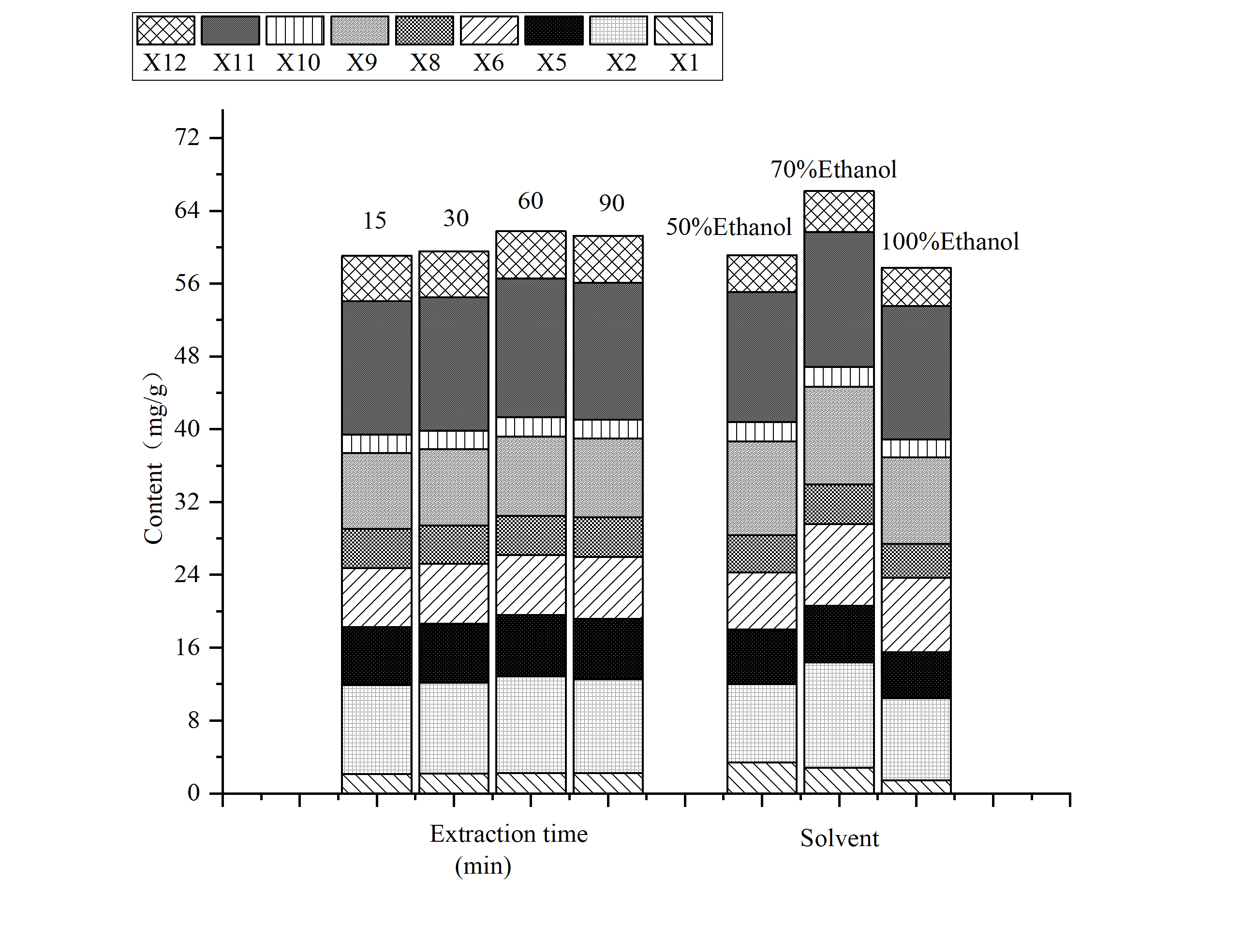


**Figure S6.** Effects of extraction solvent time and concentration on the extraction efficiency of the target analytes in CDBs, China. X12: pterostilbene; X11: loureirin B; X10: loureirin A; X9: 4H-1-benzopyran-4-one,2,3-dihydro-3,5,7-trihydroxy-3-[(4-methoxyphenyl)methyl]-,(R)-; X8: pinostilbene; X6: loureirin C; X5: loureirin D; X2:resveratrol; X1:p-hydroxybenzyl alcohol.


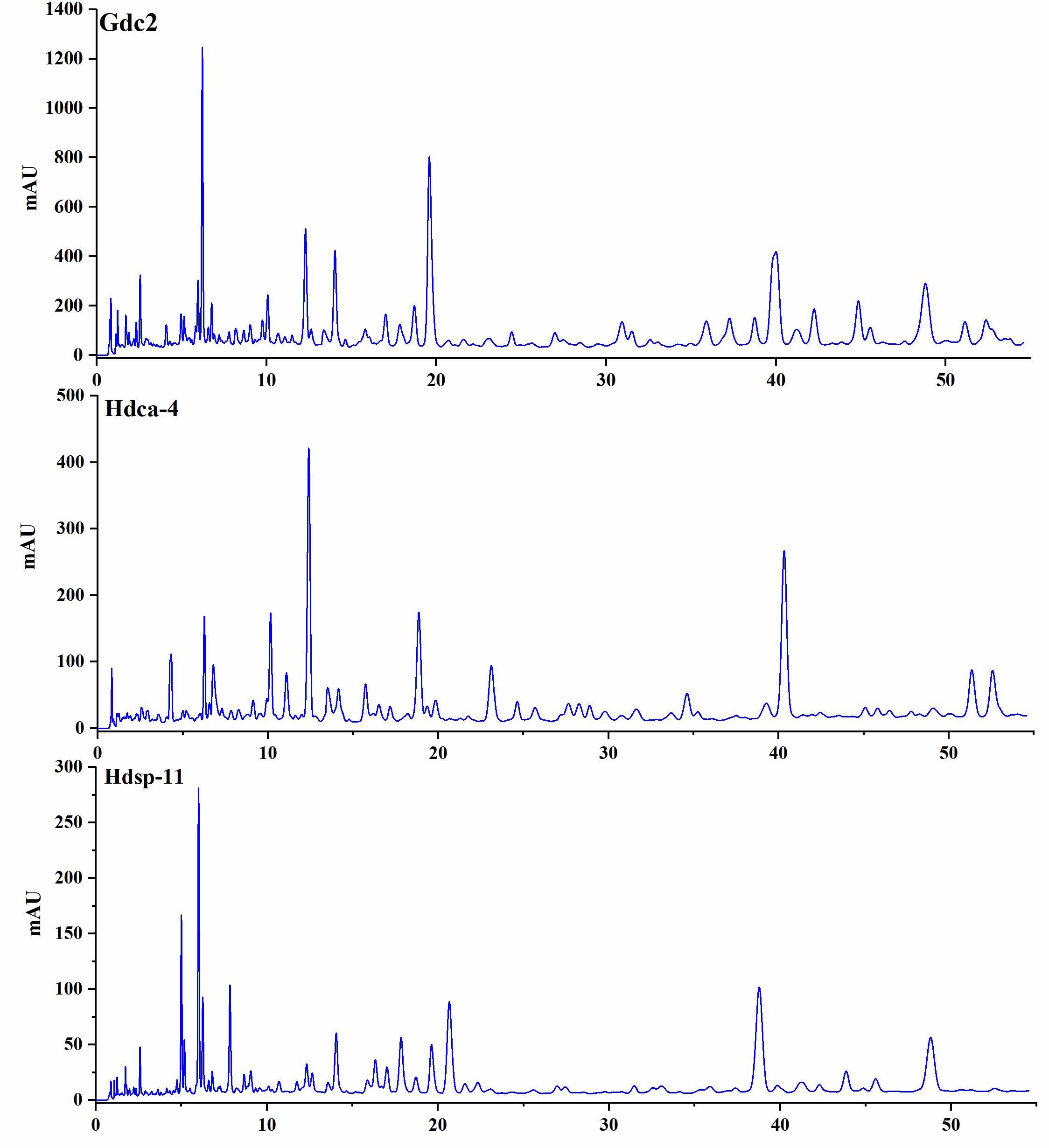


**Figure S7.** Representative Chromatogram under Optimization Conditions. UHPLC chromatograms of representative chromatograms of *D.cochinchinensis* (Gdc-2, Hdsp-11) and *D.cambodiana* (Hdca-4).

**Table S1** Geographical information on the origin of CDB samples

| Batch | Sample Code | City | variety | Latitude | Longitude | Tissue |
| --- | --- | --- | --- | --- | --- | --- |
| 1 | Gdc-1 | Baise | *D.cochinchinensis* | 23°5′33.80″N | 106°21′27.53″E | fat-containing wood |
| 2 | Gdc-2 | Baise |  | 23°5′31.32″N | 106°21′28.87″E |  |
| 3 | Gdc-3 | Baise |  | 23°5′33.66″N | 106°21′26.57″E |  |
| 4 | Gdc-4 | Baise |  | 23°5′33.66″N | 106°21′26.57″E |  |
| 5 | Gdc-5 | Baise |  | 23°5′33.66″N | 106°21′26.57″E |  |
| 6 | Gdc-6 | Baise |  | 23°5′33.66″N | 106°21′26.57″E |  |
| 7 | Gdc-7 | Chongzuo |  | 22°46′56.60″N | 107°8′37.62″E |  |
| 8 | Gdc-8 | Chongzuo |  | 22°46′55.27″N | 107°8′35.63″E |  |
| 9 | Gdc-9 | Chongzuo |  | 22°46′55.27″N | 107°8′35.63″E |  |
| 10 | Gdc-10 | Chongzuo |  | 22°46′54.15″N | 107°8′35.45″E |  |
| 11 | Gdc-11 | Chongzuo |  | 22°46′55.47″N | 107°8′37.44″E |  |
| 12 | Gdc-12 | Chongzuo |  | 22°46′55.47″N | 107°8′37.44″E |  |
| 13 | Gdc-13 | Chongzuo |  | 22°46′55.47″N | 107°8′37.44″E |  |
| 14 | Gdc-14 | Chongzuo |  | 22°46′55.47″N | 107°8′37.44″E |  |
| 15 | Gdc-15 | Chongzuo |  | 22°46′55.47″N | 107°8′37.44″E |  |
| 16 | Gdc-16 | Chongzuo |  | 22°27′24.78″N | 106°35′19.27″E |  |
| 17 | Gdc-17 | Chongzuo |  | 22°27′24.78″N | 106°35′19.27″E |  |
| 18 | Gdc-18 | Chongzuo |  | 22°27′24.78″N | 106°35′19.27″E |  |
| 19 | Gdc-19 | Chongzuo |  | 22°27′24.78″N | 106°35′19.27″E |  |
| 20 | Gdc-20 | Chongzuo |  | 22°27′24.78″N | 106°35′19.27″E |  |
| 21 | Gdc-21 | Chongzuo |  | 22°27′24.78″N | 106°35′19.27″E |  |
| 22 | Gdc-22 | Chongzuo |  | 22°27′24.78″N | 106°35′19.27″E |  |
| 23 | Gdc-23 | Chongzuo |  | 22°26′40.28″N | 106°37′8.44″E |  |
| 24 | Gdc-24 | Chongzuo |  | 22°23′21.56″N | 106°38′54.19″E |  |
| 25 | Gdc-25 | Chongzuo |  | 22°18′18.39″N | 106°58′3.47″E |  |
| 26 | Gdc-26 | Chongzuo |  | 22°18′18.39″N | 106°58′3.47″E |  |
| 27 | Gdc-27 | Chongzuo |  | 22°18′12.65″N | 106°58′17.54″E |  |
| 28 | Gdc-28 | Chongzuo |  | 22°18′12.65″N | 106°58′17.54″E |  |
| 29 | Gdc-29 | Chongzuo |  | 22°18′12.65″N | 106°58′17.54″E |  |
| 30 | Gdc-30 | Chongzuo |  | 22°18′12.65″N | 106°58′17.54″E |  |
| 31 | Gdc-31 | Chongzuo |  | 22°18′12.65″N | 106°58′17.54″E |  |
| 32 | Hdsp-1 | Dongfang |  | 18°51′56.96″N | 109°00′08.43″E |  |
| 33 | Hdsp-2 | Dongfang |  | 18°51′56.96″N | 109°00′08.43″E |  |
| 34 | Hdsp-3 | Dongfang |  | 18°51′56.96″N | 109°00′08.43″E |  |
| 35 | Hdsp-4 | Dongfang |  | 18°51′56.96″N | 109°00′08.43″E |  |
| 36 | Hdsp-5 | Dongfang |  | 18°51′56.96″N | 109°00′08.43″E |  |
| 37 | Hdsp-6 | Dongfang |  | 18°51′56.96″N | 109°00′08.43″E |  |
| 38 | Hdsp-7 | Dongfang |  | 18°51′56.96″N | 109°00′08.43″E |  |
| 39 | Hdsp-8 | Dongfang |  | 18°51′56.96″N | 109°00′08.43″E |  |
| 40 | Hdsp-9 | Dongfang |  | 18°51′56.96″N | 109°00′08.43″E |  |
| 41 | Hdsp-10 | Baoting |  | 18°35′56.01″N | 109°25′25.04″E |  |
| 42 | Hdsp-11 | Baoting |  | 18°35′56.16″N | 109°25′24.98″E |  |
| 43 | Hdsp-12 | Baoting |  | 18°35′56.16″N | 109°25′24.98″E |  |
| 44 | Hdca-1 | Wanning | *D.cambodiana* | 18°41'18.09"N | 110°13'43.27"E |  |
| 45 | Hdca-2 | Wanning |  | 18°35'18.25"N | 110°10'49.59"E |  |
| 46 | Hdca-3 | Sanya |  | 18°18'39.90"N | 109°9'31.47"E |  |
| 47 | Hdca-4 | Sanya |  | 18°14′15.97″N | 109°39′06.33″E |  |
| 48 | Hdca-5 | Sanya |  | 18°14′15.97″N | 109°39′06.33″E |  |
| 49 | Hdca-6 | lingshui |  | 18°29′58.87″N | 110°05′27.36″E |  |
| 50 | Hdca-7 | lingshui |  | 18°29′58.87″N | 110°05′27.36″E |  |
| 51 | Hdca-8 | Sanya |  | 18°14′15.97″N | 109°39′06.33″E |  |

**Table S2** 1237 metabolites annotated from three sets of CDB samples

| **NO.** | **chemical composition** | **MS2.score** | **level** | **rt** | **mz** | **type** | **Class** | **MEAN Gdc** | **MEAN Hdsp** | **MEAN Hdca** |
| --- | --- | --- | --- | --- | --- | --- | --- | --- | --- | --- |
| 1 | (-)-catechin | 1 | B(i) | 176.581 | 291.0832072 | POS | Flavonoids | 1.71 | 1.68 | 0.54 |
| 2 | 1-[2,4-dihydroxy-6-[(2s,3r,4s,5s,6r)-3,4,5-trihydroxy-6-(hydroxymethyl)oxan-2-yl]oxyphenyl]-3-(4-methoxyphenyl)propan-1-one | 1 | B(ii) | 216.091 | 451.1575975 | POS | Flavonoids | 0.01 | 0.00 | 0.03 |
| 3 | didymin | 1 | B(ii) | 534.484 | 595.1959238 | POS | Flavonoids | 0.28 | 0.36 | 0.02 |
| 4 | isosulochrin | 1 | B(ii) | 409.023 | 355.0930533 | POS | Aromatic polyketides | 0.64 | 0.30 | 0.95 |
| 5 | duartin (-) | 1 | B(ii) | 397.216 | 355.1147557 | POS | Isoflavonoids | 0.59 | 1.16 | 0.30 |
| 6 | pinoresinol dimethyl ether | 1 | B(ii) | 321.654 | 409.1611235 | POS | Lignans | 0.17 | 0.05 | 0.11 |
| 7 | catechin(+) | 1 | B(ii) | 227.531 | 291.0851182 | POS | Flavonoids | 0.01 | 0.01 | 0.01 |
| 8 | butyramide | 1 | B(ii) | 53.1681 | 88.07545895 | POS | Others | 0.11 | 0.10 | 0.13 |
| 9 | arbutin | 1 | B(ii) | 247.4505 | 295.0785954 | POS | Phenolic acids | 0.01 | 0.01 | 0.02 |
| 10 | 7-[(2s,3r,4s,5s,6r)-6-[[(2s,3r,4r)-3,4-dihydroxy-4-(hydroxymethyl)oxolan-2-yl]oxymethyl]-3,4,5-trihydroxyoxan-2-yl]oxy-5-hydroxy-3-(4-hydroxyphenyl)-6-methoxychromen-4-one | 1 | B(ii) | 492.39 | 595.1707816 | POS | Isoflavonoids | 0.36 | 0.45 | 0.11 |
| 11 | 3-hydroxybutyric acid | 1 | B(ii) | 780.377 | 103.0398556 | NEG | Fatty Acids and Conjugates | 0.17 | 0.17 | 0.17 |
| 12 | 3-methyl pyruvic acid | 1 | B(ii) | 779.46 | 101.0241953 | NEG | Fatty Acids and Conjugates | 0.01 | 0.01 | 0.01 |
| 13 | 5-hydroxymethyl-2-furancarboxylic acid | 1 | B(ii) | 795.636 | 141.0164976 | NEG | Cyclic polyketides | 0.46 | 0.45 | 0.41 |
| 14 | beta-hydroxymyristic acid | 1 | B(ii) | 514.614 | 243.1960267 | NEG | Fatty Acids and Conjugates | 0.03 | 0.03 | 0.01 |
| 15 | lactic acid | 1 | B(ii) | 781.103 | 89.02422517 | NEG | Fatty Acids and Conjugates | 0.01 | 0.01 | 0.01 |
| 16 | physalin b | 1 | B(ii) | 260.442 | 509.1794959 | NEG | Steroids | 0.00 | 0.00 | 0.00 |
| 17 | succinic acid | 0.999799308 | B(ii) | 127.7465 | 117.018944 | NEG | Fatty Acids and Conjugates | 0.79 | 0.64 | 0.49 |
| 18 | nonanoate | 0.999754 | B(ii) | 476.727 | 157.1230497 | NEG | Fatty Acids and Conjugates | 0.39 | 0.40 | 0.39 |
| 19 | alisol a | 0.999709462 | B(ii) | 694.131 | 489.357001 | NEG | Triterpenoids | 0.07 | 0.07 | 0.02 |
| 20 | kojic acid | 0.999667538 | B(ii) | 896.763 | 141.0164643 | NEG | Cyclic polyketides | 0.08 | 0.06 | 0.07 |
| 21 | adenosine | 0.999645 | B(i) | 207.346 | 268.1032119 | POS | Nucleosides | 0.18 | 0.23 | 0.11 |
| 22 | 1,9b-dihydroxy-6,6,9a-trimethyl-1,5,5a,7,8,9-hexahydrobenzo[e][2]benzofuran-3-one | 0.999481077 | B(ii) | 465.67 | 265.1475706 | NEG | Sesquiterpenoids | 0.21 | 0.14 | 0.16 |
| 23 | aesculin | 0.999437692 | B(ii) | 393.9495 | 341.0775368 | POS | Coumarins | 23.76 | 28.55 | 11.26 |
| 24 | 3,9-dimethoxy-6~{a},11~{a}-dihydro-6~{h}-[1]benzofuro[3,2-c]chromene | 0.999330462 | B(ii) | 452.158 | 285.1113156 | POS |  | 8.63 | 2.35 | 1.14 |
| 25 | 7-hydroxy-2-(4-hydroxyphenyl)-5-methoxy-2,3-dihydro-4h-chromen-4-one | 0.998804846 | B(ii) | 333.568 | 287.0900553 | POS | Flavonoids | 13.82 | 66.12 | 5.98 |
| 26 | mandelic acid, methyl ester | 0.998739538 | B(ii) | 783.317 | 165.0552468 | NEG | Others | 0.01 | 0.01 | 0.01 |
| 27 | pgd2 | 0.998739077 | B(ii) | 670.311 | 351.2201584 | NEG | Eicosanoids | 0.22 | 0.20 | 0.21 |
| 28 | betaine | 0.998448 | B(ii) | 44.8965 | 118.0858272 | POS | Others | 7.28 | 7.80 | 10.48 |
| 29 | coniferyl aldehyde | 0.997735154 | B(ii) | 325.772 | 177.0552551 | NEG | Phenylpropanoids | 0.02 | 0.01 | 0.06 |
| 30 | saponarin | 0.997562308 | B(i) | 334.23 | 595.1569009 | POS | Flavonoids | 0.33 | 1.51 | 0.10 |
| 31 | perseitol | 0.997451769 | B(ii) | 298.807 | 213.1018139 | POS | Fatty acyls | 0.01 | 0.01 | 0.03 |
| 32 | piceid | 0.997431385 | B(ii) | 282.0985 | 389.1240561 | NEG | Stilbenoids | 0.02 | 0.02 | 0.03 |
| 33 | (2s,3r,4r,5r,6s)-2-[(2r,3r,4s,5r,6r)-2-[2-(3,4-dihydroxyphenyl)ethoxy]-3,5-dihydroxy-6-(hydroxymethyl)oxan-4-yl]oxy-6-methyloxane-3,4,5-triol | 0.997340231 | B(ii) | 259.6445 | 461.1651331 | NEG | Phenylethanoids | 0.00 | 0.02 | 0.00 |
| 34 | sternbin | 0.997277769 | B(ii) | 316.692 | 301.0713113 | NEG | Flavonoids | 0.08 | 0.17 | 0.07 |
| 35 | 7-methoxyflavonol | 0.997123385 | B(ii) | 348.717 | 269.0798533 | POS | Flavonoids | 47.06 | 107.75 | 3.71 |
| 36 | kaempferol-3-o-glucoside-6''-p-coumaroyl | 0.996914077 | B(ii) | 361.5395 | 595.1571577 | POS | Flavonoids | 0.02 | 0.02 | 0.07 |
| 37 | 8-hydroxy-2-methoxy-6-methyl-1,4-naphthoquinone | 0.996723308 | B(ii) | 363.821 | 219.0646622 | POS | Naphthalenes | 0.01 | 0.22 | 0.01 |
| 38 | salicifolioside a | 0.996551615 | B(ii) | 393.317 | 627.2190719 | POS | Flavonoids | 0.50 | 0.85 | 0.18 |
| 39 | 9-(2,3-dihydroxy-3-methylbutyl)-4-methoxyfuro[3,2-g]chromen-7-one | 0.995885 | B(ii) | 293.2265 | 357.0729539 | POS | Coumarins | 8.35 | 10.19 | 7.00 |
| 40 | theanine | 0.995866231 | B(ii) | 430.312 | 192.1380021 | POS | Small peptides | 0.44 | 0.59 | 0.77 |
| 41 | acetosyringone | 0.995852231 | B(ii) | 321.169 | 195.0656297 | NEG |  | 0.02 | 0.03 | 0.02 |
| 42 | 2-hydroxyadipic acid | 0.995420538 | B(i) | 371.736 | 161.0453471 | NEG | Fatty Acids and Conjugates | 0.00 | 0.00 | 0.00 |
| 43 | 5,7-dihydroxy-4'-methoxyflavone | 0.995236231 | B(ii) | 497.627 | 283.0602892 | NEG | Flavonoids | 0.09 | 0.07 | 0.02 |
| 44 | eudesmin | 0.994812538 | B(ii) | 456.682 | 387.1789275 | POS | Lignans | 0.19 | 0.19 | 0.32 |
| 45 | l-glutamic acid | 0.994628462 | B(ii) | 40.9286 | 146.045391 | NEG | Small peptides | 0.01 | 0.02 | 0.02 |
| 46 | paeonoside | 0.994436923 | B(ii) | 264.066 | 327.1080884 | NEG | Flavonoids | 0.01 | 0.00 | 0.00 |
| 47 | trans-resveratrol | 0.994322154 | B(ii) | 347.343 | 227.0701027 | NEG | Stilbenoids | 27.92 | 30.65 | 0.16 |
| 48 | homogentisic acid | 0.994177462 | B(ii) | 249.718 | 167.0344804 | NEG | Phenolic acids | 0.05 | 0.04 | 0.05 |
| 49 | rhein | 0.994146615 | B(i) | 42.2792 | 283.0273799 | NEG | Polycyclic aromatic polyketides | 0.06 | 0.04 | 0.14 |
| 50 | 4'-hydroxy-5,7-dimethoxyflavanone | 0.993625615 | B(ii) | 359.263 | 301.1063063 | POS | Flavonoids | 4.16 | 11.46 | 5.78 |
| 51 | 11-oxoursolic acid acetate | 0.993579615 | B(ii) | 661.858 | 511.3407674 | NEG | Triterpenoids | 0.06 | 0.00 | 0.00 |
| 52 | (2r,3s,4s,5r,6s)-2-(hydroxymethyl)-6-[4-[(e)-3-hydroxyprop-1-enyl]phenoxy]oxane-3,4,5-triol | 0.992548462 | B(ii) | 321.877 | 311.1127669 | NEG | Phenylpropanoids | 2.23 | 1.22 | 1.27 |
| 53 | isoleucine | 0.992500692 | B(ii) | 47.24645 | 132.1015088 | POS | Small peptides | 0.08 | 0.13 | 0.09 |
| 54 | suberic acid | 0.992426154 | B(i) | 271.134 | 173.0814043 | NEG | Fatty Acids and Conjugates | 0.01 | 0.01 | 0.01 |
| 55 | 2',6'-dihydroxy-4'-methoxydihydrochalcone | 0.991000615 | B(ii) | 45.5769 | 271.1024768 | NEG | Flavonoids | 0.08 | 0.11 | 0.10 |
| 56 | vanillic acid | 0.990950692 | B(ii) | 250.604 | 169.0491657 | POS | Phenolic acids | 0.09 | 0.09 | 0.09 |
| 57 | 2',4'-dihydroxy-4-methoxychalcone | 0.990934308 | B(ii) | 374.335 | 271.095818 | POS | Flavonoids | 34.69 | 12.74 | 38.66 |
| 58 | 7-methoxy-3-(4-methoxyphenyl)-4h-chromen-4-one | 0.990681308 | B(ii) | 372.643 | 283.0957539 | POS | Isoflavonoids | 106.96 | 119.37 | 116.89 |
| 59 | cinnamate | 0.990103615 | B(ii) | 329.548 | 147.0447549 | NEG | Phenylpropanoids | 0.54 | 0.83 | 0.45 |
| 60 | azelaic acid | 0.990014923 | B(i) | 304.472 | 187.0972473 | NEG | Fatty Acids and Conjugates | 0.08 | 0.08 | 0.03 |
| 61 | sinapaldehyde | 0.989938077 | B(ii) | 338.161 | 207.0659902 | NEG | Phenylpropanoids | 0.26 | 0.11 | 0.13 |
| 62 | 5,7-dihydroxyflavanone | 0.989872692 | B(ii) | 359.927 | 255.0655231 | NEG | Flavonoids | 1.49 | 0.61 | 1.00 |
| 63 | (4s,5z,6s)-4-(2-methoxy-2-oxoethyl)-5-[2-[(e)-3-phenylprop-2-enoyl]oxyethylidene]-6-[(2s,3r,4s,5s,6r)-3,4,5-trihydroxy-6-(hydroxymethyl)oxan-2-yl]oxy-4h-pyran-3-carboxylic acid | 0.989777846 | B(ii) | 297.5435 | 207.0647508 | POS | Meroterpenoids | 0.06 | 0.11 | 0.51 |
| 64 | 9-hpode | 0.989284077 | B(ii) | 483.5175 | 311.2215809 | NEG | Octadecanoids | 0.05 | 0.03 | 0.01 |
| 65 | glyceraldehyde | 0.989281923 | B(ii) | 46.0977 | 89.02418395 | NEG | Saccharides | 0.31 | 0.23 | 0.23 |
| 66 | 6-methoxyflavone | 0.989252769 | B(ii) | 401.871 | 253.0849844 | POS | Flavonoids | 0.50 | 0.81 | 0.11 |
| 67 | methylmalonic acid | 0.988833077 | B(i) | 106.9625 | 117.0189416 | NEG | Fatty Acids and Conjugates | 0.90 | 0.71 | 0.67 |
| 68 | uric acid; lc-tdda; ce10 | 0.988792538 | B(ii) | 80.26635 | 167.0207812 | NEG |  | 0.06 | 0.03 | 0.00 |
| 69 | 9-hode | 0.988611923 | B(ii) | 549.657 | 295.2181708 | NEG | Octadecanoids | 0.01 | 0.00 | 0.00 |
| 70 | pinosylvin | 0.988495 | B(ii) | 425.866 | 211.0757001 | NEG | Stilbenoids | 2.62 | 4.13 | 0.00 |
| 71 | 3-(2-hydroxy-3,4-dimethoxyphenyl)-3,4-dihydro-2~{h}-chromen-7-ol | 0.988216538 | B(ii) | 433.791 | 303.1224015 | POS |  | 5.22 | 5.78 | 2.12 |
| 72 | 5-methoxysalicylic acid | 0.988015846 | B(ii) | 383.496 | 169.0491619 | POS | Phenolic acids | 0.10 | 0.03 | 0.02 |
| 73 | 3-(2-hydroxy-4-methoxyphenyl)-3,4-dihydro-2h-1-benzopyran-7-ol | 0.987298769 | B(ii) | 525.687 | 273.11127 | POS | Isoflavonoids | 0.53 | 0.22 | 0.06 |
| 74 | 5'-hydroxy-3'-methoxysativan | 0.986412923 | B(ii) | 397.858 | 333.1329264 | POS | Isoflavonoids | 3.87 | 8.54 | 0.62 |
| 75 | calycosin | 0.986212769 | B(ii) | 330.306 | 285.0750312 | POS | Isoflavonoids | 25.84 | 51.71 | 9.63 |
| 76 | madecassic acid | 0.985900154 | B(ii) | 688.465 | 503.3364123 | NEG | Triterpenoids | 0.02 | 0.03 | 0.04 |
| 77 | glycitin | 0.985798308 | B(i) | 274.579 | 447.1268791 | POS | Isoflavonoids | 0.01 | 0.07 | 0.01 |
| 78 | 2-methoxy-4-vinylphenol | 0.985266769 | B(ii) | 455.253 | 149.060489 | NEG | Phenylpropanoids | 0.21 | 0.12 | 0.44 |
| 79 | heneicosanoic acid (21:0) | 0.984233462 | B(ii) | 686.041 | 325.3107485 | NEG | Fatty Acids and Conjugates | 0.05 | 0.04 | 0.04 |
| 80 | licoflavone c | 0.983609 | B(ii) | 431.192 | 339.1220269 | POS | Flavonoids | 0.14 | 0.09 | 0.08 |
| 81 | hydroquinone | 0.983351538 | B(ii) | 519.075 | 111.0436933 | POS |  | 0.01 | 0.01 | 0.07 |
| 82 | (+)-dihydrowighteone | 0.982859308 | B(ii) | 492.827 | 341.1372686 | POS | Isoflavonoids | 0.04 | 0.20 | 0.03 |
| 83 | d-mannose | 0.982684308 | B(ii) | 328.7445 | 229.0170163 | NEG | Saccharides | 0.01 | 0.01 | 0.01 |
| 84 | 5,7-dihydroxy-2-(4-hydroxyphenyl)-6-[(2s,3r,4r,5s,6r)-3,4,5-trihydroxy-6-(hydroxymethyl)oxan-2-yl]-8-[(2s,3r,4r,5r,6s)-3,4,5-trihydroxy-6-methyloxan-2-yl]chromen-4-one | 0.982266615 | B(ii) | 513.858 | 579.1778197 | POS | Flavonoids | 0.81 | 0.34 | 0.13 |
| 85 | pinocembrin 7-o-benzoate | 0.980961538 | B(ii) | 403.672 | 361.1064825 | POS | Flavonoids | 0.31 | 0.12 | 0.11 |
| 86 | glutarate | 0.980494615 | B(ii) | 81.03745 | 131.0347071 | NEG | Fatty Acids and Conjugates | 0.01 | 0.01 | 0.01 |
| 87 | asparagine | 0.979768769 | B(ii) | 434.4315 | 133.0644365 | POS | Small peptides | 0.15 | 0.20 | 0.14 |
| 88 | 5-(1,2,4a,5-tetramethyl-7-oxo-3,4,8,8a-tetrahydro-2h-naphthalen-1-yl)-3-methylpentanoic acid | 0.979697308 | B(ii) | 587.795 | 319.2270084 | NEG | Diterpenoids | 0.00 | 0.01 | 0.00 |
| 89 | 2',4'-dihydroxychalcone | 0.979624538 | B(ii) | 484.8865 | 241.0850994 | POS | Flavonoids | 0.24 | 0.58 | 0.03 |
| 90 | [6]-gingerdiol 3,5-diacetate | 0.979476 | B(ii) | 326.9425 | 379.2121904 | NEG | Aromatic polyketides | 0.07 | 0.13 | 0.04 |
| 91 | butein | 0.978618462 | B(ii) | 382.008 | 271.0607913 | NEG | Flavonoids | 1.04 | 2.43 | 0.18 |
| 92 | 3,4,5-trihydroxystilbene; plasma id-333 | 0.976834308 | B(ii) | 412.016 | 227.0706784 | NEG | Stilbenoids | 0.05 | 0.07 | 0.00 |
| 93 | isoflavone base + 2o, o-hex | 0.976394154 | B(ii) | 283.411 | 417.1177177 | POS | Isoflavonoids | 0.05 | 0.03 | 0.02 |
| 94 | 3-(3,4,5-trimethoxyphenyl)propanoic acid | 0.976380846 | B(ii) | 388.605 | 241.1063631 | POS | Lignans | 0.23 | 0.38 | 0.03 |
| 95 | 4-methoxy-9,10-dihydrophenanthrene-2,7-diol | 0.976023615 | B(ii) | 408.739 | 241.0858484 | NEG | Phenanthrenoids | 29.01 | 16.40 | 0.76 |
| 96 | 2-methoxyresorcinol | 0.975954615 | B(ii) | 360.958 | 139.0401995 | NEG | Phenylpropanoids | 0.01 | 0.02 | 0.00 |
| 97 | 9-hydroxy-10,12-octadecadienoic acid | 0.975926615 | B(ii) | 536.254 | 295.2272515 | NEG | Octadecanoids | 0.64 | 0.57 | 0.18 |
| 98 | d-mannitol | 0.975877923 | B(ii) | 44.5377 | 181.0710743 | NEG | Fatty acyls | 1.06 | 1.79 | 1.56 |
| 99 | l-malic acid | 0.975698923 | B(ii) | 893.255 | 133.0139011 | NEG | Fatty Acids and Conjugates | 0.03 | 0.03 | 0.04 |
| 100 | 2,4-dihydroxyheptadec-16-ynyl acetate | 0.975679385 | B(ii) | 508.407 | 325.1830824 | NEG | Fatty acyls | 0.08 | 0.07 | 0.07 |
| 101 | 6,7-dimethoxy-4-methylcoumarin | 0.975436538 | B(ii) | 355.218 | 221.0804286 | POS | Coumarins | 0.08 | 0.11 | 0.85 |
| 102 | di(2-ethylhexyl)phthalate (dehp) | 0.974620923 | B(ii) | 721.719 | 391.2835197 | POS | Phenolic acids | 0.54 | 1.48 | 0.38 |
| 103 | 6-methoxy-7-hydroxycoumarin; plasma id-231 | 0.974562385 | B(ii) | 356.791 | 191.0346655 | NEG | Coumarins | 0.11 | 0.27 | 0.10 |
| 104 | malonic acid | 0.974355538 | B(ii) | 75.6154 | 103.0034721 | NEG | Fatty Acids and Conjugates | 0.01 | 0.01 | 0.01 |
| 105 | feruloyl tyramine | 0.973996923 | B(ii) | 352.412 | 314.1379748 | POS | Diarylheptanoids | 0.96 | 1.41 | 0.33 |
| 106 | 7-hydroxyflavone | 0.973982923 | B(ii) | 372.877 | 237.0557744 | NEG | Flavonoids | 0.00 | 0.00 | 0.00 |
| 107 | 4e,15z-bilirubin ixa | 0.973361769 | B(ii) | 584.112 | 585.2828637 | POS |  | 0.65 | 1.98 | 0.66 |
| 108 | 2-[[7-hydroxy-1-(4-hydroxy-3,5-dimethoxyphenyl)-3-(hydroxymethyl)-6,8-dimethoxy-1,2,3,4-tetrahydronaphthalen-2-yl]methoxy]-6-(hydroxymethyl)oxane-3,4,5-triol | 0.973253 | B(ii) | 260.798 | 600.2648926 | POS | Lignans | 0.11 | 0.02 | 0.01 |
| 109 | 7,4'-dihydroxyflavone | 0.972921231 | B(ii) | 343.414 | 253.04923 | NEG | Flavonoids | 25.79 | 15.08 | 7.79 |
| 110 | avenanthramide g | 0.972371 | B(ii) | 384.149 | 300.0941447 | POS |  | 1.59 | 1.83 | 0.75 |
| 111 | arginine | 0.971382769 | B(ii) | 44.1449 | 175.1184995 | POS | Small peptides | 0.44 | 0.42 | 0.76 |
| 112 | 2-[(3~{s},3~{a}~{r},5~{r},7~{a}~{s})-3~{a}-acetyl-3-hydroxy-7~{a}-methyl-2,3,4,5,6,7-hexahydro-1~{h}-inden-5-yl]prop-2-enoic acid | 0.970059077 | B(ii) | 311.007 | 289.1402795 | POS |  | 0.56 | 0.43 | 0.52 |
| 113 | glyflavanone b | 0.969800769 | B(ii) | 435.168 | 397.1643644 | POS | Flavonoids | 0.03 | 0.03 | 0.02 |
| 114 | xanthyletin | 0.968599538 | B(ii) | 390.0715 | 229.0851936 | POS | Coumarins | 0.35 | 0.21 | 0.06 |
| 115 | angolensin (r) | 0.968140769 | B(ii) | 388.555 | 271.0959718 | NEG | Flavonoids | 36.03 | 17.96 | 17.97 |
| 116 | [1-(3,5-dihydroxyphenyl)-12-hydroxytridecan-2-yl] acetate | 0.967964154 | B(ii) | 445.7015 | 365.2324657 | NEG | Others | 0.00 | 0.01 | 0.01 |
| 117 | lethedoside a | 0.967914385 | B(ii) | 481.704 | 491.1491101 | POS | Flavonoids | 1.66 | 4.39 | 0.33 |
| 118 | trans-pterostilbene | 0.966749308 | B(ii) | 480.311 | 255.1014073 | NEG | Stilbenoids | 104.00 | 43.58 | 1.43 |
| 119 | (16alpha,17beta)-estra-1,3,5(10)-triene-3,16,17-triol | 0.965084538 | B(ii) | 261.2535 | 311.1611619 | POS | Steroids | 0.01 | 0.01 | 0.04 |
| 120 | resveratrol 4'-methyl ether | 0.965041077 | B(ii) | 783.032 | 241.0867413 | NEG | Stilbenoids | 0.07 | 0.05 | 0.02 |
| 121 | sucrose | 0.964250615 | B(ii) | 47.5832 | 341.1084769 | NEG | Saccharides | 0.06 | 0.08 | 0.12 |
| 122 | arabitol | 0.964011154 | B(ii) | 45.0065 | 151.0606381 | NEG | Saccharides | 0.24 | 0.27 | 0.26 |
| 123 | aspartate | 0.963168923 | B(ii) | 40.2099 | 132.029992 | NEG | Small peptides | 0.01 | 0.01 | 0.01 |
| 124 | palmitic amide | 0.963045231 | B(ii) | 604.226 | 256.2626986 | POS | Others | 0.00 | 0.00 | 0.00 |
| 125 | fa 9:1+1o | 0.962585692 | B(ii) | 349.388 | 171.1021156 | NEG | Fatty Acids and Conjugates | 0.01 | 0.03 | 0.01 |
| 126 | 4-o-methylphloracetophenone | 0.962463308 | B(ii) | 404.5425 | 181.0501164 | NEG | Phloroglucinols | 0.03 | 0.02 | 0.07 |
| 127 | sn-glycero-3-phosphocholine | 0.962200769 | B(ii) | 42.9312 | 258.1096011 | POS | Others | 0.08 | 0.29 | 0.33 |
| 128 | cinnamyl benzoate | 0.961522769 | B(ii) | 465.669 | 239.1061678 | POS | Phenylpropanoids | 0.07 | 0.09 | 0.01 |
| 129 | curcumin | 0.961286923 | B(ii) | 457.862 | 367.1142861 | NEG | Diarylheptanoids | 0.02 | 0.01 | 0.00 |
| 130 | fallacinol (teloschistin) | 0.959449923 | B(ii) | 395.076 | 299.0555249 | NEG | Polycyclic aromatic polyketides | 0.94 | 1.41 | 0.37 |
| 131 | [3,4,5-trihydroxy-6-(hydroxymethyl)oxan-2-yl] 9-(hydroxymethyl)-2,2,6a,6b,9,12a-hexamethyl-10-[3,4,5-trihydroxy-6-[(3,4,5-trihydroxy-6-methyloxan-2-yl)oxymethyl]oxan-2-yl]oxy-1,3,4,5,6,6a,7,8,8a,10,11,12,13,14b-tetradecahydropicene-4a-carboxylate | 0.958155462 | B(ii) | 619.395 | 329.13786 | POS | Small peptides | 0.01 | 0.15 | 0.01 |
| 132 | 4-methoxyhomopterocarpin | 0.958043462 | B(ii) | 644.266 | 315.121558 | POS | Isoflavonoids | 0.02 | 0.26 | 0.01 |
| 133 | cinnamaldehyde | 0.957944 | B(ii) | 306.835 | 133.064428 | POS | Phenylpropanoids | 0.02 | 0.03 | 0.06 |
| 134 | 6-hydroxy-2-(4-methoxyphenyl)-4h-chromen-4-one | 0.957906692 | B(ii) | 409.514 | 269.0798817 | POS | Flavonoids | 6.14 | 10.70 | 0.67 |
| 135 | 4,6'-dihydroxy-2',3',4'-trimethoxychalcone | 0.9576 | B(ii) | 376.276 | 331.1165882 | POS | Flavonoids | 0.53 | 1.20 | 0.11 |
| 136 | 5,6,7-trimethoxycoumarin | 0.957118077 | B(ii) | 293.0005 | 235.0605636 | NEG | Coumarins | 0.00 | 0.00 | 0.01 |
| 137 | bungeiside c | 0.956832 | B(ii) | 553.6885 | 431.1480047 | POS | Others | 0.60 | 1.65 | 0.24 |
| 138 | 6-gingerol | 0.955876923 | B(ii) | 535.189 | 293.1749955 | NEG | Others | 0.00 | 0.00 | 0.00 |
| 139 | 10,16-dihydroxy-palmitic acid | 0.955838308 | B(ii) | 623.945 | 289.2361516 | POS | Fatty Acids and Conjugates | 0.02 | 0.02 | 0.03 |
| 140 | shanzhiside methyl ester | 0.955291231 | B(ii) | 341.084 | 429.1299939 | POS | Monoterpenoids | 3.74 | 1.72 | 0.11 |
| 141 | imperatorin | 0.954878846 | B(ii) | 374.074 | 309.0517464 | POS | Coumarins | 2.21 | 3.51 | 1.91 |
| 142 | feruloyl o-methyldopamine | 0.954537769 | B(ii) | 350.667 | 344.1486758 | POS | Diarylheptanoids | 2.23 | 3.32 | 0.03 |
| 143 | (2s)-4'-hydroxy-5,7,3'-trimethoxyflavan | 0.954464077 | B(ii) | 460.938 | 317.1378434 | POS | Flavonoids | 131.90 | 239.53 | 4.27 |
| 144 | (-)-mucronulatol | 0.953879077 | B(ii) | 419.6195 | 303.1223218 | POS | Isoflavonoids | 17.54 | 129.27 | 0.85 |
| 145 | phenylpyruvic acid | 0.953854385 | B(ii) | 297.625 | 163.0396176 | NEG | Phenolic acids | 0.32 | 0.21 | 0.27 |
| 146 | loganin | 0.953800462 | B(ii) | 291.302 | 389.1449828 | NEG | Monoterpenoids | 0.01 | 0.01 | 0.00 |
| 147 | fisetinidol | 0.952063846 | B(ii) | 322.2715 | 273.0760071 | NEG | Flavonoids | 0.16 | 0.28 | 0.09 |
| 148 | curcumenol | 0.951509923 | B(ii) | 555.1535 | 233.1543483 | NEG | Sesquiterpenoids | 0.08 | 0.09 | 0.07 |
| 149 | (s)-beta-aminoisobutyric acid | 0.951475308 | B(i) | 389.449 | 104.0702453 | POS |  | 0.25 | 0.20 | 0.23 |
| 150 | alpha,beta-dihydroresveratrol | 0.950714385 | B(ii) | 351.1245 | 229.0864972 | NEG | Stilbenoids | 0.14 | 0.12 | 0.00 |
| 151 | isorhapontin | 0.950469385 | B(ii) | 322.895 | 459.1054044 | POS | Stilbenoids | 0.04 | 0.07 | 0.13 |
| 152 | 4-hydroxy-3-methylbenzoic acid | 0.950113308 | B(ii) | 782.6355 | 151.039815 | NEG | Phenolic acids | 0.02 | 0.02 | 0.02 |
| 153 | guibourtinidol | 0.949815692 | B(ii) | 369.7555 | 259.095797 | POS | Flavonoids | 1.17 | 0.45 | 0.56 |
| 154 | 5,7-dimethoxy-2-phenyl-4h-chromen-4-one | 0.949773923 | B(ii) | 443.8445 | 283.0961206 | POS | Flavonoids | 0.93 | 2.08 | 0.90 |
| 155 | parasorbic acid | 0.947732846 | B(ii) | 72.9998 | 113.0593354 | POS | Macrolides | 0.51 | 0.52 | 0.56 |
| 156 | 6-methoxy-[2'',3'':7,8]furanoflavanone | 0.947619462 | B(ii) | 271.923 | 295.0936933 | POS | Flavonoids | 0.54 | 0.56 | 0.10 |
| 157 | 7,3'-dihydroxy-4'-methoxy-8-methylflavan | 0.946474154 | B(ii) | 575.508 | 287.1275165 | POS | Flavonoids | 1.84 | 0.94 | 0.31 |
| 158 | choline; ce10; oeyiohpdsnjkls-uhfffaoysa-n | 0.946360462 | B(ii) | 46.8206 | 104.1065422 | POS | Others | 9.98 | 11.75 | 5.82 |
| 159 | linoleoyl ethanolamide | 0.946039846 | B(ii) | 598.233 | 324.2891287 | POS | Fatty acyls | 0.47 | 0.37 | 0.10 |
| 160 | uridine-5-monophosphate | 0.945093692 | B(ii) | 786.2445 | 305.022015 | NEG | Nucleosides | 0.00 | 0.00 | 0.00 |
| 161 | methyl vanillate | 0.945003077 | B(ii) | 310.708 | 181.0501786 | NEG | Phenolic acids | 0.11 | 0.16 | 0.03 |
| 162 | yangonin | 0.944827154 | B(ii) | 303.702 | 259.095776 | POS | Styrylpyrones | 0.13 | 0.09 | 0.03 |
| 163 | ferulic acid | 0.943831462 | B(ii) | 360.391 | 193.050013 | NEG | Phenylpropanoids | 0.03 | 0.08 | 0.02 |
| 164 | 4-vinylphenol | 0.943378 | B(ii) | 433.027 | 119.0506503 | NEG | Phenylpropanoids | 0.06 | 0.05 | 0.03 |
| 165 | methysticin | 0.943212769 | B(ii) | 291.5255 | 275.0906921 | POS | Styrylpyrones | 0.03 | 0.06 | 0.09 |
| 166 | herniarin | 0.942870154 | B(ii) | 265.776 | 194.0807645 | POS | Coumarins | 0.05 | 0.06 | 0.01 |
| 167 | 3',4'-dihydroxy-5,7-dimethoxyflavan | 0.942077231 | B(ii) | 447.101 | 303.1224604 | POS | Flavonoids | 1.27 | 1.59 | 0.45 |
| 168 | 2-methyl-3-[(2s,3r,4s,5s,6r)-3,4,5-trihydroxy-6-(hydroxymethyl)oxan-2-yl]oxypyran-4-one | 0.941413692 | B(ii) | 388.6805 | 311.067479 | POS | Cyclic polyketides | 11.88 | 5.70 | 7.82 |
| 169 | diosgenin | 0.940467154 | B(ii) | 709.351 | 415.3198869 | POS | Steroids | 0.05 | 0.16 | 1.05 |
| 170 | dl-dopa | 0.940191769 | B(ii) | 760.3585 | 196.0622755 | NEG | Small peptides | 0.04 | 0.04 | 0.03 |
| 171 | rhapontigenin | 0.939939385 | B(ii) | 376.343 | 257.0814716 | NEG | Stilbenoids | 0.27 | 0.25 | 0.06 |
| 172 | alpinetin | 0.939409769 | B(ii) | 340.227 | 271.0955107 | POS | Flavonoids | 2.28 | 1.34 | 3.46 |
| 173 | 5,7-dihydroxy-3-[(4-methoxyphenyl)methyl]-2,3-dihydrochromen-4-one | 0.939012462 | B(ii) | 390.3945 | 301.1063752 | POS |  | 2.03 | 1.85 | 3.24 |
| 174 | [(2r,3s,4s,5r,6s)-6-[(2s,3r,4s,5r,6r)-6-[[(1s,3r,4s,4ar,8ar)-4-[(3s)-3-[(2s,3r,4r,5r,6r)-3,4-dihydroxy-6-methyl-5-[(2s,3r,4r,5r,6s)-3,4,5-trihydroxy-6-methyloxan-2-yl]oxyoxan-2-yl]oxy-3-methylpent-4-enyl]-3,4,8,8a-tetramethyl-1,2,3,4a,5,6-hexahydronaphthalen-1-yl]oxy]-4,5-dihydroxy-2-methyloxan-3-yl]oxy-3,4,5-trihydroxyoxan-2-yl]methyl acetate | 0.938622615 | B(ii) | 308.7815 | 947.4797955 | NEG | Diterpenoids | 0.07 | 0.01 | 0.01 |
| 175 | 5-hydroxyflavone | 0.938583308 | B(ii) | 255.1025 | 239.0672497 | POS | Flavonoids | 0.27 | 1.72 | 0.95 |
| 176 | (2e,4e,12z)-n-(2-methylpropyl)octadeca-2,4,12-trienamide | 0.938405385 | B(ii) | 592.894 | 334.3099368 | POS | Fatty acyls | 0.03 | 0.03 | 0.05 |
| 177 | genipin 1-beta-gentiobioside | 0.936955462 | B(ii) | 385.453 | 551.1874047 | POS | Monoterpenoids | 0.08 | 0.12 | 0.09 |
| 178 | 1-phenylethyl formate | 0.936456615 | B(ii) | 506.592 | 151.0750848 | POS |  | 0.93 | 0.37 | 0.06 |
| 179 | chlorogenate | 0.935696077 | B(ii) | 795.077 | 355.0939599 | POS | Phenylpropanoids | 0.17 | 0.28 | 0.12 |
| 180 | oxalic acid | 0.935670077 | B(i) | 393.742 | 88.98774184 | NEG | Fatty Acids and Conjugates | 0.03 | 0.03 | 0.01 |
| 181 | 4-hydroxybenzaldehyde | 0.935429308 | B(ii) | 10.8262 | 121.0292971 | NEG | Phenolic acids | 0.28 | 0.26 | 0.47 |
| 182 | isosativan | 0.935239615 | B(ii) | 518.486 | 287.1274866 | POS | Isoflavonoids | 6.16 | 3.67 | 0.21 |
| 183 | farrerol | 0.934645 | B(ii) | 477.361 | 299.0913354 | NEG | Flavonoids | 1.39 | 2.75 | 0.06 |
| 184 | n-carbamoyl-l-aspartate | 0.934505077 | B(i) | 369.471 | 175.0395886 | NEG |  | 0.00 | 0.00 | 0.01 |
| 185 | columbianetin | 0.932896308 | B(ii) | 367.589 | 245.0816483 | NEG | Coumarins | 0.03 | 0.07 | 0.02 |
| 186 | artoindonesianin a | 0.932869231 | B(ii) | 556.807 | 571.2684086 | POS | Flavonoids | 0.10 | 0.64 | 0.00 |
| 187 | riboflavin | 0.932833077 | B(ii) | 538.8965 | 375.1230039 | NEG | Anthranilic acid alkaloids | 0.03 | 0.02 | 0.00 |
| 188 | anabasamine | 0.931350077 | B(ii) | 200.227 | 254.1605332 | POS | Nicotinic acid alkaloids | 0.02 | 0.05 | 0.02 |
| 189 | isosafrole | 0.930606231 | B(ii) | 443.055 | 163.0749311 | POS | Phenylpropanoids | 0.38 | 0.11 | 0.24 |
| 190 | pyruvate | 0.930540538 | B(ii) | 778.582 | 87.00858768 | NEG | Fatty Acids and Conjugates | 0.04 | 0.04 | 0.04 |
| 191 | 3,5-dihydroxybenzoic acid | 0.930392692 | B(ii) | 385.156 | 155.0336034 | POS | Phenolic acids | 0.07 | 0.04 | 0.00 |
| 192 | medicarpin | 0.929848385 | B(ii) | 398.396 | 271.0956328 | POS | Isoflavonoids | 5.13 | 7.15 | 1.39 |
| 193 | monotesone a | 0.929771 | B(ii) | 336.927 | 357.1319688 | POS | Flavonoids | 0.23 | 0.26 | 0.63 |
| 194 | n6-(delta2-isopentenyl)-adenine | 0.929517385 | B(ii) | 48.8154 | 204.1227114 | POS | Pseudoalkaloids | 0.14 | 0.27 | 0.97 |
| 195 | isocrotonic acid | 0.928612 | B(ii) | 422.69 | 87.04385666 | POS | Fatty Acids and Conjugates | 0.11 | 0.11 | 0.11 |
| 196 | 5,6-dimethoxy-[2'',3'':7,8]furanoflavanone | 0.928289769 | B(ii) | 393.927 | 325.1036055 | POS | Flavonoids | 2.00 | 2.29 | 0.94 |
| 197 | 3,5,6,7,8,3',4'-heptamethoxyflavone | 0.926922077 | B(ii) | 288.774 | 433.1484629 | POS | Flavonoids | 0.08 | 0.03 | 0.03 |
| 198 | e-resveratrol trimethyl ether | 0.924786154 | B(ii) | 578.973 | 271.1317838 | POS | Stilbenoids | 0.48 | 0.16 | 0.05 |
| 199 | ginkgoic acid | 0.924535692 | B(ii) | 576.975 | 345.2429925 | NEG | Aromatic polyketides | 0.00 | 0.06 | 0.00 |
| 200 | oroxylin a | 0.924307692 | B(ii) | 451.242 | 283.0970753 | NEG | Flavonoids | 12.24 | 1.76 | 0.21 |
| 201 | sapidolide a | 0.924297154 | B(ii) | 303.971 | 267.1217204 | POS | Sesquiterpenoids | 1.18 | 0.84 | 0.56 |
| 202 | lophirone i | 0.923868154 | B(ii) | 413.451 | 373.1057962 | POS | Lignans | 0.42 | 0.33 | 0.11 |
| 203 | (r)-(e)-sulforaphene | 0.923764615 | B(ii) | 879.694 | 176.0227681 | POS | Others | 0.04 | 0.05 | 0.04 |
| 204 | otobanone | 0.923300923 | B(ii) | 460.8285 | 339.1194165 | POS | Lignans | 7.96 | 10.77 | 0.74 |
| 205 | threonic acid | 0.922021538 | B(ii) | 41.5319 | 135.0296507 | NEG | Fatty Acids and Conjugates | 0.03 | 0.02 | 0.01 |
| 206 | hydroxyphenyllactate | 0.921458462 | B(ii) | 365.7895 | 205.0466222 | POS |  | 0.08 | 0.04 | 0.10 |
| 207 | sinapoyl aldehyde | 0.920784846 | B(ii) | 392.055 | 209.0803082 | POS | Phenylpropanoids | 0.22 | 0.20 | 0.22 |
| 208 | alpha-glucose | 0.920491308 | B(ii) | 44.3379 | 179.0556226 | NEG | Saccharides | 0.17 | 0.12 | 0.15 |
| 209 | catechol | 0.919777692 | B(ii) | 477.066 | 111.043665 | POS |  | 0.03 | 0.02 | 0.05 |
| 210 | swertiamarin | 0.919639385 | B(ii) | 447.02 | 375.1215152 | POS | Monoterpenoids | 0.07 | 0.06 | 0.08 |
| 211 | 4'-hydroxy-7-methoxyflavan | 0.919327615 | B(ii) | 413.996 | 257.1168749 | POS | Flavonoids | 1.54 | 1.15 | 0.80 |
| 212 | hydroxyvalerenic acid | 0.919055231 | B(ii) | 498.816 | 289.1198613 | POS | Sesquiterpenoids | 0.09 | 0.33 | 0.21 |
| 213 | 5'-(furan-3-yl)-4a-hydroxy-4,7-dimethylspiro[5,6,7,8a-tetrahydro-1h-naphthalene-8,3'-oxolane]-2,2'-dione | 0.918017615 | B(ii) | 482.9305 | 331.1530933 | POS | Diterpenoids | 0.47 | 0.30 | 0.11 |
| 214 | decursinol angelate | 0.917781692 | B(ii) | 392.005 | 329.1381628 | POS | Coumarins | 0.18 | 0.29 | 0.13 |
| 215 | 5,10-dimethoxy-2,2-dimethylpyrano[3,2-g]chromen-8-one | 0.917493231 | B(ii) | 298.69 | 311.0885994 | POS | Coumarins | 0.02 | 0.02 | 0.03 |
| 216 | 3-hydroxycinnamic acid | 0.916932308 | B(i) | 271.084 | 209.0451819 | NEG | Phenylpropanoids | 0.02 | 0.02 | 0.02 |
| 217 | 5,7-dimethoxy-3-(4-methoxyphenyl)-4h-chromen-4-one | 0.916597 | B(ii) | 407.775 | 313.1064084 | POS | Isoflavonoids | 1.13 | 1.94 | 0.50 |
| 218 | candidone | 0.916509769 | B(ii) | 588.2 | 353.1742406 | POS | Flavonoids | 0.04 | 0.04 | 0.29 |
| 219 | lenticin | 0.916181 | B(ii) | 251.604 | 247.1438621 | POS | Others | 0.00 | 0.00 | 0.06 |
| 220 | 1-acetyl beta carboline | 0.915910538 | B(ii) | 454.131 | 211.0861171 | POS | Tryptophan alkaloids | 0.18 | 0.07 | 0.22 |
| 221 | 7-methoxy-2-methyl-3-phenyl-4h-chromen-4-one | 0.915131692 | B(ii) | 404.3905 | 267.1008481 | POS | Chromanes | 0.60 | 0.40 | 0.75 |
| 222 | triacetin (c2:0) | 0.914790308 | B(ii) | 339.543 | 236.1123578 | POS | Glycerolipids | 0.15 | 0.16 | 0.15 |
| 223 | picroside ii | 0.913923538 | B(ii) | 324.388 | 511.1602579 | NEG | Monoterpenoids | 0.23 | 0.19 | 0.02 |
| 224 | lotisoflavan | 0.913748462 | B(ii) | 523.122 | 303.1224134 | POS | Isoflavonoids | 0.74 | 2.04 | 0.07 |
| 225 | 3,7-dimethoxyflavone | 0.913727769 | B(ii) | 471.1415 | 283.0963004 | POS | Flavonoids | 0.52 | 0.92 | 0.49 |
| 226 | (4s,5z,6s)-5-[2-[(e)-3-(4-hydroxy-3-methoxyphenyl)prop-2-enoyl]oxyethylidene]-4-(2-methoxy-2-oxoethyl)-6-[(2s,3r,4s,5s,6r)-3,4,5-trihydroxy-6-(hydroxymethyl)oxan-2-yl]oxy-4h-pyran-3-carboxylic acid | 0.912960308 | B(ii) | 433.4005 | 597.1887002 | POS | Monoterpenoids | 7.74 | 4.64 | 6.95 |
| 227 | dihydropashanone | 0.911522846 | B(ii) | 272.566 | 303.1218234 | POS | Flavonoids | 0.02 | 0.01 | 0.27 |
| 228 | erysovine | 0.910993615 | B(ii) | 440.275 | 268.104412 | POS | Lysine alkaloids | 0.09 | 0.27 | 0.04 |
| 229 | citrinin | 0.910274615 | B(ii) | 283.3285 | 273.0754171 | POS | Chromanes | 0.16 | 0.08 | 0.01 |
| 230 | 6'-malonyl ononin | 0.910161692 | B(ii) | 40.9229 | 515.1239658 | NEG | Isoflavonoids | 0.00 | 0.00 | 0.00 |
| 231 | lapachol | 0.909710769 | B(ii) | 807.718 | 241.0868313 | NEG | Naphthalenes | 0.06 | 0.03 | 0.01 |
| 232 | epiafzelechin (2r,3r)(-) | 0.908283154 | B(ii) | 321.273 | 275.0904123 | POS | Flavonoids | 0.02 | 0.14 | 0.02 |
| 233 | 4'-hydroxyacetophenone 4'-[4-hydroxy-3,5-dimethoxybenzoyl-(->5)-apiosyl-(1->2)-glucoside] | 0.908039462 | B(ii) | 523.121 | 611.2034376 | POS |  | 4.83 | 13.37 | 0.09 |
| 234 | 9-oxoode | 0.907602077 | B(ii) | 572.825 | 293.2113875 | NEG | Octadecanoids | 0.04 | 0.04 | 0.03 |
| 235 | omega-hydroxydodecanoic acid | 0.907376846 | B(ii) | 438.972 | 215.1646792 | NEG | Fatty Acids and Conjugates | 0.03 | 0.04 | 0.03 |
| 236 | methyl haematommate | 0.906929077 | B(ii) | 232.048 | 209.0451404 | NEG | Aromatic polyketides | 0.10 | 0.09 | 0.09 |
| 237 | dl-liquiritigenin | 0.906719846 | B(ii) | 318.527 | 257.0799063 | POS | Flavonoids | 0.04 | 0.18 | 0.02 |
| 238 | endocrocin | 0.905992 | B(ii) | 282.417 | 313.0352412 | NEG | Polycyclic aromatic polyketides | 0.00 | 0.00 | 0.00 |
| 239 | bryacarpene 5 | 0.904981077 | B(ii) | 349.298 | 313.1063539 | POS | Isoflavonoids | 1.81 | 0.91 | 0.34 |
| 240 | 3,4-dihydroxybenzaldehyde | 0.904341692 | B(ii) | 257.362 | 137.0239375 | NEG | Phenolic acids | 0.33 | 0.28 | 0.14 |
| 241 | phyllodulcin | 0.904102462 | B(ii) | 393.949 | 287.0906662 | POS | Coumarins | 1.32 | 0.43 | 0.44 |
| 242 | 2-[2-(3,4-dimethoxyphenyl)ethyl]-4-methoxy-2,3-dihydropyran-6-one | 0.904068385 | B(ii) | 471.795 | 315.1218144 | POS | Styrylpyrones | 1.46 | 3.13 | 0.42 |
| 243 | methyl (1s,4as,7as)-4'-[(1s)-1-[(e)-3-(4-hydroxyphenyl)prop-2-enoyl]oxyethyl]-5'-oxo-1-[(2s,3r,4s,5s,6r)-3,4,5-trihydroxy-6-(hydroxymethyl)oxan-2-yl]oxyspiro[4a,7a-dihydro-1h-cyclopenta[c]pyran-7,2'-furan]-4-carboxylate | 0.902590923 | B(ii) | 513.8835 | 639.1643136 | POS | Monoterpenoids | 0.05 | 0.51 | 0.01 |
| 244 | 7-hydroxy-3-(4-methoxyphenyl)-4h-chromen-4-one | 0.902245615 | B(ii) | 533.4395 | 269.080019 | POS | Isoflavonoids | 1.06 | 0.91 | 0.96 |
| 245 | methyl 2-(2-methoxy-4-hydroxyphenyl)-6-methoxy-3-benzofurancarboxylate | 0.902237077 | B(ii) | 390.048 | 329.1014263 | POS | Isoflavonoids | 0.37 | 0.44 | 0.22 |
| 246 | 2',4'-dihydroxydihydrochalcone | 0.901626077 | B(ii) | 409.464 | 243.1009168 | POS | Flavonoids | 2.07 | 1.10 | 0.08 |
| 247 | coniferylaldehyde | 0.901328462 | B(ii) | 340.2525 | 179.0697514 | POS | Phenylpropanoids | 0.16 | 0.09 | 0.09 |
| 248 | hastatoside | 0.901189615 | B(ii) | 456.737 | 405.1321909 | POS | Monoterpenoids | 0.06 | 0.68 | 0.17 |
| 249 | triptonide | 0.900437769 | B(ii) | 443.8295 | 359.1480701 | POS | Diterpenoids | 0.03 | 0.08 | 0.03 |
| 250 | reynosin | 0.900182923 | B(ii) | 519.797 | 271.1319373 | POS | Sesquiterpenoids | 4.85 | 6.47 | 0.15 |
| 251 | benzoylformate | 0.899846154 | B(ii) | 276.399 | 149.0239519 | NEG | Flavonoids | 0.01 | 0.01 | 0.00 |
| 252 | l-carnitine | 0.899307538 | B(ii) | 45.0105 | 162.1120227 | POS |  | 2.78 | 2.31 | 2.28 |
| 253 | isomaltose | 0.898476 | B(i) | 486.89 | 401.1389884 | NEG | Saccharides | 0.28 | 0.11 | 0.03 |
| 254 | wogonin | 0.898449846 | B(ii) | 350.6605 | 283.0602004 | NEG | Flavonoids | 3.63 | 1.82 | 0.51 |
| 255 | feruloyltyramine | 0.896928923 | B(ii) | 351.911 | 312.1231862 | NEG | Diarylheptanoids | 0.07 | 0.11 | 0.02 |
| 256 | aschantin | 0.896524154 | B(ii) | 308.185 | 401.1569636 | POS | Lignans | 1.86 | 1.10 | 0.04 |
| 257 | conessine | 0.896199077 | B(ii) | 675.542 | 355.3206259 | NEG | Pseudoalkaloids | 0.10 | 0.13 | 0.14 |
| 258 | (2r,3s,4s,5r,6r)-2-(hydroxymethyl)-6-[(e)-3-(4-hydroxyphenyl)prop-2-enoxy]oxane-3,4,5-triol | 0.896041385 | B(ii) | 289.4085 | 351.0836004 | POS | Phenylpropanoids | 0.15 | 0.07 | 0.07 |
| 259 | icariside i | 0.895174923 | B(ii) | 495.5865 | 531.1774964 | POS | Flavonoids | 0.00 | 0.02 | 0.21 |
| 260 | guanine; ce30; uytpupdqbnuygx-uhfffaoysa-n | 0.895165077 | B(ii) | 88.20265 | 152.0565249 | POS | Histidine alkaloids | 0.17 | 0.04 | 0.01 |
| 261 | 5-[6-(3-hydroxy-4-methoxyphenyl)-1,3,3a,4,6,6a-hexahydrofuro[3,4-c]furan-3-yl]-2-methoxyphenol | 0.895047769 | B(ii) | 443.8445 | 397.1042804 | POS | Others | 0.08 | 0.33 | 0.02 |
| 262 | (-)-perillyl alcohol | 0.894913846 | B(ii) | 288.5545 | 135.1166825 | POS |  | 0.06 | 0.02 | 0.00 |
| 263 | (2s,3r,4s,5s,6r)-2-[[(1s,4ar,7as)-7-(hydroxymethyl)-1,4a,5,7a-tetrahydrocyclopenta[c]pyran-1-yl]oxy]-6-(hydroxymethyl)oxane-3,4,5-triol | 0.894145077 | B(ii) | 265.279 | 329.1241072 | NEG | Monoterpenoids | 0.01 | 0.01 | 0.00 |
| 264 | 2'-hydroxy-3',4',6'-trimethoxydihydrochalcone | 0.892909 | B(ii) | 520.449 | 317.1378993 | POS | Flavonoids | 3.95 | 8.23 | 0.24 |
| 265 | (2s,3r)-3-hydroxy-4-methoxy-2-(2-phenylethyl)-2,3-dihydropyran-6-one | 0.892871923 | B(ii) | 386.1165 | 271.095612 | POS | Styrylpyrones | 18.63 | 4.96 | 13.08 |
| 266 | 6-pentadecyl salicylic acid | 0.892845385 | B(ii) | 600.147 | 347.2589605 | NEG | Aromatic polyketides | 0.00 | 0.01 | 0.00 |
| 267 | licochalcone b | 0.892100462 | B(ii) | 371.1035 | 287.0907974 | POS | Flavonoids | 0.41 | 0.47 | 0.55 |
| 268 | haematoxylin | 0.891579 | B(ii) | 358.7255 | 301.0710583 | NEG |  | 0.41 | 2.09 | 0.41 |
| 269 | citrusin | 0.890765385 | B(ii) | 310.605 | 349.1270835 | POS | Phenylpropanoids | 0.02 | 0.03 | 0.18 |
| 270 | 6,4'-dimethoxyisoflavone-7-glucoside | 0.890112923 | B(ii) | 46.9238 | 459.1351435 | NEG | Isoflavonoids | 0.00 | 0.01 | 0.00 |
| 271 | phenylethyl primeveroside | 0.889984462 | B(ii) | 543.7 | 417.1695065 | POS | Phenylethanoids | 0.51 | 0.16 | 0.01 |
| 272 | [2-(hydroxymethyl)-5,5,8a-trimethyl-1,4,4a,6,7,8-hexahydronaphthalen-1-yl]methanol | 0.889447308 | B(ii) | 571.744 | 237.1853442 | NEG | Sesquiterpenoids | 0.02 | 0.00 | 0.00 |
| 273 | 4-methoxy-9-(2-methylbut-3-en-2-yl)furo[3,2-g]chromen-7-one | 0.887269308 | B(ii) | 364.485 | 323.0678036 | POS | Coumarins | 0.84 | 0.38 | 1.50 |
| 274 | indole | 0.887128231 | B(ii) | 453.437 | 118.0647912 | POS | Tryptophan alkaloids | 0.08 | 0.06 | 0.03 |
| 275 | methyl 3-(3-methylbut-2-enyl)-1,4-bis[[3,4,5-trihydroxy-6-(hydroxymethyl)oxan-2-yl]oxy]naphthalene-2-carboxylate | 0.886685538 | B(ii) | 512.603 | 649.1830464 | POS | Naphthalenes | 0.02 | 0.03 | 0.22 |
| 276 | 9-oxootre | 0.886415615 | B(ii) | 403.131 | 293.2100834 | POS | Octadecanoids | 0.09 | 0.04 | 0.11 |
| 277 | p-anisic acid | 0.885500846 | B(ii) | 243.014 | 151.0397988 | NEG | Phenolic acids | 0.07 | 0.11 | 0.04 |
| 278 | (2z)-6-hydroxy-2-[(4-hydroxy-3-methoxyphenyl)methylidene]-1-benzofuran-3-one | 0.885347308 | B(ii) | 462.5905 | 285.0751821 | POS | Flavonoids | 1.57 | 1.71 | 0.47 |
| 279 | lusianthridin | 0.884091923 | B(ii) | 276.4305 | 241.071058 | NEG | Phenanthrenoids | 0.03 | 0.00 | 0.00 |
| 280 | apigeniflavan | 0.883038462 | B(ii) | 411.6805 | 259.0959063 | POS | Flavonoids | 0.39 | 0.44 | 0.16 |
| 281 | vinetorin | 0.882623923 | B(ii) | 523.765 | 305.0217297 | NEG | Xanthones | 0.00 | 0.00 | 0.04 |
| 282 | pomiferin | 0.881890692 | B(ii) | 779.353 | 419.1491674 | NEG | Isoflavonoids | 0.00 | 0.00 | 0.01 |
| 283 | atractylenolide iii | 0.880087538 | B(ii) | 499.4025 | 271.1319656 | POS | Sesquiterpenoids | 1.95 | 1.39 | 0.06 |
| 284 | benzaldehyde | 0.879964308 | B(ii) | 435.121 | 107.0488258 | POS | Phenolic acids | 0.30 | 0.28 | 0.35 |
| 285 | abruquinone b | 0.879861769 | B(ii) | 588.2 | 391.1304142 | POS |  | 0.07 | 0.05 | 0.63 |
| 286 | retusin 7-methyl ether | 0.879713077 | B(ii) | 522.05 | 299.0905018 | POS | Isoflavonoids | 0.67 | 1.05 | 1.63 |
| 287 | isokobusone | 0.878842 | B(ii) | 552.583 | 221.1540487 | NEG | Sesquiterpenoids | 0.04 | 0.04 | 0.03 |
| 288 | catalposide | 0.878657923 | B(ii) | 373.249 | 483.1429928 | POS | Monoterpenoids | 0.48 | 0.38 | 0.01 |
| 289 | phloridzin | 0.878314 | B(ii) | 284.09 | 435.1289483 | NEG | Flavonoids | 0.00 | 0.00 | 0.00 |
| 290 | afzelechin | 0.877121769 | B(ii) | 290.3345 | 273.0760257 | NEG | Flavonoids | 0.01 | 0.01 | 0.01 |
| 291 | oleuropein | 0.877105385 | B(ii) | 485.54 | 541.1848856 | POS | Monoterpenoids | 0.69 | 1.25 | 0.45 |
| 292 | p-hydroxyphenethyl trans-ferulate | 0.877051846 | B(ii) | 455.506 | 315.121645 | POS | Phenylethanoids | 0.79 | 2.20 | 0.35 |
| 293 | glyinflanin h | 0.876826154 | B(ii) | 200.3455 | 309.1171723 | POS | Isoflavonoids | 0.01 | 0.01 | 0.16 |
| 294 | 3-hydroxy-1-(4-hydroxyphenyl)propan-1-one | 0.875576923 | B(ii) | 258.873 | 165.0552071 | NEG |  | 0.04 | 0.04 | 0.04 |
| 295 | naringenin | 0.874803615 | B(ii) | 252.018 | 273.0729178 | POS | Flavonoids | 0.04 | 0.04 | 0.04 |
| 296 | (x)-p-menth-1-en-4-yl 5-isopropyl-2-methylphenyl ether | 0.873567615 | B(ii) | 661.4085 | 287.2365926 | POS | Monoterpenoids | 0.05 | 0.01 | 0.01 |
| 297 | flavone base + 2o, 2meo, c-hex | 0.873278462 | B(ii) | 779.5685 | 477.1332683 | POS | Flavonoids | 0.03 | 0.07 | 0.01 |
| 298 | pinolenic acid | 0.871810692 | B(ii) | 421.652 | 279.2314164 | POS | Fatty Acids and Conjugates | 0.09 | 0.07 | 0.12 |
| 299 | indolepropionic acid | 0.871447538 | B(ii) | 245.231 | 190.0857568 | POS | Tryptophan alkaloids | 0.10 | 0.11 | 0.28 |
| 300 | 6-o-methylnorlaudanosoline | 0.871407846 | B(ii) | 512.559 | 302.1458833 | POS |  | 0.46 | 0.76 | 0.04 |
| 301 | homovanillate | 0.870587769 | B(ii) | 324.4425 | 181.0504104 | NEG | Phenolic acids | 0.08 | 0.04 | 0.02 |
| 302 | amorphigenin | 0.870209077 | B(ii) | 362.536 | 433.1273362 | POS | Isoflavonoids | 0.20 | 0.46 | 0.32 |
| 303 | 2-phenylethyl b-d-glucopyranoside | 0.869662154 | B(ii) | 310.8 | 307.1167946 | POS | Phenylethanoids | 0.01 | 0.01 | 0.03 |
| 304 | s(8-8)s hexoside | 0.868449077 | B(ii) | 272.715 | 579.2087365 | NEG | Lignans | 0.00 | 0.00 | 0.00 |
| 305 | santonin | 0.868021692 | B(ii) | 439.704 | 269.1167487 | POS | Sesquiterpenoids | 0.33 | 0.34 | 0.61 |
| 306 | 5,7-dihydroxy-2-phenyl-6-[3,4,5-trihydroxy-6-(hydroxymethyl)oxan-2-yl]-8-(3,4,5-trihydroxyoxan-2-yl)chromen-4-one | 0.867677 | B(ii) | 468.543 | 549.1527395 | POS | Flavonoids | 0.06 | 0.26 | 0.02 |
| 307 | 3,4-dimethoxydalbergione | 0.867465077 | B(ii) | 540.201 | 285.1111041 | POS | Flavonoids | 0.13 | 0.09 | 0.15 |
| 308 | oppositin | 0.867093923 | B(ii) | 397.142 | 421.1273814 | POS | Flavonoids | 0.02 | 0.43 | 0.10 |
| 309 | gibberellic acid | 0.866388846 | B(ii) | 468.5375 | 347.1485207 | POS | Diterpenoids | 0.19 | 0.50 | 0.01 |
| 310 | (+)-8-hydroxy-5,5-dimethylpeltogynan | 0.865868462 | B(ii) | 409.6005 | 331.1167352 | POS | Flavonoids | 0.23 | 0.30 | 0.16 |
| 311 | hydrocortisone | 0.864685846 | B(ii) | 385.664 | 361.2014716 | NEG | Steroids | 0.03 | 0.28 | 0.23 |
| 312 | 3,5-dimethoxyphenol | 0.864602538 | B(ii) | 419.471 | 155.0698597 | POS | Phloroglucinols | 0.96 | 6.98 | 0.03 |
| 313 | 3-methoxybenzaldehyde | 0.864340692 | B(ii) | 619.979 | 137.0595164 | POS | Phenolic acids | 0.47 | 0.25 | 0.04 |
| 314 | daidzin | 0.863668077 | B(i) | 74.1494 | 415.1090269 | NEG | Isoflavonoids | 0.01 | 0.01 | 0.00 |
| 315 | 4-(2,3-dihydroxy-3-methylbutoxy)furo(3,2-g)chromen-7-one | 0.863075462 | B(ii) | 197.381 | 305.0991712 | POS | Coumarins | 0.22 | 0.37 | 0.04 |
| 316 | linolenic acid | 0.862576077 | B(ii) | 618.108 | 277.2159618 | NEG | Fatty Acids and Conjugates | 1.02 | 0.80 | 0.52 |
| 317 | 7-[(2e,5e)-7-hydroxy-3,7-dimethylocta-2,5-dienoxy]chromen-2-one | 0.862293692 | B(ii) | 516.5405 | 313.1436186 | NEG | Coumarins | 0.06 | 0.01 | 0.30 |
| 318 | obtusaquinone | 0.861332923 | B(ii) | 505.457 | 253.0865015 | NEG | Flavonoids | 0.02 | 0.02 | 0.00 |
| 319 | 4'-hydroxy-5,7-dimethoxyflavan | 0.859322 | B(ii) | 434.501 | 287.1274306 | POS | Flavonoids | 4.66 | 6.79 | 3.79 |
| 320 | arachidic acid | 0.859295308 | B(ii) | 733.818 | 311.294979 | NEG | Fatty Acids and Conjugates | 0.00 | 0.01 | 0.01 |
| 321 | 5,7-dihydroxy-2-methyl-8-[(2~{s},3~{r},4~{r},5~{s},6~{r})-3,4,5-trihydroxy-6-(hydroxymethyl)oxan-2-yl]chromen-4-one | 0.857456846 | B(ii) | 460.737 | 355.0929013 | POS |  | 96.78 | 135.87 | 9.81 |
| 322 | 2-[[7-hydroxy-1-(4-hydroxy-3,5-dimethoxyphenyl)-3-(hydroxymethyl)-6,8-dimethoxy-1,2,3,4-tetrahydronaphthalen-2-yl]methoxy]oxane-3,4,5-triol | 0.856931385 | B(ii) | 306.365 | 570.252779 | POS | Lignans | 0.06 | 0.08 | 0.01 |
| 323 | (2r,3r)-3,7-dihydroxy-6-methoxy-2-phenyl-2,3-dihydrochromen-4-one | 0.855536923 | B(ii) | 356.487 | 285.0763516 | NEG | Flavonoids | 1.99 | 2.00 | 1.50 |
| 324 | plumieride | 0.855271 | B(ii) | 515.919 | 469.1419117 | NEG | Monoterpenoids | 0.06 | 0.01 | 0.22 |
| 325 | 4-hydroxyphenylacetaldehyde | 0.854639923 | B(ii) | 443.9825 | 137.0594094 | POS |  | 8.91 | 3.21 | 5.39 |
| 326 | loganic acid | 0.854555462 | B(i) | 376.204 | 375.1228021 | NEG | Monoterpenoids | 0.36 | 0.28 | 0.00 |
| 327 | 12-opda | 0.853788 | B(ii) | 448.899 | 293.2103503 | POS | Octadecanoids | 0.06 | 0.05 | 0.05 |
| 328 | capensine | 0.853412385 | B(ii) | 275.1485 | 275.091721 | NEG | Coumarins | 0.03 | 0.05 | 0.06 |
| 329 | 3',4',5',7,8-pentamethoxyflavan | 0.853290615 | B(ii) | 529.679 | 361.1641564 | POS | Flavonoids | 0.02 | 0.17 | 0.00 |
| 330 | methylpyrazine | 0.852487077 | B(ii) | 765.947 | 95.06012431 | POS | Peptide alkaloids | 0.11 | 0.12 | 0.10 |
| 331 | proline betaine; ce10; cmunutvvoohqpw-lurjtmiesa-n | 0.851866462 | B(ii) | 48.8154 | 144.1016807 | POS | Others | 2.43 | 2.38 | 3.36 |
| 332 | goniothalenol | 0.851848385 | B(ii) | 213.262 | 233.078359 | POS | Styrylpyrones | 0.48 | 0.41 | 0.45 |
| 333 | syringic acid | 0.851160231 | B(ii) | 209.1335 | 197.0450527 | NEG | Phenolic acids | 0.00 | 0.00 | 0.00 |
| 334 | pterocarpan base + 1o, 1meo | 0.850956308 | B(ii) | 327.6795 | 269.0811369 | NEG | Isoflavonoids | 2.01 | 1.25 | 2.75 |
| 335 | 3-methoxyapigenin | 0.850427231 | B(ii) | 312.163 | 301.0701377 | POS |  | 0.07 | 0.39 | 0.18 |
| 336 | perilloside e | 0.850248 | B(ii) | 445.05 | 371.1272682 | POS | Phenylpropanoids | 0.03 | 0.09 | 0.01 |
| 337 | (r)-(+)-1,2-dithiolane-3-pentanoic acid | 0.850192385 | B(ii) | 784.837 | 223.0195377 | NEG | Fatty Acids and Conjugates | 0.02 | 0.02 | 0.01 |
| 338 | sarisan | 0.848033154 | B(ii) | 433.229 | 193.0856148 | POS | Phenylpropanoids | 0.09 | 0.09 | 0.06 |
| 339 | 5,7-dimethoxy-2h-chromen-2-one | 0.847474538 | B(ii) | 437.47 | 207.0647638 | POS | Coumarins | 0.06 | 0.11 | 0.23 |
| 340 | alizarin | 0.847015692 | B(ii) | 801.42 | 241.0488063 | POS | Polycyclic aromatic polyketides | 0.54 | 0.52 | 0.55 |
| 341 | cephaeline | 0.846168615 | B(ii) | 533.685 | 489.2833116 | POS | Tyrosine alkaloids | 0.07 | 0.09 | 0.19 |
| 342 | 1-acetoxy-4,6-tetradecadiene-8,10,12-triyne | 0.846075769 | B(ii) | 563.187 | 241.1220335 | POS | Fatty acyls | 0.12 | 0.12 | 0.00 |
| 343 | strictosamide | 0.845528385 | B(ii) | 334.275 | 543.1983915 | NEG | Tryptophan alkaloids | 0.05 | 0.05 | 0.02 |
| 344 | (2r,3s,4s,5r,6s)-2-(hydroxymethyl)-6-[4-prop-2-enyl-2-[(2s,3r,4s,5s,6r)-3,4,5-trihydroxy-6-(hydroxymethyl)oxan-2-yl]oxyphenoxy]oxane-3,4,5-triol | 0.845253077 | B(ii) | 234.667 | 492.205988 | POS | Phenylpropanoids | 0.02 | 0.02 | 0.01 |
| 345 | osthol | 0.843923462 | B(ii) | 308.589 | 245.116671 | POS | Coumarins | 0.11 | 0.06 | 0.17 |
| 346 | 3'-methyl-2',4',6'-trihydroxydihydrochalcone | 0.843271385 | B(ii) | 511.9425 | 273.1113235 | POS | Flavonoids | 0.77 | 0.41 | 0.11 |
| 347 | isotectorigenin, 7-methyl ether | 0.843137692 | B(ii) | 401.891 | 327.0866193 | NEG | Isoflavonoids | 0.03 | 0.04 | 0.02 |
| 348 | 2-[[3,4-dihydroxy-4-(hydroxymethyl)oxolan-2-yl]oxymethyl]-6-[(5-hydroxy-1,7,7-trimethyl-2-bicyclo[2.2.1]heptanyl)oxy]oxane-3,4,5-triol | 0.842469385 | B(ii) | 291.5845 | 482.2593991 | POS |  | 0.07 | 0.02 | 0.00 |
| 349 | fraxin | 0.842386692 | B(ii) | 479.017 | 371.0891058 | POS | Coumarins | 0.18 | 0.20 | 0.00 |
| 350 | 3,3',4,5'-tetrahydroxy-trans-stilbene | 0.841999308 | B(ii) | 319.191 | 243.0660055 | NEG | Stilbenoids | 0.14 | 0.73 | 0.01 |
| 351 | maltose | 0.841824615 | B(ii) | 46.64 | 360.1490736 | POS | Saccharides | 2.65 | 4.53 | 4.78 |
| 352 | glyceric acid | 0.838997154 | B(ii) | 58.7318 | 105.0191114 | NEG | Fatty Acids and Conjugates | 0.01 | 0.01 | 0.01 |
| 353 | elemicin | 0.838700385 | B(ii) | 268.449 | 209.1164828 | POS | Phenylpropanoids | 0.13 | 0.10 | 0.02 |
| 354 | cardamonin | 0.837774923 | B(ii) | 485.879 | 269.0812664 | NEG | Flavonoids | 0.49 | 1.01 | 0.08 |
| 355 | hecogenin | 0.835690615 | B(ii) | 559.48 | 429.3007725 | NEG | Steroids | 0.01 | 0.01 | 0.01 |
| 356 | digitoxin | 0.835106 | B(ii) | 603.07 | 823.4482303 | NEG | Steroids | 0.00 | 0.00 | 0.01 |
| 357 | ureidosuccinic acid | 0.833626231 | B(i) | 339.429 | 175.0396132 | NEG |  | 0.01 | 0.00 | 0.03 |
| 358 | 2',4',6'-trihydroxy-4-methoxydihydrochalcone | 0.833571538 | B(ii) | 435.151 | 289.1064037 | POS | Flavonoids | 1.48 | 1.54 | 0.84 |
| 359 | baccatin iii | 0.832120846 | B(ii) | 354.2375 | 625.2044236 | POS | Diterpenoids | 0.60 | 0.23 | 0.13 |
| 360 | 7-oxocholesterol | 0.831619077 | B(ii) | 734.131 | 401.3401425 | POS | Steroids | 0.68 | 1.17 | 1.88 |
| 361 | angoroside a | 0.831221846 | B(ii) | 403.779 | 757.2629332 | POS | Phenylethanoids | 0.16 | 0.38 | 0.02 |
| 362 | 11-eicosenoic acid | 0.830377231 | B(ii) | 722.755 | 309.2792171 | NEG | Fatty Acids and Conjugates | 0.46 | 0.44 | 0.53 |
| 363 | (2r,3s,4s,5r,6s)-2-(hydroxymethyl)-6-(4-hydroxy-2-methyl-5-propan-2-ylphenoxy)oxane-3,4,5-triol | 0.828786846 | B(ii) | 636.596 | 327.1491216 | NEG | Monoterpenoids | 0.01 | 0.01 | 0.01 |
| 364 | eugenitin | 0.828509 | B(ii) | 388.713 | 221.0800023 | POS | Chromanes | 0.05 | 0.08 | 1.12 |
| 365 | l-phenylalanine | 0.828406308 | B(i) | 188.769 | 166.0861365 | POS | Small peptides | 0.03 | 0.05 | 0.03 |
| 366 | fargesin | 0.827925077 | B(ii) | 316.3055 | 371.1465632 | POS | Lignans | 1.09 | 2.38 | 0.07 |
| 367 | 1-oleoyl-rac-glycerol | 0.826071538 | B(ii) | 596.787 | 357.2998112 | POS | Glycerolipids | 0.07 | 0.11 | 0.03 |
| 368 | hesperidin methyl chalcone | 0.825496615 | B(ii) | 272.895 | 625.2125635 | POS | Flavonoids | 0.00 | 0.03 | 0.00 |
| 369 | 4-o-caffeoyl-3-o-feruloylquinic acid | 0.824603 | B(ii) | 492.195 | 531.1407836 | POS | Phenylpropanoids | 0.02 | 0.18 | 0.01 |
| 370 | daidzein | 0.824053923 | B(ii) | 783.8725 | 255.0647743 | POS | Isoflavonoids | 0.18 | 0.13 | 0.13 |
| 371 | naringin | 0.823755308 | B(ii) | 540.0135 | 603.1774621 | POS | Flavonoids | 0.00 | 0.00 | 0.12 |
| 372 | betulinic acid | 0.82328 | B(ii) | 696.8165 | 457.3664886 | POS | Triterpenoids | 0.12 | 0.15 | 0.13 |
| 373 | fragransol b | 0.822478615 | B(ii) | 529.566 | 331.153102 | POS | Lignans | 0.15 | 0.65 | 0.06 |
| 374 | p-anisaldehyde | 0.822108846 | B(ii) | 492.843 | 137.0595552 | POS | Phenolic acids | 9.06 | 6.69 | 0.29 |
| 375 | (e)-3-(2-hydroxyphenyl)-2-propenal | 0.822012538 | B(ii) | 360.933 | 149.0592178 | POS | Phenylpropanoids | 0.23 | 1.01 | 0.05 |
| 376 | 2-methoxy-4-hydroxydihydrochalcone | 0.821707308 | B(ii) | 395.668 | 257.1167116 | POS | Flavonoids | 1.80 | 1.81 | 0.66 |
| 377 | 7,4'-dihydroxy-3',5'-dimethoxyisoflavanone | 0.821405308 | B(ii) | 250.775 | 317.1018512 | POS | Isoflavonoids | 0.05 | 0.06 | 0.07 |
| 378 | mesquitol-4alpha-ol 8-methyl ether | 0.820149154 | B(ii) | 252.3985 | 321.0967066 | POS | Flavonoids | 0.03 | 0.05 | 0.03 |
| 379 | osthenol | 0.819717692 | B(ii) | 270.125 | 231.099033 | POS | Coumarins | 0.20 | 0.22 | 0.20 |
| 380 | 5,3'-dihydroxy-2,4-dimethoxydalbergiquinol | 0.818507846 | B(ii) | 420.617 | 287.1275918 | POS |  | 1.77 | 4.21 | 0.58 |
| 381 | dioscin | 0.818366769 | B(ii) | 519.1975 | 867.472473 | NEG | Steroids | 0.07 | 0.04 | 0.49 |
| 382 | (~{e})-3-phenyl-~{n}-(2-phenylethyl)prop-2-enamide | 0.818187231 | B(ii) | 786.001 | 252.1433303 | POS |  | 0.03 | 0.03 | 0.03 |
| 383 | sinapyl alcohol | 0.818106538 | B(ii) | 364.63 | 209.0814283 | NEG | Phenylpropanoids | 0.05 | 0.01 | 0.01 |
| 384 | uncinatone | 0.816729692 | B(ii) | 285.5305 | 327.1566948 | POS | Diterpenoids | 0.05 | 0.01 | 0.00 |
| 385 | harmine | 0.816420846 | B(i) | 44.6896 | 257.08754 | NEG | Tryptophan alkaloids | 0.02 | 0.03 | 0.02 |
| 386 | rescinnamine | 0.816320692 | B(ii) | 465.188 | 673.2428336 | POS | Tryptophan alkaloids | 0.35 | 0.63 | 0.10 |
| 387 | benzoic acid + 1o, 1meo, o-hex | 0.815482846 | B(ii) | 207.573 | 329.0845379 | NEG | Phenolic acids | 0.01 | 0.01 | 0.01 |
| 388 | epimedoside a | 0.814349231 | B(ii) | 329.126 | 685.1916421 | POS | Flavonoids | 0.02 | 0.01 | 0.02 |
| 389 | (-)-gallocatechin | 0.812506462 | B(ii) | 360.321 | 287.0911585 | NEG | Flavonoids | 5.83 | 19.70 | 1.65 |
| 390 | maltitol | 0.812172385 | B(ii) | 46.8174 | 343.1240437 | NEG | Saccharides | 0.02 | 0.04 | 0.01 |
| 391 | 5-hydroxy-3-(4-hydroxybenzyl)-7,8-dimethoxy-4-chromanone | 0.811117 | B(ii) | 293.3 | 331.1165448 | POS | Isoflavonoids | 0.90 | 1.30 | 0.43 |
| 392 | (2r,3r)-3,5-dihydroxy-2-(4-hydroxyphenyl)-7-methoxy-2,3-dihydrochromen-4-one | 0.810715923 | B(ii) | 380.201 | 303.0858394 | POS | Flavonoids | 1.28 | 2.88 | 0.46 |
| 393 | (2~{s},3~{r},4~{s},5~{s},6~{r})-2-[4-[(~{e})-2-(3-hydroxy-5-methoxyphenyl)ethenyl]phenoxy]-6-(hydroxymethyl)oxane-3,4,5-triol | 0.810161308 | B(ii) | 335.5425 | 403.1384616 | NEG |  | 0.11 | 0.08 | 0.08 |
| 394 | zeaxanthin | 0.809725923 | B(ii) | 730.95 | 568.4261681 | POS | Carotenoids (C40) | 0.16 | 0.09 | 1.75 |
| 395 | 13z-docosenamide | 0.809274692 | B(ii) | 641.631 | 338.3407598 | POS | Fatty Acids and Conjugates | 0.03 | 0.03 | 0.03 |
| 396 | ruscogenin | 0.809018231 | B(ii) | 487.4205 | 431.3150113 | POS | Steroids | 0.40 | 0.08 | 0.18 |
| 397 | methyl levulinate | 0.808961308 | B(ii) | 45.99665 | 131.0698909 | POS | Fatty esters | 0.06 | 0.06 | 0.05 |
| 398 | pinocembrine | 0.808893231 | B(ii) | 309.8005 | 255.0656233 | NEG | Flavonoids | 0.01 | 0.02 | 0.01 |
| 399 | 7-hydroxy-5,4'-dimethoxyflavan | 0.808640846 | B(ii) | 783.4715 | 287.1273438 | POS | Flavonoids | 0.49 | 0.29 | 0.16 |
| 400 | oxyresveratrol | 0.807814769 | B(ii) | 369.051 | 245.0797237 | POS | Stilbenoids | 0.03 | 0.03 | 0.02 |
| 401 | 4-methylvaleric acid | 0.807592385 | B(ii) | 357.019 | 115.0760853 | NEG | Fatty Acids and Conjugates | 0.01 | 0.01 | 0.01 |
| 402 | 2-linoleoyl glycerol | 0.807171077 | B(ii) | 560.1235 | 355.2835682 | POS | Glycerolipids | 0.03 | 0.03 | 0.02 |
| 403 | barbaloin | 0.806149077 | B(i) | 48.3008 | 417.1243348 | NEG |  | 0.02 | 0.01 | 0.02 |
| 404 | methyl orsellinate | 0.806136538 | B(ii) | 383.3425 | 181.0499927 | NEG | Aromatic polyketides | 0.64 | 0.36 | 0.03 |
| 405 | anisocoumarin h | 0.805923615 | B(ii) | 457.3295 | 332.1856221 | POS | Coumarins | 0.05 | 0.02 | 0.01 |
| 406 | (~{e})-1-(1,3,6,8-tetramethoxynaphthalen-2-yl)but-2-en-1-one | 0.805286769 | B(ii) | 782.7985 | 317.1374675 | POS |  | 0.41 | 0.64 | 0.27 |
| 407 | quebrachitol | 0.803925154 | B(ii) | 49.1983 | 233.0419344 | POS | Polyols | 0.31 | 0.11 | 0.16 |
| 408 | calcitriol | 0.803421231 | B(ii) | 704.4935 | 399.325404 | POS | Steroids | 0.24 | 0.70 | 0.36 |
| 409 | salsoline | 0.802469154 | B(ii) | 214.91 | 194.1173134 | POS | Tyrosine alkaloids | 0.06 | 0.05 | 0.04 |
| 410 | (r)-shinanolone | 0.800632615 | B(ii) | 359.037 | 193.0855531 | POS | Naphthalenes | 0.25 | 0.57 | 0.08 |
| 411 | (2~{s},3~{r},5~{r},10~{r},13~{r},14~{s},17~{s})-2,3,14-trihydroxy-10,13-dimethyl-17-[(2~{r},3~{r})-2,3,6-trihydroxy-6-methylheptan-2-yl]-2,3,4,5,9,11,12,15,16,17-decahydro-1~{h}-cyclopenta[a]phenanthren-6-one | 0.800391385 | B(ii) | 341.557 | 427.2829756 | POS |  | 0.19 | 0.18 | 0.05 |
| 412 | (2~{s},3~{r},4~{r},5~{r},6~{r})-2-methyl-6-[[(2~{r},3~{s},4~{s},5~{r},6~{r})-3,4,5-trihydroxy-6-(2-phenylethoxy)oxan-2-yl]methoxy]oxane-3,4,5-triol | 0.798672154 | B(iii) | 287.931 | 448.2167931 | POS |  | 0.07 | 0.04 | 0.29 |
| 413 | 2',4'-dihydroxyacetophenone | 0.798543308 | B(iii) | 401.645 | 151.0397656 | NEG | Others | 0.08 | 0.07 | 0.02 |
| 414 | ponganone xi | 0.798116846 | B(iii) | 268.65 | 293.0777705 | POS | Flavonoids | 0.12 | 0.16 | 0.02 |
| 415 | tyrosine | 0.797759846 | B(iii) | 84.2926 | 182.0806678 | POS | Small peptides | 0.02 | 0.03 | 0.01 |
| 416 | [(1~{s},3~{r},8~{r},10~{s},11~{r},12~{s},14~{s},16~{r})-12-acetyloxy-5,11,15,15-tetramethyl-6-oxo-2,7-dioxapentacyclo[8.8.0.0^{1,3}.0^{4,8}.0^{11,16}]octadec-4-en-14-yl] acetate | 0.797341077 | B(iii) | 490.779 | 415.2102583 | POS |  | 0.19 | 0.19 | 0.59 |
| 417 | 1-naphthol | 0.795397615 | B(iii) | 369.624 | 145.0645465 | POS | Naphthalenes | 0.14 | 0.03 | 0.04 |
| 418 | [(1as,1bs,2s,5ar,6s,6as)-1a-(hydroxymethyl)-2-[(2s,3r,4s,5s,6r)-3,4,5-trihydroxy-6-(hydroxymethyl)oxan-2-yl]oxy-2,5a,6,6a-tetrahydro-1bh-oxireno[5,6]cyclopenta[1,3-c]pyran-6-yl] (e)-3-(3-hydroxy-4-methoxyphenyl)prop-2-enoate | 0.794690692 | B(iii) | 563.85 | 537.1538372 | NEG | Monoterpenoids | 0.02 | 0.05 | 0.01 |
| 419 | cirsimaritin | 0.794192769 | B(iii) | 314.696 | 313.0706686 | NEG | Flavonoids | 0.02 | 0.03 | 0.21 |
| 420 | tomatidine | 0.794142308 | B(iii) | 738.832 | 416.3599849 | POS | Pseudoalkaloids | 0.21 | 0.21 | 0.08 |
| 421 | arctigenin | 0.793028692 | B(iii) | 398.494 | 395.1478737 | POS | Lignans | 2.59 | 1.72 | 1.88 |
| 422 | mexicanolide | 0.792955769 | B(iii) | 522.227 | 507.1795447 | POS | Triterpenoids | 1.42 | 0.53 | 1.02 |
| 423 | osajin | 0.791473308 | B(iii) | 560.592 | 403.1548833 | NEG | Isoflavonoids | 0.03 | 0.01 | 0.00 |
| 424 | phenylacetic acid | 0.790340077 | B(iii) | 427.796 | 135.0447329 | NEG | Phenolic acids | 0.49 | 0.18 | 0.61 |
| 425 | glycitein | 0.78947 | B(iii) | 349.938 | 285.0750557 | POS | Isoflavonoids | 20.83 | 5.35 | 1.21 |
| 426 | [3,4-dihydroxy-4-(7-methoxy-2-oxochromen-8-yl)-2-methylidenebutyl] 3-methylbutanoate | 0.789028 | B(iii) | 333.667 | 399.140801 | POS |  | 0.22 | 0.27 | 0.07 |
| 427 | (3~{r})-5-methyl-3-[(2~{s},3~{r},4~{s},5~{s},6~{r})-3,4,5-trihydroxy-6-(hydroxymethyl)oxan-2-yl]oxy-3-[[4-[(2~{s},3~{r},4~{s},5~{s},6~{r})-3,4,5-trihydroxy-6-(hydroxymethyl)oxan-2-yl]oxyphenyl]methoxycarbonyl]hexanoic acid | 0.788787231 | B(iii) | 308.147 | 619.224061 | NEG |  | 0.02 | 0.02 | 0.02 |
| 428 | 2e,4z-heptadienal | 0.787776846 | B(iii) | 375.6305 | 111.0801086 | POS | Fatty acyls | 0.02 | 0.02 | 0.01 |
| 429 | 4-coumaryl alcohol | 0.787497538 | B(iii) | 289.948 | 151.0750529 | POS | Phenylpropanoids | 0.04 | 0.03 | 0.02 |
| 430 | 7-hydroxy-3-(3-hydroxy-4-methoxybenzyl)-5-methoxy-4-chromanone | 0.787275692 | B(iii) | 333.564 | 331.1166336 | POS | Isoflavonoids | 0.05 | 0.05 | 0.17 |
| 431 | demethylvestitol | 0.785334231 | B(iii) | 348.608 | 259.095985 | POS | Isoflavonoids | 0.89 | 1.17 | 0.17 |
| 432 | alnustin | 0.784459538 | B(iii) | 364.661 | 329.1017746 | POS | Flavonoids | 0.20 | 0.48 | 0.12 |
| 433 | secoisolariciresinol | 0.784418 | B(iii) | 334.246 | 361.1644887 | NEG | Lignans | 0.72 | 0.32 | 0.38 |
| 434 | vicenin iii | 0.783956077 | B(iii) | 418.3075 | 565.1458259 | POS | Flavonoids | 0.25 | 0.35 | 0.26 |
| 435 | 3,4-dimethoxycinnamic acid | 0.783769154 | B(iii) | 378.908 | 207.0657407 | NEG | Phenylpropanoids | 0.04 | 0.04 | 0.06 |
| 436 | 2,4,6-trimethoxyphenyl acetate | 0.781921077 | B(iii) | 377.618 | 227.0909715 | POS | Phloroglucinols | 0.49 | 0.09 | 0.54 |
| 437 | eudesmic acid | 0.781901769 | B(iii) | 266.16 | 211.0609157 | NEG | Phenolic acids | 0.01 | 0.03 | 0.01 |
| 438 | 24-epi-brassinolide | 0.781685385 | B(iii) | 694.061 | 479.3284639 | NEG | Steroids | 0.02 | 0.03 | 0.01 |
| 439 | epishyobunone | 0.781328692 | B(iii) | 379.805 | 221.1894245 | POS | Sesquiterpenoids | 0.12 | 0.03 | 0.01 |
| 440 | rosmadial | 0.780572231 | B(iii) | 334.862 | 345.1685396 | POS | Diterpenoids | 0.15 | 0.07 | 0.08 |
| 441 | 17-[2,3-dihydroxy-6-methyl-6-[3,4,5-trihydroxy-6-(hydroxymethyl)oxan-2-yl]oxyheptan-2-yl]-2,3,14-trihydroxy-10,13-dimethyl-2,3,4,5,9,11,12,15,16,17-decahydro-1~{h}-cyclopenta[a]phenanthren-6-one | 0.779789231 | B(iii) | 303.824 | 625.3583292 | POS |  | 0.97 | 0.83 | 0.02 |
| 442 | isosakuranin | 0.779658308 | B(iii) | 280.1595 | 447.1281513 | NEG | Flavonoids | 0.01 | 0.00 | 0.00 |
| 443 | paeonilactone b | 0.779499769 | B(iii) | 298.101 | 195.0656147 | NEG | Sesquiterpenoids | 0.11 | 0.03 | 0.03 |
| 444 | isotaxiresinol | 0.778630462 | B(iii) | 486.567 | 345.1338835 | NEG | Lignans | 0.03 | 0.05 | 0.01 |
| 445 | (1~{r},2~{s},5~{s},8~{r})-7,7-dimethyl-6-methylidenetricyclo[6.2.1.0^{1,5}]undecane-2-carboxylic acid | 0.777982 | B(iii) | 505.273 | 217.15842 | POS |  | 0.02 | 0.01 | 0.07 |
| 446 | n-methyl-2-pyrrolidone | 0.777445692 | B(iii) | 215.987 | 100.0753993 | POS | Ornithine alkaloids | 1.22 | 1.09 | 1.09 |
| 447 | 3-hydroxy-3-methylglutaric acid | 0.776915615 | B(iii) | 83.7411 | 161.0453064 | NEG | Fatty Acids and Conjugates | 0.01 | 0.01 | 0.01 |
| 448 | dehydrorotenone | 0.776072385 | B(iii) | 333.591 | 415.1151746 | POS | Isoflavonoids | 1.56 | 2.06 | 0.29 |
| 449 | crotonoside | 0.774972692 | B(iii) | 444.035 | 284.0995188 | POS | Nucleosides | 0.11 | 0.32 | 0.14 |
| 450 | benzophenone | 0.773826615 | B(iii) | 495.4395 | 183.0798961 | POS | Flavonoids | 0.03 | 0.02 | 0.03 |
| 451 | 1,7-dimethyl-7-(4-methylpent-3-enyl)bicyclo[2.2.1]heptan-2-ol | 0.773554308 | B(iii) | 612.758 | 264.2316338 | POS |  | 0.05 | 0.02 | 0.04 |
| 452 | ligustilide | 0.773373308 | B(iii) | 425.97 | 213.0906268 | POS | Cyclic polyketides | 0.07 | 0.09 | 0.01 |
| 453 | uridine | 0.772868923 | B(iii) | 319.411 | 279.0423473 | NEG | Nucleosides | 0.01 | 0.03 | 0.00 |
| 454 | aucubin | 0.772584 | B(i) | 257.578 | 405.138496 | NEG | Monoterpenoids | 0.15 | 0.07 | 0.03 |
| 455 | benzyl butyl phthalate | 0.772379308 | B(iii) | 490.246 | 313.1430395 | POS | Phenolic acids | 0.05 | 0.07 | 0.10 |
| 456 | methyl 3-ethenyl-4-(2-oxoethyl)-2-[3,4,5-trihydroxy-6-(hydroxymethyl)oxan-2-yl]oxy-3,4-dihydro-2~{h}-pyran-5-carboxylate | 0.771867077 | B(iii) | 395.9185 | 389.1370299 | POS |  | 0.16 | 0.39 | 0.53 |
| 457 | methyl 3,4,5-trimethoxycinnamate | 0.771849231 | B(iii) | 262.871 | 275.0903086 | POS | Phenylpropanoids | 0.06 | 0.14 | 0.10 |
| 458 | gibberellin a87 | 0.771185923 | B(iii) | 262.728 | 363.1428214 | POS | Diterpenoids | 0.01 | 0.01 | 0.02 |
| 459 | (9a-hydroxy-3,8a-dimethyl-5-methylidene-2-oxo-4,4a,6,7,8,9-hexahydrobenzo[f][1]benzofuran-8-yl) acetate | 0.770900615 | B(iii) | 498.249 | 329.1380553 | POS | Sesquiterpenoids | 0.13 | 0.38 | 0.05 |
| 460 | (2s)-2-(3,4-dihydroxyphenyl)-5,7-dihydroxy-2,3-dihydro-4h-chromen-4-one | 0.770899231 | B(iii) | 132.806 | 311.0523553 | POS | Flavonoids | 0.12 | 0.13 | 0.07 |
| 461 | protodioscin | 0.769769846 | B(iii) | 342.108 | 1047.534117 | NEG | Steroids | 0.00 | 0.00 | 0.04 |
| 462 | violaxanthin | 0.768993692 | B(iii) | 658.694 | 600.4163836 | POS | Carotenoids (C40) | 0.11 | 0.05 | 0.08 |
| 463 | lactucin | 0.768668769 | B(iii) | 409.6275 | 277.1063301 | POS | Sesquiterpenoids | 0.14 | 0.12 | 0.06 |
| 464 | umbelliferone | 0.768532077 | B(iii) | 800.721 | 185.0207243 | POS | Coumarins | 0.71 | 0.72 | 0.70 |
| 465 | 5,7-dihydroxy-8-(3-methylbut-2-en-1-yl)-2-phenylchroman-4-one | 0.767877231 | B(iii) | 268.9005 | 325.1393751 | POS |  | 0.04 | 0.04 | 0.03 |
| 466 | 2-pyrrolidinone; aif; ce10; ms2dec | 0.766968308 | B(iii) | 211.922 | 86.05984558 | POS | Others | 0.11 | 0.13 | 0.11 |
| 467 | p-methoxycinnamic acid ethyl ester | 0.766760154 | B(iii) | 308.601 | 207.100796 | POS | Phenylpropanoids | 0.24 | 0.14 | 0.04 |
| 468 | 7-hydroxy-5,8,2'-trimethoxyflavanone | 0.766044154 | B(iii) | 449.522 | 331.1167285 | POS | Flavonoids | 0.12 | 0.26 | 0.30 |
| 469 | calycosin-7-o-beta-d-glucoside | 0.765449769 | B(iii) | 465.206 | 447.1217141 | POS | Isoflavonoids | 1.44 | 0.31 | 0.13 |
| 470 | 4'-hydroxy-5,7-dimethoxy-8-methylflavan | 0.765321846 | B(iii) | 583.337 | 301.1409852 | POS | Flavonoids | 0.08 | 0.09 | 0.06 |
| 471 | phellopterin | 0.765 | B(iii) | 351.393 | 323.0886433 | POS | Coumarins | 0.89 | 0.84 | 0.41 |
| 472 | kanakugiol | 0.764645154 | B(iii) | 476.955 | 343.118091 | NEG | Flavonoids | 0.02 | 0.19 | 0.04 |
| 473 | 7-hydroxy-2-(4-hydroxy-3,5-dimethoxyphenyl)-5-[(2s,3r,4s,5s,6r)-3,4,5-trihydroxy-6-(hydroxymethyl)oxan-2-yl]oxychromen-4-one | 0.764405 | B(iii) | 383.5175 | 493.1283067 | POS | Flavonoids | 0.39 | 1.00 | 0.03 |
| 474 | 5-hydroxy-6-methoxy-2-(4-methoxyphenyl)-7-(((2s,3r,4s,5s,6r)-3,4,5-trihydroxy-6-((((2r,3r,4r,5r,6s)-3,4,5-trihydroxy-6-methyltetrahydro-2h-pyran-2-yl)oxy)methyl)tetrahydro-2h-pyran-2-yl)oxy)-4h-chrome n-4-one | 0.763483 | B(iii) | 376.969 | 623.1871282 | POS | Flavonoids | 0.48 | 0.39 | 0.32 |
| 475 | (2~{r},3~{s},4~{s},5~{r},6~{s})-2-[[(2~{r},3~{r},4~{r})-3,4-dihydroxy-4-(hydroxymethyl)oxolan-2-yl]oxymethyl]-6-(2-methylbut-3-en-2-yloxy)oxane-3,4,5-triol | 0.762480846 | B(iii) | 487.07 | 403.1539423 | POS |  | 0.77 | 0.14 | 0.24 |
| 476 | 3,7,4'-trihydroxyflavone | 0.762288077 | B(iii) | 464.3085 | 269.0450111 | NEG | Flavonoids | 0.02 | 0.25 | 0.00 |
| 477 | 5,7-dihydroxy-2-(4-hydroxyphenyl)-6-[3,4,5-trihydroxy-6-(hydroxymethyl)oxan-2-yl]-8-(3,4,5-trihydroxyoxan-2-yl)chromen-4-one | 0.761276615 | B(iii) | 48.83975 | 587.1490142 | POS | Flavonoids | 0.03 | 0.03 | 0.05 |
| 478 | glaucarubin | 0.760458692 | B(iii) | 303.353 | 495.2324349 | NEG | Triterpenoids | 0.02 | 0.02 | 0.01 |
| 479 | acetoacetic acid | 0.759849692 | B(iii) | 46.2169 | 85.02822896 | POS | Fatty Acids and Conjugates | 0.23 | 0.23 | 0.26 |
| 480 | mangostin | 0.759690923 | B(iii) | 453.477 | 433.1629355 | POS | Xanthones | 0.62 | 0.45 | 0.27 |
| 481 | 3,4-dihydroxyhydrocinnamic acid | 0.758788769 | B(i) | 219.11 | 181.0502389 | NEG |  | 0.02 | 0.02 | 0.03 |
| 482 | pentadecanoic acid | 0.758511846 | B(iii) | 647.94 | 241.2166766 | NEG | Fatty Acids and Conjugates | 1.36 | 0.94 | 0.93 |
| 483 | 3,7,21-trihydroxy-17,17-dimethyl-14-(3-methylbut-2-en-1-yl)-10,12,16-trioxapentacyclo[11.8.0.0,.0,.0,henicosa-1(13),4(9),5,7,14,18,20-heptaen-2-one | 0.758221615 | B(iii) | 524.352 | 435.1431901 | NEG |  | 0.01 | 0.03 | 0.03 |
| 484 | spinosin | 0.757429385 | B(iii) | 321.363 | 609.1712689 | POS | Flavonoids | 0.06 | 0.10 | 0.10 |
| 485 | heralenol | 0.756438385 | B(iii) | 294.31 | 303.0869369 | NEG | Coumarins | 0.02 | 0.03 | 0.03 |
| 486 | phrymarolin i | 0.755886231 | B(iii) | 422.795 | 489.1300082 | POS | Lignans | 0.79 | 0.56 | 2.98 |
| 487 | [(2r,3s,4s,5r,6r)-6-[(2s,3s,4s,5r)-3,4-dihydroxy-2,5-bis(hydroxymethyl)oxolan-2-yl]oxy-3,4,5-trihydroxyoxan-2-yl]methyl (e)-3-(4-hydroxy-3,5-dimethoxyphenyl)prop-2-enoate | 0.755557462 | B(iii) | 477.62 | 549.1878153 | POS | Phenylpropanoids | 0.04 | 0.23 | 0.01 |
| 488 | broussin | 0.755116231 | B(iii) | 446.5195 | 257.1168284 | POS | Flavonoids | 1.88 | 1.03 | 1.40 |
| 489 | 2-[2-(3,4-dihydroxyphenyl)ethoxy]-6-(hydroxymethyl)oxane-3,4,5-triol | 0.754910077 | B(iii) | 219.358 | 334.1491761 | POS |  | 0.07 | 0.06 | 0.12 |
| 490 | 5-[(2r,3s)-6-hydroxy-2-(4-hydroxyphenyl)-4-[(e)-2-(4-hydroxyphenyl)ethenyl]-2,3-dihydro-1-benzofuran-3-yl]benzene-1,3-diol | 0.753683308 | B(iii) | 375.629 | 455.1487125 | POS | Stilbenoids | 0.04 | 0.05 | 0.01 |
| 491 | 8-hydroxy-5,8a-dimethyl-3-methylidene-3a,4,4a,8,9,9a-hexahydrobenzo[f][1]benzofuran-2,7-dione | 0.753190923 | B(iii) | 621.31 | 285.111178 | POS | Sesquiterpenoids | 0.33 | 0.05 | 0.05 |
| 492 | 5,5-dimethyl-2(5h)-furanone | 0.753096077 | B(iii) | 245.5885 | 113.0593053 | POS |  | 0.20 | 0.16 | 0.18 |
| 493 | 4'-methoxyflavone | 0.753009 | B(iii) | 373.0375 | 253.0852113 | POS | Flavonoids | 2.91 | 1.17 | 0.16 |
| 494 | dodecanoic acid | 0.752083385 | B(iii) | 571.003 | 199.1697337 | NEG | Fatty Acids and Conjugates | 0.25 | 0.24 | 0.22 |
| 495 | 8'-hydroxyabscisate | 0.751735 | B(iii) | 386.813 | 281.1372413 | POS | Apocarotenoids | 0.01 | 0.03 | 0.04 |
| 496 | 2e,4e-hexadecadienoic acid | 0.751635538 | B(iii) | 639.022 | 253.215691 | POS | Fatty Acids and Conjugates | 0.01 | 0.02 | 0.02 |
| 497 | pterosin h | 0.751529462 | B(iii) | 315.418 | 251.1249768 | POS | Sesquiterpenoids | 0.20 | 0.12 | 0.06 |
| 498 | anhydroicaritin | 0.750940385 | B(iii) | 283.667 | 369.1309983 | POS | Flavonoids | 0.20 | 0.04 | 0.01 |
| 499 | 5,7-dihydroxy-4-(4-methoxyphenyl)-3,4-dihydrochromen-2-one | 0.750407923 | B(iii) | 355.299 | 325.0466343 | POS | Flavonoids | 4.93 | 2.05 | 1.50 |
| 500 | stearamide | 0.749147769 | B(iii) | 686.86 | 284.2938062 | POS |  | 25.45 | 20.78 | 29.36 |
| 501 | tephrorianin | 0.748956538 | B(iii) | 466.648 | 421.1280849 | POS | Flavonoids | 0.02 | 0.78 | 0.00 |
| 502 | 3,7,3',4'-tetrahydroxyflavanone | 0.748790385 | B(iii) | 338.1275 | 287.0557948 | NEG | Flavonoids | 0.03 | 0.03 | 0.03 |
| 503 | 1-(3-hydroxy-4-methoxyphenyl)-1,2-ethanediol | 0.747065231 | B(iii) | 258.114 | 185.0804776 | POS |  | 0.64 | 0.28 | 0.14 |
| 504 | 7-o-methylrosmanol | 0.746916846 | B(iii) | 378.481 | 359.1849316 | NEG | Diterpenoids | 0.02 | 0.04 | 0.11 |
| 505 | [2-[(2-benzamido-3-phenylpropanoyl)amino]-3-phenylpropyl] acetate | 0.746574385 | B(iii) | 474.3865 | 467.1962111 | POS |  | 0.05 | 0.09 | 0.15 |
| 506 | 3-hydroxy-4-methoxyxanthen-9-one | 0.746450308 | B(iii) | 340.214 | 243.0643039 | POS |  | 1.11 | 0.59 | 1.80 |
| 507 | chrysin | 0.745124769 | B(iii) | 358.6015 | 255.0646973 | POS | Flavonoids | 1.56 | 0.88 | 0.46 |
| 508 | glutathione | 0.744770462 | B(iii) | 47.5489 | 325.1118515 | POS | Small peptides | 0.36 | 0.46 | 0.45 |
| 509 | 4-hydroxydihydrocinnamaldehyde | 0.744334923 | B(iii) | 479.677 | 151.0750371 | POS |  | 1.59 | 0.40 | 0.20 |
| 510 | 2,6-dimethylphenol | 0.744282154 | B(iii) | 785.589 | 123.0841885 | POS |  | 0.51 | 0.52 | 0.55 |
| 511 | artonin o | 0.741605154 | B(iii) | 459.427 | 503.2048832 | POS | Flavonoids | 0.01 | 0.27 | 0.00 |
| 512 | haplopappin | 0.741407154 | B(iii) | 305.743 | 435.1433361 | POS | Flavonoids | 0.07 | 0.02 | 0.01 |
| 513 | hispidulin 7-glucoside | 0.740965 | B(iii) | 44.2377 | 463.1331245 | POS | Flavonoids | 0.06 | 0.19 | 0.15 |
| 514 | cis-mulberroside a | 0.740273231 | B(iii) | 387.446 | 569.1799081 | POS | Stilbenoids | 0.78 | 1.23 | 0.45 |
| 515 | protocatechuic acid | 0.738978462 | B(iii) | 27.47315 | 177.0068069 | POS | Phenolic acids | 3.06 | 3.91 | 4.40 |
| 516 | tephrosin | 0.738711154 | B(iii) | 302.4245 | 409.1277419 | NEG | Isoflavonoids | 0.01 | 0.00 | 0.00 |
| 517 | 7-methoxy-6-(1,2,3-trihydroxy-3-methylbutyl)chromen-2-one | 0.737890231 | B(iii) | 393.2345 | 317.101104 | POS | Coumarins | 0.74 | 3.01 | 0.25 |
| 518 | abietic acid | 0.737779385 | B(iii) | 635.795 | 301.2169161 | NEG | Diterpenoids | 0.05 | 0.03 | 0.02 |
| 519 | 3,7-dihydroxy-9-methoxy-1-methylbenzo[c]chromen-6-one | 0.737321231 | B(iii) | 502.791 | 273.0748662 | POS |  | 0.09 | 0.13 | 0.64 |
| 520 | (2~{s},3~{r},4~{s},5~{s},6~{r})-2-[4-[(3~{s},3~{a}~{r},6~{s},6~{a}~{r})-3-(4-hydroxy-3,5-dimethoxyphenyl)-1,3,3~{a},4,6,6~{a}-hexahydrofuro[3,4-c]furan-6-yl]-2,6-dimethoxyphenoxy]-6-(hydroxymethyl)oxane-3,4,5-triol | 0.737101 | B(iii) | 309.835 | 598.2496414 | POS |  | 2.02 | 0.52 | 0.82 |
| 521 | aurantiamide acetate | 0.736234385 | B(iii) | 484.906 | 445.2122947 | POS |  | 2.17 | 0.20 | 0.12 |
| 522 | 5-hydroxyferulate | 0.735753692 | B(iii) | 649.516 | 209.0451101 | NEG | Phenylpropanoids | 0.01 | 0.01 | 0.01 |
| 523 | 5,7,4'-trimethoxyflavone | 0.734760769 | B(iii) | 379.1875 | 313.1062046 | POS | Flavonoids | 1.43 | 1.72 | 6.74 |
| 524 | 3-(3,4-dihydroxyphenyl)-5,7-dihydroxy-6,8-bis(3-methylbut-2-enyl)chromen-4-one | 0.734302 | B(iii) | 453.605 | 423.180381 | POS | Isoflavonoids | 0.62 | 0.36 | 0.16 |
| 525 | benzyl acetic acid | 0.733996846 | B(iii) | 355.085 | 149.0605275 | NEG |  | 0.02 | 0.03 | 0.03 |
| 526 | geniposide | 0.731589 | B(iii) | 515.9065 | 389.1486872 | POS | Monoterpenoids | 0.06 | 0.09 | 0.33 |
| 527 | paclitaxel | 0.731184846 | B(iii) | 516.48 | 854.3206373 | POS | Others | 0.06 | 1.31 | 0.00 |
| 528 | beta-glucose pentaacetic acid | 0.730807 | B(iii) | 386.7115 | 413.0992439 | POS | Saccharides | 0.08 | 0.25 | 0.07 |
| 529 | d-asparagine | 0.730707462 | B(i) | 512.633 | 133.064533 | POS | Small peptides | 0.01 | 0.02 | 0.02 |
| 530 | 4-methylabyssinone v | 0.729865154 | B(iii) | 539.637 | 423.2246282 | POS | Isoflavonoids | 0.15 | 0.11 | 0.11 |
| 531 | euchrenone a2 | 0.729585769 | B(iii) | 488.917 | 407.1838267 | POS | Flavonoids | 12.60 | 6.42 | 0.06 |
| 532 | docosanoic acid | 0.729331231 | B(iii) | 751.077 | 339.3257906 | NEG | Fatty Acids and Conjugates | 0.11 | 0.10 | 0.07 |
| 533 | 5,7-dihydroxy-2'-methoxyflavone | 0.728855769 | B(iii) | 329.6585 | 283.0601936 | NEG | Flavonoids | 1.53 | 2.99 | 0.46 |
| 534 | 4-hydroxy-2',4'-dimethoxydihydrochalcone | 0.728235077 | B(iii) | 344.782 | 287.127542 | POS | Flavonoids | 0.06 | 0.26 | 0.11 |
| 535 | beta-sinensal | 0.727576769 | B(iii) | 541.968 | 219.1740832 | POS | Sesquiterpenoids | 0.05 | 0.21 | 0.03 |
| 536 | 1-monostearin | 0.727261538 | B(iii) | 635.781 | 359.3153458 | POS | Glycerolipids | 0.03 | 0.05 | 0.02 |
| 537 | gummiferol | 0.726173462 | B(iii) | 302.423 | 287.090632 | POS |  | 0.15 | 0.52 | 0.21 |
| 538 | sterebin a | 0.726145 | B(iii) | 367.081 | 311.2206798 | POS | Diterpenoids | 0.14 | 0.15 | 0.55 |
| 539 | 4-guanidinobutanoic acid | 0.725767 | B(iii) | 49.1005 | 146.0921276 | POS | Small peptides | 0.20 | 0.26 | 0.07 |
| 540 | [(2~{r},3~{r},4~{s},5~{r},6~{r})-6-[2-(3,4-dihydroxyphenyl)ethoxy]-3,5-dihydroxy-4-[(2~{s},3~{r},4~{r},5~{r},6~{s})-3,4,5-trihydroxy-6-methyloxan-2-yl]oxyoxan-2-yl]methyl (~{e})-3-(4-hydroxy-3-methoxyphenyl)prop-2-enoate | 0.725448308 | B(iii) | 200.143 | 656.2378583 | POS |  | 0.01 | 0.01 | 0.02 |
| 541 | dihydroferulic acid | 0.725303154 | B(iii) | 270.7495 | 195.0656005 | NEG | Phenolic acids | 0.04 | 0.05 | 0.03 |
| 542 | limonin | 0.725261077 | B(iii) | 250.927 | 493.1703985 | POS | Triterpenoids | 0.04 | 0.07 | 0.01 |
| 543 | malonylglycitin | 0.723685692 | B(i) | 338.5135 | 533.1190588 | POS | Isoflavonoids | 0.21 | 0.18 | 0.05 |
| 544 | mollugin | 0.722696154 | B(iii) | 310.473 | 285.1109283 | POS | Naphthalenes | 0.08 | 0.10 | 0.05 |
| 545 | l-sorbose | 0.722469385 | B(iii) | 45.0065 | 161.0452845 | NEG | Saccharides | 0.05 | 0.04 | 0.05 |
| 546 | coniferyl alcohol | 0.72135 | B(iii) | 523.6325 | 167.0697824 | POS | Phenylpropanoids | 0.31 | 0.67 | 0.06 |
| 547 | oleanoic acid | 0.720992077 | B(iii) | 744.322 | 455.3525826 | NEG | Triterpenoids | 0.01 | 0.01 | 0.00 |
| 548 | 5,7-dihydroxy-2-(4-hydroxyphenyl)-6,8-bis(3,4,5-trihydroxyoxan-2-yl)chromen-4-one | 0.719997154 | B(iii) | 376.278 | 535.1353301 | POS |  | 0.16 | 0.18 | 0.17 |
| 549 | 4'-hydroxy-7-methoxy-8-methylflavan | 0.719986615 | B(iii) | 479.6375 | 271.1319422 | POS | Flavonoids | 1.63 | 2.29 | 0.30 |
| 550 | 6-methoxy-7-(3-methylbut-2-enoxy)chromen-2-one | 0.719849 | B(iii) | 319.071 | 259.0607402 | NEG | Coumarins | 0.01 | 0.02 | 0.03 |
| 551 | mimosine | 0.719816769 | B(iii) | 306.817 | 221.0570701 | POS | Small peptides | 0.09 | 2.95 | 1.02 |
| 552 | kumatakenin | 0.719142154 | B(iii) | 314.788 | 315.0860478 | POS | Flavonoids | 0.05 | 0.04 | 0.05 |
| 553 | beta-peltatin | 0.719099846 | B(iii) | 48.99655 | 453.1006025 | POS | Lignans | 0.05 | 0.05 | 0.06 |
| 554 | n-hexadecanoylpyrrolidine | 0.718739615 | B(iii) | 691.552 | 310.3096126 | POS | Fatty amides | 3.79 | 3.18 | 4.86 |
| 555 | lusitanicoside | 0.718347154 | B(iii) | 316.772 | 460.2176927 | POS | Phenylpropanoids | 0.04 | 0.02 | 0.04 |
| 556 | 7,8-dimethoxy-2h-chromen-2-one | 0.717284077 | B(iii) | 349.426 | 207.0646461 | POS | Coumarins | 0.18 | 0.09 | 0.10 |
| 557 | methylophiopogonanone b | 0.716606846 | B(iii) | 672.569 | 329.137696 | POS | Isoflavonoids | 0.19 | 0.13 | 0.01 |
| 558 | 3,7-dimethyluric acid | 0.715507077 | B(iii) | 41.4738 | 195.0506937 | NEG | Pseudoalkaloids | 0.17 | 0.28 | 0.08 |
| 559 | 6,11-dihydroxy-2,2-dimethylpyrano[3,2-c]xanthen-7(2h)-one | 0.715139692 | B(iii) | 354.5765 | 311.0903551 | POS | Xanthones | 3.71 | 3.83 | 1.24 |
| 560 | lupenone | 0.714394231 | B(iii) | 773.879 | 425.3779169 | POS | Triterpenoids | 0.23 | 0.09 | 0.04 |
| 561 | rotenone | 0.714374 | B(iii) | 355.314 | 395.1472282 | POS | Isoflavonoids | 0.08 | 0.10 | 0.05 |
| 562 | linoelaidic acid | 0.713740846 | B(iii) | 669.335 | 281.2464323 | POS | Fatty Acids and Conjugates | 0.19 | 0.19 | 0.13 |
| 563 | ethyl vanillin | 0.713606615 | B(iii) | 546.6455 | 167.0698148 | POS | Phenolic acids | 0.15 | 0.28 | 0.06 |
| 564 | 1',2'-dihydro-2',6-dihydroxyrotenone | 0.713535846 | B(iii) | 328.447 | 451.1382763 | POS | Isoflavonoids | 0.02 | 0.04 | 0.24 |
| 565 | (2s,3r,4s,5r)-2-[(2r,3r,4s,5s,6r)-4,5-dihydroxy-6-(hydroxymethyl)-2-(2-phenylethoxy)oxan-3-yl]oxyoxane-3,4,5-triol | 0.713481538 | B(iii) | 268.81 | 434.200701 | POS | Phenylethanoids | 0.09 | 0.02 | 0.07 |
| 566 | (9-hydroxy-8,8-dimethyl-2-oxo-9,10-dihydropyrano[2,3-f]chromen-10-yl) acetate | 0.713470538 | B(iii) | 316.68 | 305.1005733 | POS |  | 0.05 | 0.07 | 0.06 |
| 567 | 3-phenyllactic acid | 0.712196692 | B(iii) | 434.151 | 167.0697345 | POS |  | 1.61 | 1.98 | 0.87 |
| 568 | 6-[(2~{s},3~{r},4~{s},5~{s},6~{r})-4,5-dihydroxy-6-(hydroxymethyl)-3-[(2~{s},3~{r},4~{r},5~{r},6~{s})-3,4,5-trihydroxy-6-methyloxan-2-yl]oxyoxan-2-yl]-5,7-dihydroxy-2-(4-hydroxyphenyl)chromen-4-one | 0.712099923 | B(iii) | 605.512 | 601.1622186 | POS |  | 0.00 | 0.25 | 0.15 |
| 569 | austricine | 0.711204538 | B(iii) | 608.2015 | 263.1267318 | POS | Sesquiterpenoids | 0.04 | 0.03 | 0.00 |
| 570 | glycycoumarin | 0.710963538 | B(iii) | 386.674 | 391.1166121 | POS | Coumarins | 0.22 | 0.25 | 0.32 |
| 571 | furostane base -2h + 1o, o-hex, o-pen-dhex | 0.710779077 | B(iii) | 309.431 | 871.4605601 | POS | Steroids | 1.47 | 0.33 | 0.22 |
| 572 | 2'-hydroxy-4',6'-dimethoxy-3'-methylacetophenone | 0.709886923 | B(iii) | 325.178 | 211.096021 | POS | Phloroglucinols | 0.31 | 0.15 | 0.03 |
| 573 | 2-(3-hydroxy-5-methoxyphenoxy)-6-(hydroxymethyl)oxane-3,4,5-triol | 0.709812846 | B(iii) | 456.7675 | 325.082542 | POS | Phloroglucinols | 44.15 | 32.35 | 2.69 |
| 574 | arborinine | 0.709156231 | B(iii) | 373.0455 | 286.104103 | POS | Anthranilic acid alkaloids | 0.15 | 0.20 | 0.12 |
| 575 | isorhamnetin-3-o-galactoside-6''-rhamnoside | 0.708792462 | B(iii) | 287.337 | 625.1760197 | POS | Flavonoids | 0.01 | 0.00 | 0.01 |
| 576 | l-arabitol | 0.708168077 | B(iii) | 44.9501 | 153.0754008 | POS | Saccharides | 0.31 | 0.39 | 0.35 |
| 577 | norlichexanthone | 0.707852231 | B(iii) | 429.773 | 257.0445078 | NEG | Xanthones | 0.00 | 0.01 | 0.11 |
| 578 | (2~{r},3~{r},4~{s},5~{s},6~{r})-2-[[(2~{r},3~{r},4~{s})-6-hydroxy-4-(4-hydroxy-3-methoxyphenyl)-3-(hydroxymethyl)-7-methoxy-1,2,3,4-tetrahydronaphthalen-2-yl]methoxy]-6-(hydroxymethyl)oxane-3,4,5-triol | 0.707558 | B(iii) | 435.171 | 545.2149892 | POS |  | 24.70 | 24.01 | 13.42 |
| 579 | (2r,3r)-2-(3,4-dihydroxyphenyl)-3,5-dihydroxy-7-methoxy-2,3-dihydrochromen-4-one | 0.707412385 | B(iii) | 332.976 | 317.0656123 | NEG | Flavonoids | 0.02 | 0.19 | 0.02 |
| 580 | 1-linoleoyl glycerol | 0.707323 | B(iii) | 525.262 | 355.2837526 | POS | Glycerolipids | 0.29 | 0.28 | 0.18 |
| 581 | 4-hydroxybenzoic acid | 0.706215615 | B(iii) | 315.913 | 137.0240435 | NEG | Phenolic acids | 0.04 | 0.03 | 0.02 |
| 582 | testosterone | 0.704972923 | B(iii) | 504.035 | 311.1994676 | POS | Steroids | 0.02 | 0.04 | 0.08 |
| 583 | leridal | 0.704157231 | B(iii) | 531.545 | 313.1062983 | POS | Flavonoids | 0.03 | 0.09 | 0.09 |
| 584 | 1,3,6-trihydroxy-2-(3-methylbut-2-enyl)xanthen-9-one | 0.703767923 | B(iii) | 381.4 | 311.0912763 | NEG | Xanthones | 0.01 | 0.01 | 0.12 |
| 585 | 1,3,5-trihydroxy-10-methylacridone | 0.703568692 | B(iii) | 344.1285 | 258.0729129 | POS | Anthranilic acid alkaloids | 0.16 | 0.12 | 0.07 |
| 586 | 4-nitrophenol | 0.703295154 | B(iii) | 356.401 | 138.0192147 | NEG | Others | 0.01 | 0.01 | 0.01 |
| 587 | linoleic acid | 0.702966769 | B(iii) | 673.562 | 303.2314819 | POS | Fatty Acids and Conjugates | 0.05 | 0.05 | 0.02 |
| 588 | 3-hydroxy-9,10-dimethoxypterocarpan | 0.702702231 | B(iii) | 496.1255 | 301.1065321 | POS | Isoflavonoids | 0.43 | 1.93 | 0.21 |
| 589 | boeravinone c | 0.702348692 | B(iii) | 314.124 | 345.0956353 | POS | Isoflavonoids | 0.06 | 0.04 | 0.21 |
| 590 | desglucocoroloside | 0.702176308 | B(iii) | 559.383 | 505.3138793 | POS | Steroids | 0.06 | 0.02 | 0.02 |
| 591 | ergosterol peroxide_120246 | 0.701618385 | B(iii) | 683.1475 | 429.3349245 | POS | Steroids | 0.48 | 0.50 | 0.16 |
| 592 | 4-(1-methylethenyl)benzaldehyde | 0.701320615 | B(iii) | 306.525 | 147.0801909 | POS | Monoterpenoids | 0.02 | 0.46 | 0.13 |
| 593 | dihydroformononetin | 0.701204308 | B(iii) | 435.955 | 271.0956914 | POS | Isoflavonoids | 0.79 | 0.93 | 0.57 |
| 594 | (1s,4as,7s,7as)-7-hydroxy-7-methyl-1-[(2s,3r,4s,5s,6r)-3,4,5-trihydroxy-6-[(4-hydroxybenzoyl)oxymethyl]oxan-2-yl]oxy-4a,5,6,7a-tetrahydro-1h-cyclopenta[c]pyran-4-carboxylic acid | 0.699688923 | B(iii) | 425.603 | 519.1418182 | POS | Monoterpenoids | 0.69 | 0.75 | 1.40 |
| 595 | (-)-curcumol | 0.698831923 | B(iii) | 500.095 | 237.1846326 | POS | Sesquiterpenoids | 0.07 | 0.13 | 0.03 |
| 596 | (2e)-3,7-dimethyl-2,6-octadienyl acetate | 0.698794231 | B(iii) | 339.591 | 197.1529206 | POS | Fatty esters | 0.03 | 0.00 | 0.00 |
| 597 | alpha-tocopherol | 0.698583462 | B(iii) | 749.181 | 430.3752 | POS | Meroterpenoids | 0.18 | 0.19 | 0.11 |
| 598 | plaunotol | 0.698119615 | B(iii) | 616.109 | 307.262487 | POS | Sesterterpenoids | 0.02 | 0.02 | 0.03 |
| 599 | niloticin | 0.696832615 | B(iii) | 746.648 | 457.3660488 | POS | Triterpenoids | 0.04 | 0.04 | 0.03 |
| 600 | o-toluate | 0.696339154 | B(iii) | 332.5385 | 135.0447318 | NEG | Phenolic acids | 0.01 | 0.01 | 0.01 |
| 601 | flavone base + 3o, 1meo | 0.696222 | B(iii) | 342.28 | 299.0557032 | NEG | Flavonoids | 0.01 | 0.04 | 0.05 |
| 602 | 1-[2,4,6-trihydroxy-3-[7-hydroxy-2-(4-hydroxyphenyl)-3,4-dihydro-2h-chromen-4-yl]phenyl]dodecan-1-one | 0.695892538 | B(iii) | 674.927 | 547.2711725 | NEG | Flavonoids | 0.04 | 0.04 | 0.02 |
| 603 | 5,7-dihydroxy-2-(4-hydroxyphenyl)-8-[3,4,5-trihydroxy-6-(hydroxymethyl)oxan-2-yl]-6-(3,4,5-trihydroxyoxan-2-yl)chromen-4-one | 0.695368308 | B(iii) | 284.6 | 582.180474 | POS | Flavonoids | 0.02 | 0.03 | 0.00 |
| 604 | garciduol a | 0.695304 | B(iii) | 440.914 | 487.114121 | POS | Phloroglucinols | 0.07 | 0.18 | 0.09 |
| 605 | angoletin | 0.694838538 | B(iii) | 494.868 | 301.1426937 | POS | Flavonoids | 1.30 | 5.41 | 0.17 |
| 606 | 6-methyl-3e,5-heptadien-2-one | 0.694744308 | B(iii) | 594.346 | 125.0957427 | POS | Fatty acyls | 0.08 | 0.03 | 0.03 |
| 607 | 3-hydroxycapric acid | 0.693946923 | B(iii) | 353.2475 | 187.133582 | NEG | Fatty Acids and Conjugates | 0.01 | 0.02 | 0.01 |
| 608 | (3~{r},5~{s},8~{e})-5,9,14-trimethyl-4,12-dioxatricyclo[9.3.0.0^{3,5}]tetradeca-1(11),8,13-trien-2-one | 0.693579846 | B(iii) | 306.525 | 247.1321792 | POS |  | 1.23 | 0.31 | 1.29 |
| 609 | cnidilide | 0.692653 | B(iii) | 364.1545 | 195.1376544 | POS | Cyclic polyketides | 0.09 | 0.05 | 0.02 |
| 610 | l-sinoacutine | 0.692124154 | B(iii) | 409.075 | 328.1540906 | POS | Tyrosine alkaloids | 0.32 | 0.42 | 0.01 |
| 611 | s-acetyl dihydroasparagusic acid | 0.689353077 | B(iii) | 73.32175 | 195.0174009 | POS | Fatty Acids and Conjugates | 0.13 | 0.12 | 0.13 |
| 612 | 12:4+3o fatty acyl hexoside | 0.688803308 | B(iii) | 258.169 | 387.1643055 | NEG | Fatty acyl glycosides | 0.00 | 0.00 | 0.00 |
| 613 | sequiterpene lactone 326 | 0.688773769 | B(iii) | 486.371 | 263.1284552 | NEG | Sesquiterpenoids | 0.00 | 0.06 | 0.00 |
| 614 | furostane base -1h2o + 1o, o-hex, o-hex-hex | 0.687825154 | B(iii) | 303.518 | 917.4727151 | NEG | Steroids | 0.00 | 0.00 | 0.00 |
| 615 | 5,4'-dihydroxy-6,7-dimethoxyflavanone | 0.687327385 | B(iii) | 372.237 | 317.1014944 | POS | Flavonoids | 0.42 | 0.77 | 0.24 |
| 616 | sodium 3-(3,4-dihydroxyphenyl)-2-hydroxypropanoate | 0.686485769 | B(i) | 192.6835 | 243.0502482 | NEG |  | 0.01 | 0.00 | 0.01 |
| 617 | dulxanthone d | 0.685327615 | B(iii) | 443.393 | 343.1169386 | POS | Xanthones | 0.02 | 0.05 | 0.02 |
| 618 | sweroside | 0.685155692 | B(i) | 220.592 | 357.1177314 | NEG | Monoterpenoids | 0.00 | 0.00 | 0.00 |
| 619 | homoferreirin | 0.685149 | B(iii) | 438.294 | 317.1011602 | POS | Isoflavonoids | 0.13 | 0.35 | 0.09 |
| 620 | 6''-acetylliquiritin | 0.684990385 | B(iii) | 303.9275 | 461.1398734 | POS | Flavonoids | 0.79 | 0.60 | 0.37 |
| 621 | (2~{s},3~{r},4~{s},5~{s},6~{r})-2-[4-[(1~{s},2~{s},3~{s})-3,7-dihydroxy-2,3-bis(hydroxymethyl)-6-methoxy-2,4-dihydro-1~{h}-naphthalen-1-yl]-2-methoxyphenoxy]-6-(hydroxymethyl)oxane-3,4,5-triol | 0.684310462 | B(iii) | 522.864 | 556.2396406 | POS |  | 0.09 | 0.33 | 0.09 |
| 622 | fistuloside c | 0.684188154 | B(iii) | 330.276 | 917.4687869 | POS | Steroids | 0.03 | 0.03 | 0.02 |
| 623 | tetrahydropalmatine | 0.683456385 | B(iii) | 369.139 | 356.185401 | POS | Tyrosine alkaloids | 0.07 | 0.31 | 0.01 |
| 624 | undecanoate | 0.682260692 | B(iii) | 540.8145 | 185.1540264 | NEG | Fatty Acids and Conjugates | 0.12 | 0.12 | 0.11 |
| 625 | 4,4',alpha-trihydroxy-2'-methoxydihydrochalcone | 0.681331692 | B(iii) | 318.274 | 289.1061363 | POS | Flavonoids | 0.09 | 0.28 | 0.14 |
| 626 | cathine | 0.681243308 | B(iii) | 232.474 | 152.1067997 | POS | Pseudoalkaloids | 0.06 | 0.01 | 0.00 |
| 627 | heminitidulan | 0.680769308 | B(iii) | 537.537 | 407.2182717 | POS | Isoflavonoids | 0.33 | 0.33 | 0.27 |
| 628 | cirsimarin | 0.680483 | B(iii) | 426.589 | 477.1303412 | POS | Flavonoids | 0.11 | 0.28 | 0.27 |
| 629 | sinapoylmalate | 0.680243923 | B(iii) | 269.035 | 207.1371901 | POS | Phenylpropanoids | 0.02 | 0.04 | 0.02 |
| 630 | 5-aminoimidazole-4-carboxamide-1-beta-d-ribofuranosyl 5&#39;-monophosphate | 0.678283846 | B(iii) | 318.1675 | 339.0620992 | POS | Nucleosides | 6.60 | 7.75 | 7.40 |
| 631 | nicotiflorin | 0.677397231 | B(iii) | 286.023 | 595.1649312 | POS | Flavonoids | 0.01 | 0.01 | 0.00 |
| 632 | 1,6-anhydro-b-glucose | 0.677388 | B(iii) | 782.7665 | 161.0452606 | NEG | Saccharides | 0.01 | 0.01 | 0.01 |
| 633 | beta-cyclocitral | 0.677361 | B(iii) | 318.274 | 153.1270967 | POS | Apocarotenoids | 0.03 | 0.18 | 0.02 |
| 634 | d-(-)-quinic acid | 0.677166769 | B(iii) | 83.255 | 231.0262561 | POS | Phenolic acids | 0.08 | 0.08 | 0.35 |
| 635 | flavokawin a | 0.675606769 | B(iii) | 409.413 | 317.1378202 | POS | Flavonoids | 0.55 | 0.68 | 0.96 |
| 636 | geranyl formate | 0.675424538 | B(iii) | 405.8215 | 183.1375246 | POS | Monoterpenoids | 0.04 | 0.07 | 0.00 |
| 637 | oleamide | 0.674754077 | B(iii) | 664.62 | 282.278506 | POS | Fatty acyls | 3.85 | 3.06 | 4.63 |
| 638 | chaulmoogric acid | 0.674702308 | B(iii) | 610.7955 | 281.2466258 | POS | Fatty Acids and Conjugates | 0.13 | 0.13 | 0.14 |
| 639 | 2-(3,4-dimethoxyphenyl)-7-methoxy-4h-chromen-4-one | 0.674504231 | B(iii) | 509.6355 | 313.1063467 | POS | Flavonoids | 0.08 | 0.06 | 0.14 |
| 640 | cathasterone | 0.674212846 | B(iii) | 648.198 | 433.3661909 | POS | Steroids | 2.99 | 3.85 | 1.97 |
| 641 | gossypol | 0.673219385 | B(iii) | 310.2855 | 557.1538182 | POS | Sesquiterpenoids | 0.08 | 0.10 | 0.03 |
| 642 | myristicin | 0.672700923 | B(iii) | 324.591 | 193.0855146 | POS | Phenylpropanoids | 0.10 | 0.06 | 0.04 |
| 643 | 5-methoxyvestitol | 0.670701615 | B(iii) | 362.299 | 303.1221006 | POS | Isoflavonoids | 18.80 | 12.47 | 32.41 |
| 644 | rutacultin | 0.669935385 | B(iii) | 283.28 | 275.1248126 | POS | Coumarins | 0.58 | 0.55 | 0.58 |
| 645 | scutellarioside ii | 0.669911154 | B(iii) | 280.097 | 507.1493158 | NEG | Monoterpenoids | 0.00 | 0.00 | 0.00 |
| 646 | l-gamma-glutamyl-l-valine | 0.668888077 | B(iii) | 76.74505 | 247.128464 | POS | Small peptides | 0.02 | 0.01 | 0.02 |
| 647 | salviaflaside | 0.668494923 | B(iii) | 344.137 | 523.136932 | POS | Phenylpropanoids | 0.15 | 0.49 | 0.11 |
| 648 | typhaneoside | 0.668276615 | B(iii) | 365.134 | 771.2409883 | POS | Flavonoids | 0.07 | 0.14 | 0.02 |
| 649 | gamma-mangostin | 0.667197769 | B(iii) | 424.931 | 419.1486991 | POS | Xanthones | 0.46 | 0.57 | 0.58 |
| 650 | gibberellin a7 | 0.666542538 | B(iii) | 536.1945 | 313.1429486 | POS | Diterpenoids | 0.03 | 0.11 | 0.06 |
| 651 | citromitin | 0.666037462 | B(iii) | 335.196 | 405.1520052 | POS | Flavonoids | 0.47 | 0.43 | 0.51 |
| 652 | gigantol | 0.665397846 | B(iii) | 477.0575 | 297.111266 | POS | Stilbenoids | 0.59 | 0.58 | 0.15 |
| 653 | ethyl 3-(methylthio)propanoate | 0.665319846 | B(iii) | 419.6665 | 149.0592478 | POS | Fatty esters | 0.10 | 0.64 | 0.02 |
| 654 | phthalic anhydride | 0.665281077 | B(iii) | 583.348 | 149.0231068 | POS |  | 0.17 | 0.16 | 0.23 |
| 655 | 4-hydroxy-3-tetratrenylbenzoic acid | 0.665148769 | B(iii) | 497.669 | 411.2886871 | POS | Diterpenoids | 0.97 | 1.35 | 0.33 |
| 656 | piperin | 0.663647846 | B(iii) | 398.616 | 286.114879 | POS | Lysine alkaloids | 0.04 | 0.05 | 0.05 |
| 657 | eugenin | 0.663575769 | B(iii) | 315.989 | 207.0647801 | POS | Chromanes | 0.15 | 0.09 | 0.10 |
| 658 | irigenin | 0.663573 | B(iii) | 49.3353 | 359.074214 | NEG | Isoflavonoids | 0.04 | 0.03 | 0.02 |
| 659 | 2-[3-[4-[1,3-dihydroxy-1-(4-hydroxy-3-methoxyphenyl)propan-2-yl]oxy-3-methoxyphenyl]propoxy]-6-(hydroxymethyl)oxane-3,4,5-triol | 0.663531769 | B(iii) | 481.593 | 558.2488524 | POS |  | 0.08 | 0.04 | 0.13 |
| 660 | 4-acetamidobutanoate | 0.663406385 | B(iii) | 419.423 | 168.0731163 | POS | Fatty Acids and Conjugates | 0.48 | 3.32 | 0.02 |
| 661 | melezitose | 0.661656538 | B(iii) | 554.338 | 522.1979335 | POS | Saccharides | 0.57 | 0.22 | 0.09 |
| 662 | methyl (4~{s},5~{e},6~{s})-5-ethylidene-4-[2-oxo-2-[(2~{s},3~{r},4~{s},5~{s},6~{r})-3,4,5-trihydroxy-6-(hydroxymethyl)oxan-2-yl]oxyethyl]-6-[(2~{s},3~{r},4~{s},5~{s},6~{r})-3,4,5-trihydroxy-6-(hydroxymethyl)oxan-2-yl]oxy-4~{h}-pyran-3-carboxylate | 0.661649769 | B(iii) | 309.6625 | 584.2328735 | POS |  | 0.11 | 0.11 | 0.03 |
| 663 | l-asparagine | 0.661202692 | B(iii) | 321.6025 | 113.0353243 | NEG | Small peptides | 0.01 | 0.00 | 0.00 |
| 664 | 1-[3-methoxy-4-[(2s,3r,4s,5s,6r)-3,4,5-trihydroxy-6-[[(2r,3r,4r,5r,6s)-3,4,5-trihydroxy-6-methyloxan-2-yl]oxymethyl]oxan-2-yl]oxyphenyl]ethanone | 0.660891231 | B(iii) | 293.8 | 473.1659839 | NEG | Others | 0.01 | 0.02 | 0.01 |
| 665 | inermin | 0.660529077 | B(iii) | 288.853 | 307.056877 | POS | Isoflavonoids | 0.07 | 0.08 | 0.05 |
| 666 | 12(r)-hepe | 0.660072077 | B(iii) | 677.817 | 301.2157169 | POS | Eicosanoids | 0.04 | 0.04 | 0.01 |
| 667 | dichotosin | 0.658786077 | B(iii) | 340.255 | 449.1772062 | POS | Flavonoids | 0.35 | 0.30 | 0.34 |
| 668 | alpha-cyperone | 0.658085231 | B(iii) | 590.472 | 219.1740224 | POS | Sesquiterpenoids | 0.11 | 0.05 | 0.01 |
| 669 | (z)-5-hexadecenoic acid | 0.658017462 | B(iii) | 585.368 | 255.2313822 | POS | Fatty Acids and Conjugates | 0.02 | 0.04 | 0.05 |
| 670 | chalepensin | 0.657991538 | B(iii) | 421.298 | 255.1006559 | POS | Coumarins | 0.75 | 0.36 | 0.67 |
| 671 | confertifoline | 0.657943 | B(iii) | 495.243 | 233.1543211 | NEG | Sesquiterpenoids | 0.02 | 0.02 | 0.01 |
| 672 | andrographidin a | 0.657925846 | B(iii) | 290.121 | 463.1597574 | POS | Flavonoids | 0.04 | 0.90 | 0.01 |
| 673 | 7,4'-dihydroxy-3'-methoxyflavan | 0.657839692 | B(iii) | 358.428 | 273.1110539 | POS | Flavonoids | 0.28 | 0.15 | 0.62 |
| 674 | 4-hydroxystachydrine | 0.656186462 | B(iii) | 45.4886 | 160.0963353 | POS | Others | 0.06 | 0.22 | 0.05 |
| 675 | shanzhiside | 0.655883769 | B(iii) | 276.854 | 391.1384141 | NEG | Monoterpenoids | 0.01 | 0.01 | 0.01 |
| 676 | loureirin d | 0.655006538 | B(iii) | 408.4405 | 289.1064161 | POS | Flavonoids | 1.82 | 2.23 | 0.96 |
| 677 | deoxytubulosine | 0.654753615 | B(iii) | 516.492 | 460.2936506 | POS | Tryptophan alkaloids | 0.03 | 0.43 | 0.03 |
| 678 | (2r,3s,4s,5r,6s)-2-(hydroxymethyl)-6-(3,4,5-trimethoxyphenoxy)oxane-3,4,5-triol | 0.654547154 | B(iii) | 266.191 | 345.1187737 | NEG | Phloroglucinols | 0.01 | 0.00 | 0.01 |
| 679 | isoeleutherin | 0.654433154 | B(iii) | 328.414 | 273.1109691 | POS | Naphthalenes | 0.83 | 0.41 | 0.55 |
| 680 | p-hydroxypropiophenone | 0.654206 | B(iii) | 403.133 | 151.0747878 | POS |  | 0.09 | 0.01 | 0.47 |
| 681 | abscisic acid | 0.654091308 | B(iii) | 549.485 | 263.1286403 | NEG | Apocarotenoids | 0.00 | 0.01 | 0.00 |
| 682 | [2-[(2~{s},3~{r},4~{s},5~{s},6~{r})-3,4,5-trihydroxy-6-(hydroxymethyl)oxan-2-yl]oxyphenyl]methyl 2-hydroxy-6-[(2~{s},3~{r},4~{s},5~{s},6~{r})-3,4,5-trihydroxy-6-(hydroxymethyl)oxan-2-yl]oxybenzoate | 0.653560385 | B(iii) | 384.134 | 585.17385 | POS |  | 0.21 | 0.67 | 0.11 |
| 683 | vulpinic acid | 0.653348385 | B(iii) | 309.2115 | 345.0724309 | POS | Diazotetronic acids and derivatives | 0.03 | 0.07 | 0.06 |
| 684 | 2-hydroxyhomopterocarpin | 0.653106154 | B(iii) | 407.136 | 315.0860175 | POS | Isoflavonoids | 0.63 | 0.24 | 0.11 |
| 685 | gluconolactone | 0.652502385 | B(i) | 40.9849 | 179.0546395 | POS | Saccharides | 0.12 | 0.19 | 0.04 |
| 686 | 1-phenylethyl acetate | 0.652373 | B(iii) | 479.651 | 165.0904065 | POS | Others | 0.26 | 0.12 | 0.18 |
| 687 | retinaldehyde | 0.652365462 | B(iii) | 666.59 | 285.2208266 | POS | Diterpenoids | 0.04 | 0.04 | 0.03 |
| 688 | xanthopterin | 0.651839615 | B(iii) | 271.069 | 197.0807062 | POS | Pseudoalkaloids | 0.04 | 0.05 | 0.04 |
| 689 | diffutin | 0.651724923 | B(iii) | 265.995 | 465.1739333 | POS | Flavonoids | 0.02 | 0.01 | 0.01 |
| 690 | histidine | 0.651645231 | B(iii) | 431.78 | 331.1176173 | NEG | Small peptides | 0.15 | 0.62 | 0.02 |
| 691 | sanggenol l | 0.650205231 | B(iii) | 518.4645 | 423.1801308 | POS | Flavonoids | 2.28 | 1.40 | 0.02 |
| 692 | 5-hydroxy-7,3',4'-trimethoxy-6-c-methylflavanone | 0.64995 | B(iii) | 431.915 | 345.1323795 | POS | Flavonoids | 0.15 | 0.08 | 0.10 |
| 693 | 5,7-dihydroxy-2-(4-hydroxy-3-methoxyphenyl)-2,3-dihydrochromen-4-one | 0.648619538 | B(iii) | 190.869 | 303.0840052 | POS | Flavonoids | 0.11 | 0.12 | 0.05 |
| 694 | dihydrocalythropsin | 0.648570231 | B(iii) | 298.762 | 289.1061494 | POS | Others | 0.15 | 0.13 | 0.23 |
| 695 | (3r)-3-(3,4-dimethoxyphenyl)-8-hydroxy-3,4-dihydroisochromen-1-one | 0.648047692 | B(iii) | 423.435 | 301.1064378 | POS | Coumarins | 1.25 | 1.92 | 1.07 |
| 696 | 7,8,3',4'-tetramethoxy-6'',6''-dimethylpyrano[2'',3'':5,6]flavone | 0.647276538 | B(iii) | 401.223 | 425.1591984 | POS | Flavonoids | 0.62 | 0.36 | 0.29 |
| 697 | 8-nonynoic acid | 0.645811846 | B(iii) | 310.136 | 155.106119 | POS | Fatty acyls | 0.02 | 0.01 | 0.02 |
| 698 | (2e,6e,10z)-12-hydroxy-10-(hydroxymethyl)-6-methyl-2-(4-methylpent-3-enyl)dodeca-2,6,10-trienoic acid | 0.644364154 | B(iii) | 546.668 | 335.2165537 | NEG | Diterpenoids | 0.02 | 0.02 | 0.03 |
| 699 | salidroside | 0.643807615 | B(i) | 218.1 | 318.1536649 | POS | Phenylethanoids | 0.03 | 0.01 | 0.04 |
| 700 | [(1r,5s,6s)-3-(hydroxymethyl)-5-[(z)-2-methylbut-2-enoyl]oxy-2-oxo-6-propan-2-ylcyclohex-3-en-1-yl] (e)-4-hydroxy-2-methylbut-2-enoate | 0.643792923 | B(iii) | 779.2285 | 419.1482395 | POS | Sesquiterpenoids | 0.17 | 0.18 | 0.16 |
| 701 | 3,4-di-o-caffeoylquinic acid | 0.643782231 | B(iii) | 443.0015 | 517.1241484 | POS | Phenylpropanoids | 0.24 | 1.61 | 0.11 |
| 702 | pyridoxamine 5'-phosphate | 0.643084692 | B(iii) | 198.113 | 247.0454939 | NEG | Nicotinic acid alkaloids | 0.01 | 0.00 | 0.00 |
| 703 | brosimone g | 0.643078462 | B(iii) | 442.608 | 421.1642257 | POS | Flavonoids | 0.77 | 0.43 | 1.86 |
| 704 | (e)-3-[4-methoxy-2-[(2s,3r,4s,5s,6r)-3,4,5-trihydroxy-6-(hydroxymethyl)oxan-2-yl]oxyphenyl]prop-2-enoic acid | 0.642655231 | B(iii) | 264.469 | 355.0947484 | NEG | Phenylpropanoids | 0.01 | 0.00 | 0.01 |
| 705 | coniferin | 0.642604615 | B(iii) | 48.8553 | 343.1429132 | POS | Phenylpropanoids | 0.20 | 0.20 | 0.10 |
| 706 | verimol j | 0.642304692 | B(iii) | 306.4735 | 183.1011453 | POS | Aromatic polyketides | 0.04 | 1.12 | 0.32 |
| 707 | hexamethylquercetagetin | 0.642265846 | B(iii) | 46.9375 | 401.1291562 | NEG | Flavonoids | 0.17 | 0.33 | 0.33 |
| 708 | 2-palmitoylglycerol | 0.642232231 | B(iii) | 583.735 | 331.2831065 | POS | Glycerolipids | 0.03 | 0.06 | 0.03 |
| 709 | sakuranetin | 0.642170692 | B(iii) | 416.192 | 287.0910892 | POS | Flavonoids | 1.74 | 10.22 | 1.15 |
| 710 | 3-(4-hydroxyphenyl)-1-propanol | 0.642107615 | B(iii) | 594.346 | 153.0908529 | POS |  | 0.21 | 0.08 | 0.07 |
| 711 | (3~{a}~{s},5~{a}~{s},9~{b}~{r})-5~{a},9-dimethyl-3-methylidene-3~{a},4,6,7,8,9~{b}-hexahydrobenzo[g][1]benzofuran-2,5-dione | 0.641888308 | B(iii) | 487.303 | 269.1169121 | POS |  | 0.43 | 0.38 | 1.65 |
| 712 | fa 18:2+3o | 0.641820692 | B(iii) | 405.661 | 327.2166019 | NEG | Octadecanoids | 0.03 | 0.03 | 0.05 |
| 713 | rosmic acid | 0.641485308 | B(iii) | 363.192 | 413.1581526 | POS | Diterpenoids | 0.22 | 0.16 | 0.11 |
| 714 | sphinganine | 0.640919385 | B(iii) | 779.414 | 302.3049356 | POS | Spingolipids | 0.13 | 0.04 | 0.02 |
| 715 | ambonane | 0.640508308 | B(iii) | 365.1635 | 325.1044908 | POS | Isoflavonoids | 3.28 | 2.61 | 3.68 |
| 716 | (2~{r},3~{r},4~{s},5~{s},6~{r})-2-[2-(4-methoxyphenyl)ethoxy]-6-[[(2~{s},3~{r},4~{s},5~{s})-3,4,5-trihydroxyoxan-2-yl]oxymethyl]oxane-3,4,5-triol | 0.640132846 | B(iii) | 225.59 | 464.2116494 | POS |  | 0.01 | 0.00 | 0.09 |
| 717 | calomelanol h | 0.639856077 | B(iii) | 420.449 | 403.1173988 | POS | Flavonoids | 0.54 | 3.46 | 0.15 |
| 718 | veratric acid | 0.639234308 | B(iii) | 283.079 | 181.0503903 | NEG | Phenolic acids | 0.05 | 0.08 | 0.03 |
| 719 | allantoin | 0.639162923 | B(iii) | 446.409 | 181.0278589 | POS | Others | 2.30 | 2.38 | 3.27 |
| 720 | dihydroisomilletenone methyl ether | 0.638922308 | B(iii) | 447.988 | 345.1323602 | POS | Flavonoids | 0.18 | 0.08 | 0.17 |
| 721 | quinquangulin | 0.638774231 | B(iii) | 263.967 | 287.0907548 | POS | Chromanes | 0.20 | 0.13 | 0.53 |
| 722 | piplartine | 0.638747385 | B(iii) | 219.1065 | 316.1118485 | NEG |  | 0.00 | 0.00 | 0.00 |
| 723 | pro | 0.637944 | B(iii) | 46.8534 | 116.0702416 | POS | Small peptides | 0.10 | 0.19 | 0.09 |
| 724 | tangeritin | 0.637558077 | B(iii) | 365.398 | 395.1119632 | POS | Flavonoids | 0.03 | 0.10 | 0.05 |
| 725 | rhoifolin | 0.635972923 | B(i) | 278.973 | 579.1819401 | POS | Flavonoids | 0.08 | 0.13 | 0.04 |
| 726 | ougenin | 0.635851308 | B(iii) | 416.77 | 317.1009002 | POS | Isoflavonoids | 0.41 | 0.64 | 0.13 |
| 727 | sophoracarpan a | 0.635587846 | B(iii) | 318.11 | 301.1060902 | POS | Isoflavonoids | 6.45 | 7.76 | 6.05 |
| 728 | pratol | 0.634597077 | B(iii) | 348.629 | 267.0656749 | NEG | Flavonoids | 0.58 | 1.49 | 0.08 |
| 729 | dehydrodeguelin,7a,13a-didehydrodeguelin | 0.634223538 | B(iii) | 386.831 | 393.1327224 | POS | Isoflavonoids | 0.21 | 0.20 | 0.13 |
| 730 | adenosine monophosphate (amp) | 0.633849154 | B(i) | 405.313 | 346.0557941 | NEG | Nucleosides | 0.06 | 0.04 | 0.04 |
| 731 | furostane base -2h + 1o, o-hex, o-pen-pen-dhex | 0.631884308 | B(iii) | 308.291 | 1003.503279 | POS | Steroids | 0.09 | 0.10 | 0.06 |
| 732 | baicalein | 0.631619077 | B(iii) | 500.525 | 269.0447182 | NEG | Flavonoids | 0.00 | 0.02 | 0.22 |
| 733 | benzyl alcohol + hex-hex | 0.631446692 | B(iii) | 522.498 | 431.1491434 | NEG | Others | 0.02 | 0.02 | 0.03 |
| 734 | homopisatin | 0.631314385 | B(iii) | 435.6985 | 301.1064292 | POS | Isoflavonoids | 1.77 | 1.98 | 1.34 |
| 735 | 2'-hydroxy-3,4,4',6'-tetramethoxychalcone | 0.630953615 | B(iii) | 414.947 | 345.1323351 | POS | Flavonoids | 0.20 | 0.13 | 0.17 |
| 736 | catalpol | 0.630748231 | B(i) | 193.347 | 361.113172 | NEG | Monoterpenoids | 0.01 | 0.00 | 0.00 |
| 737 | maximaflavanone a | 0.630072846 | B(iii) | 620.004 | 375.1930732 | POS | Flavonoids | 0.11 | 0.11 | 0.09 |
| 738 | khellin | 0.629848 | B(iii) | 311.9265 | 261.0749392 | POS | Chromanes | 0.05 | 0.19 | 0.11 |
| 739 | furostane base -2h + o-hex | 0.629343615 | B(iii) | 343.4915 | 577.3721575 | POS | Steroids | 0.02 | 0.06 | 0.04 |
| 740 | 3-hydroxyphenylacetic acid | 0.628853462 | B(iii) | 360.796 | 153.0543308 | POS | Phenolic acids | 1.85 | 8.90 | 0.38 |
| 741 | 2',3-dihydroxy-4,4',6'-trimethoxychalcone | 0.628367231 | B(iii) | 292.647 | 329.1025612 | NEG | Flavonoids | 0.05 | 0.07 | 0.02 |
| 742 | silychristin | 0.627549615 | B(iii) | 281.193 | 483.1258701 | POS | Lignans | 0.02 | 0.02 | 0.01 |
| 743 | 8-hydroxy-6-methoxy-7-[(2s,3r,4s,5s,6r)-3,4,5-trihydroxy-6-(hydroxymethyl)oxan-2-yl]oxychromen-2-one | 0.627431308 | B(iii) | 200.39 | 393.1158762 | POS | Coumarins | 0.09 | 0.10 | 2.11 |
| 744 | capsi-amide | 0.627155154 | B(iii) | 663.3255 | 270.2788419 | POS | Fatty Acids and Conjugates | 0.25 | 0.19 | 0.33 |
| 745 | 1-methoxyindole-3-carbaldehyde | 0.627056077 | B(iii) | 235.743 | 176.0703906 | POS | Tryptophan alkaloids | 0.04 | 0.08 | 0.02 |
| 746 | resorcinol | 0.626274308 | B(iii) | 418.281 | 109.0291908 | NEG | Phenolic acids | 0.01 | 0.00 | 0.01 |
| 747 | pseudo-anisatin | 0.626037615 | B(iii) | 364.476 | 337.1047559 | POS | Sesquiterpenoids | 0.15 | 0.23 | 0.06 |
| 748 | glyasperin c | 0.625759692 | B(iii) | 453.553 | 379.1535323 | POS | Isoflavonoids | 0.25 | 0.05 | 0.38 |
| 749 | cis-7-hexadecenoic acid | 0.625367462 | B(iii) | 637.036 | 253.2162724 | NEG | Fatty Acids and Conjugates | 1.52 | 1.02 | 1.13 |
| 750 | 1,3,5-trimethoxybenzene | 0.625262385 | B(iii) | 469.2 | 169.0855681 | POS | Phloroglucinols | 0.06 | 0.08 | 0.00 |
| 751 | (2~{r},3~{s},4~{s},5~{r},6~{s})-2-(hydroxymethyl)-6-[4-(3-hydroxypropyl)-2-methoxyphenoxy]oxane-3,4,5-triol | 0.624767923 | B(iii) | 265.959 | 362.1804014 | POS |  | 0.05 | 0.05 | 0.02 |
| 752 | pinostrobin | 0.624727923 | B(iii) | 289.579 | 271.0953771 | POS | Flavonoids | 0.07 | 0.08 | 0.08 |
| 753 | laxogenin | 0.624308385 | B(iii) | 597.64 | 431.314895 | POS | Steroids | 0.22 | 0.22 | 0.51 |
| 754 | dehydrodihydrorotenone | 0.623974 | B(iii) | 436.927 | 417.1323118 | POS | Isoflavonoids | 0.40 | 0.93 | 0.84 |
| 755 | (1s,8s,9s,10s,13r)-6,9,10-trimethyl-2-oxo-4,14-dioxatetracyclo[7.5.0.0,.0,tetradeca-3(7),5-dien-8-yl acetate | 0.623413385 | B(iii) | 397.8975 | 327.1218588 | POS |  | 0.10 | 0.11 | 2.36 |
| 756 | isosclerone | 0.622366846 | B(iii) | 440.6575 | 179.0699523 | POS |  | 0.19 | 0.41 | 0.10 |
| 757 | nitiducarpin | 0.621874 | B(iii) | 534.958 | 419.1845346 | POS | Isoflavonoids | 0.10 | 0.05 | 0.00 |
| 758 | cupressuflavone | 0.621697692 | B(iii) | 424.378 | 537.1897217 | POS | Flavonoids | 1.88 | 2.24 | 2.77 |
| 759 | dehydrovariabilin | 0.621560077 | B(iii) | 781.101 | 281.0807014 | NEG | Isoflavonoids | 0.01 | 0.00 | 0.01 |
| 760 | 1-(3,4-dimethoxyphenyl)-1,2-ethanediol 2-o-b-d-glucoside | 0.619815538 | B(iii) | 449.4685 | 361.1422634 | POS | Lignans | 0.63 | 0.16 | 0.20 |
| 761 | octadecanedioic acid | 0.619258 | B(iii) | 544.738 | 313.237219 | NEG | Fatty Acids and Conjugates | 0.16 | 0.20 | 0.52 |
| 762 | paulownin | 0.619065231 | B(iii) | 268.109 | 393.0947235 | POS | Lignans | 0.00 | 0.00 | 0.01 |
| 763 | (3,6,9-trimethylidene-2-oxo-3a,4,5,6a,7,8,9a,9b-octahydroazuleno[4,5-b]furan-8-yl) acetate | 0.618984538 | B(iii) | 351.313 | 289.1409134 | POS | Sesquiterpenoids | 0.07 | 0.07 | 0.07 |
| 764 | stictic acid | 0.618696923 | B(iii) | 332.308 | 387.0686822 | POS | Aromatic polyketides | 0.28 | 0.27 | 0.21 |
| 765 | arnicolide d | 0.618213308 | B(iii) | 316.31 | 333.1672472 | POS | Sesquiterpenoids | 0.21 | 0.23 | 0.22 |
| 766 | arabinose | 0.617532231 | B(iii) | 45.5697 | 149.0452049 | NEG | Saccharides | 0.03 | 0.03 | 0.02 |
| 767 | harman | 0.617398923 | B(iii) | 283.525 | 183.0913957 | POS | Tryptophan alkaloids | 0.05 | 0.10 | 0.02 |
| 768 | n~1~,n~4~-bis(3-aminopropyl)-1,4-butanediamine | 0.615734308 | B(iii) | 783.103 | 225.2066785 | POS | Ornithine alkaloids | 0.19 | 0.19 | 0.18 |
| 769 | l-rhamnose | 0.615455846 | B(i) | 45.365 | 209.0660907 | NEG | Saccharides | 0.01 | 0.02 | 0.02 |
| 770 | pinene oxide | 0.615293077 | B(iii) | 269.049 | 153.1270829 | POS |  | 0.03 | 0.07 | 0.03 |
| 771 | fisetinidol-4beta-ol 7,3',4'-trimethyl ether | 0.614769846 | B(iii) | 332.404 | 333.1328737 | POS | Flavonoids | 0.07 | 0.09 | 0.06 |
| 772 | 2,3-secoporrigenin | 0.613957077 | B(iii) | 317.333 | 461.2895804 | POS | Steroids | 0.03 | 0.05 | 0.08 |
| 773 | itaconate | 0.613888462 | B(iii) | 90.0107 | 129.018945 | NEG | Fatty Acids and Conjugates | 0.01 | 0.01 | 0.01 |
| 774 | 1'-acetoxyeugenol acetate | 0.613571154 | B(iii) | 375.9985 | 265.1060694 | POS | Phenylpropanoids | 0.22 | 0.26 | 0.30 |
| 775 | 9-(4-hydroxy-3,5-dimethoxyphenyl)-4-[3,4,5-trihydroxy-6-(hydroxymethyl)oxan-2-yl]oxy-5a,6,8a,9-tetrahydro-5h-[2]benzofuro[6,5-f][1,3]benzodioxol-8-one | 0.613201077 | B(iii) | 534.983 | 585.2032837 | POS | Lignans | 0.04 | 0.55 | 0.02 |
| 776 | 3,2',4'-trihydroxy-4-methoxychalcone | 0.613047538 | B(iii) | 589.54 | 287.0911194 | POS | Flavonoids | 0.31 | 0.22 | 0.72 |
| 777 | isopalmitic acid | 0.612786692 | B(iii) | 780.84 | 255.2322744 | NEG | Fatty Acids and Conjugates | 0.01 | 0.01 | 0.01 |
| 778 | isoamericanin a | 0.612679769 | B(iii) | 437.5565 | 329.1009561 | POS | Lignans | 0.02 | 0.10 | 0.02 |
| 779 | [(1s)-5-hydroxy-1-[(2s,3r,4s,5s,6r)-3,4,5-trihydroxy-6-(hydroxymethyl)oxan-2-yl]oxy-1,4a,5,7a-tetrahydrocyclopenta[c]pyran-7-yl]methyl benzoate | 0.612570923 | B(iii) | 315.418 | 451.158049 | POS | Monoterpenoids | 0.06 | 0.24 | 0.02 |
| 780 | gentiobiose | 0.612310538 | B(i) | 311.1855 | 387.1071849 | NEG | Saccharides | 0.00 | 0.01 | 0.01 |
| 781 | euparin | 0.612176077 | B(iii) | 297.903 | 234.111729 | POS | Coumarins | 0.06 | 0.05 | 0.03 |
| 782 | eupomatenoid 5 | 0.611409462 | B(iii) | 583.373 | 317.1140399 | POS | Lignans | 0.36 | 0.37 | 0.46 |
| 783 | verimol a | 0.611259 | B(iii) | 428.632 | 317.1378131 | POS | Phenolic acids | 10.41 | 3.61 | 0.26 |
| 784 | poncirin | 0.610478462 | B(i) | 281.7025 | 593.1874352 | NEG | Flavonoids | 0.00 | 0.00 | 0.00 |
| 785 | glucocerebrosides | 0.60958 | B(iii) | 774.368 | 714.550176 | POS | Spingolipids | 0.16 | 0.18 | 0.17 |
| 786 | (5s)-5-hydroxy-1,7-diphenylheptan-3-one | 0.609516231 | B(iii) | 243.128 | 321.1301846 | POS | Diarylheptanoids | 0.14 | 0.11 | 0.11 |
| 787 | austrobailignan 7 | 0.608194923 | B(iii) | 308.193 | 343.1526491 | POS | Lignans | 0.15 | 0.08 | 0.00 |
| 788 | confluentic acid | 0.607286846 | B(iii) | 382.1985 | 501.2610995 | POS | Aromatic polyketides | 0.16 | 0.52 | 0.15 |
| 789 | 2-methoxycinnamic acid | 0.606273077 | B(iii) | 286.802 | 177.0552962 | NEG | Phenylpropanoids | 0.01 | 0.01 | 0.00 |
| 790 | 2-[[(2r,3s)-2-(3,4-dihydroxyphenyl)-3,5-dihydroxy-3,4-dihydro-2h-chromen-7-yl]oxy]oxane-3,4,5-triol | 0.605260231 | B(iii) | 436.9035 | 271.1318858 | POS | Sesquiterpenoids | 1.06 | 0.71 | 0.41 |
| 791 | myrigalone h | 0.604935154 | B(iii) | 470.483 | 287.1275289 | POS | Flavonoids | 10.03 | 4.65 | 0.76 |
| 792 | 2-methoxyhomopterocarpin | 0.604811462 | B(iii) | 497.5695 | 315.1217815 | POS | Isoflavonoids | 0.33 | 1.22 | 0.15 |
| 793 | beta-vetivone | 0.603767615 | B(iii) | 569.731 | 219.1740323 | POS | Sesquiterpenoids | 0.10 | 0.09 | 0.06 |
| 794 | tigogenin | 0.603699385 | B(iii) | 670.627 | 417.3356789 | POS | Steroids | 0.29 | 0.13 | 0.54 |
| 795 | 3-(2-hydroxyphenyl)propanoate | 0.603596846 | B(iii) | 873.1915 | 165.0551963 | NEG | Others | 0.02 | 0.02 | 0.01 |
| 796 | gancaonin d | 0.602603077 | B(iii) | 374.3425 | 385.1273633 | POS | Isoflavonoids | 0.10 | 0.07 | 0.17 |
| 797 | mosloflavanone | 0.601956231 | B(iii) | 278.863 | 317.1018518 | POS | Flavonoids | 0.39 | 0.18 | 0.10 |
| 798 | (r,e)-8,8-dimethyl-2-oxo-2,8,9,10-tetrahydropyrano[2,3-f]chromen-9-yl 2-methylbut-2-enoate | 0.601873077 | B(iii) | 467.261 | 329.1380421 | POS | Coumarins | 0.15 | 0.25 | 0.14 |
| 799 | hydroxyprogesterone caproate | 0.600669538 | B(iii) | 519.147 | 429.2998791 | POS | Steroids | 0.27 | 0.60 | 0.77 |
| 800 | tanshinone iia | 0.599561154 | B(iii) | 215.0075 | 293.1237882 | NEG | Diterpenoids | 0.01 | 0.01 | 0.00 |
| 801 | samaderin a | 0.597825385 | B(iii) | 360.128 | 331.1165219 | POS | Triterpenoids | 0.23 | 0.27 | 0.18 |
| 802 | 2'-hydroxy-2,3,4',6'-tetramethoxychalcone | 0.597797538 | B(iii) | 476.3925 | 345.1325068 | POS | Flavonoids | 0.08 | 0.04 | 0.28 |
| 803 | 5-hydroxy-8,8-dimethyl-6-(2-methylpropanoyl)-4-phenylpyrano[2,3-h]chromen-2-one | 0.597202231 | B(iii) | 513.2 | 391.1538373 | POS |  | 0.29 | 0.09 | 0.39 |
| 804 | visnagin | 0.596639846 | B(iii) | 165.5835 | 253.0470342 | POS | Chromanes | 0.03 | 0.04 | 0.05 |
| 805 | suberosin | 0.594588538 | B(iii) | 486.946 | 267.1008316 | POS | Coumarins | 0.11 | 0.04 | 0.09 |
| 806 | 7-dehydrocholesterol | 0.593554 | B(iii) | 756.217 | 385.3470822 | POS | Steroids | 0.03 | 0.04 | 0.07 |
| 807 | sorbitol | 0.593233846 | B(iii) | 44.27185 | 221.0416136 | POS | Fatty acyls | 1.34 | 2.41 | 1.96 |
| 808 | 2'-hydroxy-3',4',6',3,4-pentamethoxychalcone | 0.592281385 | B(iii) | 335.7045 | 375.1428784 | POS | Flavonoids | 0.30 | 0.21 | 0.77 |
| 809 | cucurbitacin p | 0.590420462 | B(iii) | 283.2785 | 521.3354113 | POS | Triterpenoids | 0.07 | 0.01 | 0.01 |
| 810 | isosakuranetin | 0.590222846 | B(iii) | 263.5645 | 285.0763067 | NEG | Flavonoids | 0.03 | 0.02 | 0.11 |
| 811 | scutellarein 4'-methyl ether | 0.589590538 | B(iii) | 325.626 | 299.0555811 | NEG | Flavonoids | 0.03 | 0.11 | 0.02 |
| 812 | 2,6-dimethoxyquinone | 0.588284 | B(iii) | 219.383 | 169.0491552 | POS | Aromatic polyketides | 0.01 | 0.03 | 0.03 |
| 813 | epicatechin 5,3'-dimethyl ether | 0.586962231 | B(iii) | 367.905 | 319.1167487 | POS | Flavonoids | 0.66 | 0.80 | 0.77 |
| 814 | rhamnazin 3-rutinoside | 0.585526462 | B(iii) | 343.494 | 639.1835582 | POS | Flavonoids | 0.05 | 0.07 | 0.18 |
| 815 | capsaicin | 0.585137692 | B(iii) | 49.725 | 344.1544568 | POS |  | 2.45 | 2.04 | 3.94 |
| 816 | enol-3-ethyl-1,2-cyclopentanedione | 0.584472077 | B(iii) | 32.21105 | 127.0750513 | POS | Others | 0.25 | 0.19 | 0.15 |
| 817 | pachyrrhizin | 0.583913846 | B(iii) | 50.7823 | 354.1024059 | POS | Coumarins | 0.06 | 0.04 | 0.12 |
| 818 | camellenodiol | 0.583344077 | B(iii) | 683.5555 | 443.3511298 | POS | Triterpenoids | 0.12 | 0.13 | 0.07 |
| 819 | farnesoic acid | 0.582244538 | B(iii) | 482.924 | 237.1846196 | POS | Sesquiterpenoids | 0.72 | 0.15 | 0.24 |
| 820 | 6'-methoxy-4,2',4',beta-tetrahydroxy-alpha,beta-dihydrochalcone | 0.582144077 | B(iii) | 543.903 | 327.0770625 | POS | Flavonoids | 0.03 | 0.06 | 0.05 |
| 821 | dalbergione, 4-methoxy-4'-hydroxy- | 0.581760154 | B(iii) | 445.599 | 269.0812932 | NEG | Flavonoids | 0.21 | 0.25 | 0.74 |
| 822 | chalcone base + 3o, 1meo, 1prenyl | 0.581184154 | B(iii) | 534.914 | 353.1380804 | NEG | Flavonoids | 0.00 | 0.02 | 0.00 |
| 823 | scropolioside d | 0.580650308 | B(iii) | 361.479 | 721.2283263 | NEG | Monoterpenoids | 0.01 | 0.02 | 0.00 |
| 824 | daidzein 7-o-apiosyl-(1->6)-glucoside | 0.580363231 | B(iii) | 305.8715 | 549.1737956 | POS | Isoflavonoids | 0.08 | 0.04 | 0.01 |
| 825 | estrone | 0.580284154 | B(iii) | 519.092 | 253.1947246 | POS | Steroids | 0.08 | 0.13 | 0.27 |
| 826 | cis-reticulatacin-10-one | 0.580057154 | B(iii) | 727.639 | 607.4938977 | POS | Linear polyketides | 0.05 | 0.02 | 0.02 |
| 827 | 13-oxoode | 0.579328154 | B(iii) | 514.819 | 293.2112919 | NEG | Octadecanoids | 0.03 | 0.02 | 0.02 |
| 828 | matairesinol | 0.578583923 | B(iii) | 304.475 | 359.1478384 | POS | Lignans | 0.20 | 0.17 | 0.07 |
| 829 | garcilivin b | 0.578359385 | B(iii) | 380.939 | 621.1734309 | POS | Xanthones | 0.05 | 0.04 | 0.14 |
| 830 | isoeugenitol | 0.578208308 | B(iii) | 305.298 | 205.050001 | NEG | Chromanes | 0.01 | 0.01 | 0.02 |
| 831 | (4s,5z,6s)-5-[2-[(e)-3-(4-hydroxyphenyl)prop-2-enoyl]oxyethylidene]-4-(2-methoxy-2-oxoethyl)-6-[(2s,3r,4s,5s,6r)-3,4,5-trihydroxy-6-(hydroxymethyl)oxan-2-yl]oxy-4h-pyran-3-carboxylic acid | 0.578100231 | B(iii) | 652.4335 | 567.1640644 | POS | Monoterpenoids | 0.08 | 0.16 | 0.10 |
| 832 | thiamine | 0.577764154 | B(i) | 327.277 | 265.1066457 | POS | Others | 0.09 | 0.02 | 0.05 |
| 833 | [(1s,3ar,5r,5ar,8ar,9s,9ar)-1,5,8a-trimethyl-2,8-dioxo-3a,4,5,5a,9,9a-hexahydro-1h-azuleno[6,5-b]furan-9-yl] (z)-2-methylbut-2-enoate | 0.577313923 | B(iii) | 367.148 | 385.1988532 | POS | Sesquiterpenoids | 0.21 | 0.22 | 0.89 |
| 834 | 5,3'-dihydroxy-7,2'-dimethoxyflavanone | 0.577221 | B(iii) | 338.218 | 317.1015271 | POS | Flavonoids | 0.43 | 1.04 | 0.25 |
| 835 | puerarin | 0.576538308 | B(i) | 282.417 | 415.1028139 | NEG | Isoflavonoids | 0.01 | 0.00 | 0.00 |
| 836 | tutin | 0.575949538 | B(iii) | 229.7795 | 277.1046779 | POS | Sesquiterpenoids | 0.26 | 0.22 | 0.26 |
| 837 | 2,4,4'-trihydroxydihydrochalcone | 0.575646615 | B(iii) | 398.509 | 259.0959366 | POS | Flavonoids | 0.99 | 0.44 | 0.75 |
| 838 | [8-[2-(3-methylbutanoyloxy)propan-2-yl]-2-oxo-8,9-dihydrofuro[2,3-h]chromen-9-yl] 3-methylbutanoate | 0.575481846 | B(iii) | 467.255 | 453.1905381 | POS | Coumarins | 0.26 | 0.50 | 0.04 |
| 839 | [1a-(hydroxymethyl)-2-[3,4,5-trihydroxy-6-(hydroxymethyl)oxan-2-yl]oxy-2,5a,6,6a-tetrahydro-1bh-oxireno[5,6]cyclopenta[1,3-c]pyran-6-yl] 3,4-dimethoxybenzoate | 0.575259 | B(iii) | 448.119 | 544.2039344 | POS | Monoterpenoids | 1.20 | 1.33 | 0.86 |
| 840 | dalpaniculin | 0.574830154 | B(iii) | 350 | 537.1509665 | POS | Others | 1.55 | 0.83 | 0.85 |
| 841 | scopoloside ii | 0.574072385 | B(iii) | 340.2095 | 771.4147018 | POS | Steroids | 0.17 | 0.19 | 0.20 |
| 842 | phenylacetic acid + 2o, o-hex | 0.573954769 | B(iii) | 245.634 | 329.0866888 | NEG | Phenolic acids | 0.01 | 0.00 | 0.01 |
| 843 | prenyletin | 0.573441769 | B(iii) | 289.7075 | 245.0816243 | NEG | Coumarins | 0.02 | 0.01 | 0.00 |
| 844 | salsolinol | 0.573126692 | B(iii) | 151.955 | 180.1012942 | POS | Tyrosine alkaloids | 0.12 | 0.16 | 0.03 |
| 845 | lomatin | 0.572979615 | B(iii) | 367.837 | 247.0960432 | POS | Coumarins | 0.12 | 0.39 | 0.08 |
| 846 | hexadecanedioic acid | 0.572362769 | B(iii) | 494.3115 | 285.2064995 | NEG | Fatty Acids and Conjugates | 0.05 | 0.11 | 0.38 |
| 847 | 3,5-dihydroxy-2-(4-hydroxyphenyl)-7-[3,4,5-trihydroxy-6-(hydroxymethyl)oxan-2-yl]oxy-2,3-dihydrochromen-4-one | 0.572243308 | B(iii) | 258.78 | 451.1583785 | POS | Flavonoids | 0.04 | 0.01 | 0.01 |
| 848 | schizandrin | 0.572064231 | B(iii) | 80.0688 | 471.1701163 | POS | Lignans | 0.00 | 0.00 | 0.01 |
| 849 | (2~{r},3~{s},4~{s},5~{r},6~{r})-2-[[(2~{r},3~{r},4~{r})-3,4-dihydroxy-4-(hydroxymethyl)oxolan-2-yl]oxymethyl]-6-phenylmethoxyoxane-3,4,5-triol | 0.571854462 | B(iii) | 260.219 | 420.1853431 | POS |  | 0.02 | 0.22 | 0.01 |
| 850 | 2'-o-methylisoliquiritigenin | 0.571239923 | B(iii) | 328.317 | 271.0955328 | POS | Flavonoids | 20.41 | 10.91 | 29.56 |
| 851 | cearoin | 0.571214923 | B(iii) | 204.958 | 243.0659977 | NEG | Phloroglucinols | 0.01 | 0.00 | 0.00 |
| 852 | methyl 4-hydroxyphenylacetate | 0.570997538 | B(iii) | 351.9005 | 165.0552022 | NEG | Phenolic acids | 0.03 | 0.03 | 0.04 |
| 853 | 3,4,5-trimethoxycinnamic aldehyde | 0.570801308 | B(iii) | 399.806 | 223.0956781 | POS | Phenylpropanoids | 0.03 | 0.01 | 0.60 |
| 854 | 5,7,8-trimethoxyflavanone | 0.569890308 | B(iii) | 585.992 | 315.1215849 | POS | Flavonoids | 0.02 | 0.06 | 0.02 |
| 855 | fa 18:1+3o | 0.568999 | B(iii) | 410.423 | 329.2327505 | NEG | Octadecanoids | 0.03 | 0.03 | 0.04 |
| 856 | 2'-hydroxydaidzein | 0.568963846 | B(iii) | 388.727 | 271.0594949 | POS | Isoflavonoids | 10.54 | 21.75 | 2.53 |
| 857 | physalin f | 0.568330615 | B(iii) | 317.45 | 527.1898865 | POS | Steroids | 0.06 | 0.03 | 0.01 |
| 858 | vitexin 2''-glucoside | 0.567916462 | B(i) | 423.9315 | 595.1706673 | POS |  | 0.40 | 0.34 | 0.44 |
| 859 | delta9-tetrahydrocannabinol | 0.567641308 | B(iii) | 675.773 | 315.2307656 | POS | Meroterpenoids | 0.47 | 0.59 | 0.42 |
| 860 | 3-(2,4-dihydroxyphenyl)-7-hydroxy-6,8-bis(3-methylbut-2-enyl)-2,3-dihydrochromen-4-one | 0.566484385 | B(iii) | 273.531 | 407.1919457 | NEG | Isoflavonoids | 0.00 | 0.00 | 0.01 |
| 861 | 2-furanylmethyl butanoate | 0.565105615 | B(iii) | 298.671 | 169.0756739 | POS | Fatty esters | 0.02 | 0.02 | 0.03 |
| 862 | dibutyl phthalate | 0.563367308 | B(iii) | 583.337 | 279.1585783 | POS | Phenolic acids | 0.14 | 0.12 | 0.19 |
| 863 | 5,7-dihydroxy-2-(3-hydroxy-4-methoxyphenyl)-2,3-dihydrochromen-4-one | 0.563251077 | B(iii) | 223.006 | 301.0713542 | NEG | Flavonoids | 0.00 | 0.00 | 0.00 |
| 864 | stepharine | 0.562947538 | B(iii) | 412.075 | 298.1427969 | POS | Tyrosine alkaloids | 0.24 | 0.56 | 0.02 |
| 865 | nortrachelogenin | 0.562689 | B(iii) | 46.6286 | 413.096737 | POS | Lignans | 0.02 | 0.03 | 0.01 |
| 866 | secologanin | 0.562329154 | B(iii) | 477.693 | 389.1372366 | POS | Monoterpenoids | 0.13 | 0.14 | 0.07 |
| 867 | sesamin | 0.562049231 | B(iii) | 346.109 | 355.1166114 | POS | Lignans | 0.42 | 0.37 | 0.18 |
| 868 | galactosamine | 0.561293308 | B(iii) | 44.2895 | 180.0861033 | POS | Aminosugars and aminoglycosides | 0.36 | 0.25 | 0.30 |
| 869 | 2-(2-oxo-8,9-dihydrofuro[2,3-h]chromen-8-yl)propan-2-yl acetate | 0.560483923 | B(iii) | 298.56 | 327.0628716 | POS | Coumarins | 0.38 | 0.25 | 0.53 |
| 870 | hispidulin | 0.560246538 | B(iii) | 395.2355 | 301.070407 | POS | Flavonoids | 2.47 | 0.76 | 1.13 |
| 871 | lucuminic acid | 0.559696615 | B(iii) | 389.3555 | 447.1435535 | POS |  | 0.38 | 0.95 | 0.72 |
| 872 | [(2s,3r,4s,5s,6r)-3,4,5-trihydroxy-6-(hydroxymethyl)oxan-2-yl] (2e,6e)-8-hydroxy-2,6-dimethylocta-2,6-dienoate | 0.558270154 | B(iii) | 261.335 | 345.1549915 | NEG | Monoterpenoids | 0.00 | 0.00 | 0.00 |
| 873 | hematoxylin | 0.557293923 | B(iii) | 311.0875 | 341.041964 | POS |  | 0.02 | 0.34 | 0.03 |
| 874 | n-acetyl-dl-glutamic acid | 0.556829231 | B(iii) | 47.2288 | 190.0706018 | POS | Fatty Acids and Conjugates | 0.16 | 0.02 | 0.03 |
| 875 | lycorine | 0.556802615 | B(iii) | 783.474 | 288.1303075 | POS | Lysine alkaloids | 0.09 | 0.05 | 0.02 |
| 876 | (+)-dihydrokavain | 0.556714846 | B(iii) | 384.875 | 255.1005899 | POS | Styrylpyrones | 1.56 | 0.54 | 0.94 |
| 877 | pseudoionone | 0.556659923 | B(iii) | 436.494 | 193.1581575 | POS | Apocarotenoids | 0.07 | 0.04 | 0.05 |
| 878 | 1,5-dihydroxy-2,3-dimethoxy-10-methylacridin-9-one | 0.556547308 | B(iii) | 435.501 | 302.1095232 | POS |  | 0.28 | 0.38 | 0.23 |
| 879 | benzoic acid | 0.554828769 | B(iii) | 782.428 | 121.0292768 | NEG | Phenolic acids | 0.06 | 0.04 | 0.03 |
| 880 | epicalyxin j | 0.553873308 | B(iii) | 517.1775 | 687.2594307 | POS | Diarylheptanoids | 0.24 | 0.51 | 0.03 |
| 881 | 2-hydroxyquinoline | 0.553042692 | B(iii) | 334.694 | 144.0450492 | NEG | Anthranilic acid alkaloids | 0.01 | 0.01 | 0.00 |
| 882 | (s,s)-asperphenamate | 0.552709385 | B(iii) | 530.506 | 529.2090872 | POS | Flavonoids | 0.11 | 0.07 | 0.03 |
| 883 | 5,7-dimethoxy-6-c-methylflavone | 0.551876077 | B(iii) | 522.311 | 297.1114187 | POS | Flavonoids | 0.45 | 0.58 | 0.07 |
| 884 | 7-methylxanthine | 0.551126308 | B(i) | 41.4561 | 165.0399615 | NEG | Pseudoalkaloids | 0.01 | 0.02 | 0.01 |
| 885 | 3-(4-hydroxy-2-methoxyphenyl)-2-propenal | 0.551088077 | B(iii) | 324.623 | 179.0697178 | POS | Phenylpropanoids | 0.08 | 0.05 | 0.08 |
| 886 | liquiritin apioside | 0.550613615 | B(iii) | 390.089 | 551.1672668 | POS | Flavonoids | 1.15 | 1.27 | 0.90 |
| 887 | norephedrine | 0.550101846 | B(iii) | 594.294 | 152.1067908 | POS | Pseudoalkaloids | 0.04 | 0.02 | 0.02 |
| 888 | ergostane-3,6-dione | 0.549861538 | B(iii) | 754.676 | 415.3568055 | POS | Steroids | 0.92 | 0.71 | 0.27 |
| 889 | oleuropein aglycon derivative | 0.549483692 | B(iii) | 294.117 | 377.1240392 | NEG | Monoterpenoids | 0.01 | 0.00 | 0.01 |
| 890 | d-(+)-cellotriose | 0.549201615 | B(iii) | 48.9548 | 503.1611241 | NEG | Saccharides | 0.02 | 0.03 | 0.02 |
| 891 | muzanzagenin | 0.547472385 | B(iii) | 401.6775 | 443.2785724 | POS | Steroids | 0.22 | 0.35 | 0.17 |
| 892 | fa 18:2+1o | 0.547170692 | B(iii) | 518.756 | 295.2274828 | NEG | Octadecanoids | 0.05 | 0.12 | 0.02 |
| 893 | methyl 2-hydroxy-6-(4-hydroxy-2-methoxy-6-methoxycarbonylphenoxy)-4-methylbenzoate | 0.547149769 | B(iii) | 284.649 | 363.1069908 | POS |  | 0.02 | 0.02 | 0.05 |
| 894 | 2-pyrrolidineacetic acid | 0.546924231 | B(iii) | 198.806 | 130.0858669 | POS | Lysine alkaloids | 0.01 | 0.01 | 0.08 |
| 895 | demethoxycentaureidin 7-o-rutinoside | 0.546596846 | B(iii) | 327.392 | 637.1903746 | NEG | Flavonoids | 0.02 | 0.00 | 0.00 |
| 896 | acetylcholine | 0.544602692 | B(iii) | 46.1753 | 146.1171614 | POS |  | 0.08 | 0.13 | 0.04 |
| 897 | (2z,2'z)-(3r,4r)-2,2-dimethyl-8-oxo-2,3,4,8-tetrahydropyrano[3,2-g]chromene-3,4-diyl bis(2-methylbut-2-enoate) | 0.543386231 | B(iii) | 373.918 | 449.1586598 | POS | Coumarins | 0.15 | 0.26 | 0.15 |
| 898 | 6''-p-coumaroylprunin | 0.543107308 | B(iii) | 378.3375 | 581.157127 | POS | Flavonoids | 0.42 | 0.24 | 0.22 |
| 899 | histamine | 0.542978615 | B(iii) | 769.213 | 112.0865945 | POS | Histidine alkaloids | 0.54 | 0.32 | 0.79 |
| 900 | cytidine | 0.542528615 | B(iii) | 214.614 | 244.0937991 | POS | Nucleosides | 0.05 | 0.05 | 0.05 |
| 901 | isoamylamine | 0.542171154 | B(i) | 203.841 | 88.11191508 | POS |  | 0.02 | 0.02 | 0.01 |
| 902 | isoliquiritin | 0.541958385 | B(iii) | 487.1865 | 441.1097066 | POS | Flavonoids | 0.36 | 0.05 | 0.06 |
| 903 | menthol(-) | 0.541482923 | B(iii) | 349.886 | 174.1848195 | POS | Monoterpenoids | 0.07 | 0.06 | 0.07 |
| 904 | spirostane-3,6-dione | 0.539817308 | B(iii) | 397.25 | 429.2996188 | POS | Steroids | 0.13 | 0.28 | 0.32 |
| 905 | tomatine | 0.539602231 | B(iii) | 382.9675 | 1034.550192 | POS | Pseudoalkaloids | 0.15 | 0.38 | 0.01 |
| 906 | avenanthramide c | 0.539289538 | B(iii) | 288.338 | 316.0873508 | POS |  | 0.03 | 0.03 | 0.03 |
| 907 | 13-hode-[d4] | 0.538506231 | B(iii) | 624.038 | 299.2589313 | NEG | Octadecanoids | 0.02 | 0.02 | 0.01 |
| 908 | dodecanamide | 0.538304385 | B(iii) | 527.742 | 200.2003851 | POS | Fatty Acids and Conjugates | 1.52 | 1.10 | 2.45 |
| 909 | batatasin iii | 0.537925846 | B(iii) | 542.296 | 267.1009277 | POS | Stilbenoids | 0.05 | 0.02 | 0.03 |
| 910 | leonoside b | 0.535726462 | B(iii) | 419.254 | 785.2924158 | POS | Phenylethanoids | 0.22 | 0.71 | 0.23 |
| 911 | 2',4'-dihydroxy-2''-(1-hydroxy-1-methylethyl)dihydrofuro[2,3-h]flavanone | 0.535530769 | B(iii) | 413.503 | 357.1322062 | POS | Flavonoids | 0.15 | 0.11 | 0.07 |
| 912 | thonningine b | 0.533721615 | B(iii) | 366.507 | 409.1273001 | POS | Coumarins | 0.08 | 0.21 | 0.08 |
| 913 | mukoenine a | 0.533389308 | B(iii) | 436.4885 | 266.1508701 | POS | Tryptophan alkaloids | 1.84 | 1.48 | 2.34 |
| 914 | 1,2-bis-o-sinapoyl-beta-d-glucoside | 0.533127 | B(iii) | 359.282 | 593.1783494 | POS | Phenylpropanoids | 0.07 | 0.09 | 0.10 |
| 915 | 5,7-dioxa-12-azapentacyclo[10.6.1.0^{2,10}.0^{4,8}.0^{13,18}]nonadeca-2,4(8),9,17-tetraene-15,16-diol | 0.532964308 | B(iii) | 470.484 | 288.1306864 | POS |  | 1.80 | 0.82 | 0.13 |
| 916 | glycyrrhetinic acid | 0.532770692 | B(iii) | 616.117 | 469.3304085 | NEG | Triterpenoids | 0.01 | 0.00 | 0.00 |
| 917 | momordenol | 0.532707 | B(iii) | 769.158 | 427.3570144 | POS | Steroids | 0.68 | 0.77 | 0.39 |
| 918 | soyasapogenol a | 0.532569846 | B(iii) | 724.3715 | 475.3775493 | POS | Triterpenoids | 0.03 | 0.03 | 0.02 |
| 919 | moghanin a | 0.532491 | B(iii) | 344.135 | 509.1214607 | POS | Flavonoids | 10.41 | 5.12 | 1.89 |
| 920 | 2',5'-dihydroxy-4-methoxychalcone | 0.531470538 | B(iii) | 379.37 | 269.0811295 | NEG | Flavonoids | 21.43 | 6.42 | 12.85 |
| 921 | (2s,3s)-2-(3,4,5-trihydroxyphenyl)-3,4-dihydro-2h-chromene-3,5,7-triol | 0.531420923 | B(iii) | 293.519 | 305.0664706 | NEG | Flavonoids | 0.00 | 0.00 | 0.00 |
| 922 | combrestatin a4 | 0.529729615 | B(iii) | 806.022 | 317.1374337 | POS | Stilbenoids | 0.27 | 0.60 | 0.10 |
| 923 | garciduol b | 0.528290077 | B(iii) | 397.896 | 503.109044 | POS | Phloroglucinols | 0.12 | 0.48 | 0.06 |
| 924 | stypandrol | 0.527954462 | B(iii) | 367.802 | 431.1455004 | POS | Naphthalenes | 0.22 | 0.28 | 0.18 |
| 925 | apocynin | 0.527202615 | B(iii) | 464.644 | 167.0698206 | POS | Phenolic acids | 2.20 | 1.77 | 0.21 |
| 926 | [3,4,5-trihydroxy-6-(hydroxymethyl)oxan-2-yl] 3,4,5-trimethoxybenzoate | 0.525450308 | B(iii) | 463.964 | 392.1565285 | POS |  | 0.09 | 0.04 | 0.14 |
| 927 | culantraramine | 0.525390846 | B(iii) | 626.557 | 491.36076 | POS |  | 0.14 | 0.10 | 0.16 |
| 928 | 5-isopropyl-2-methylphenol acetate | 0.524902846 | B(iii) | 315.22 | 193.1217453 | POS | Monoterpenoids | 0.08 | 0.06 | 0.03 |
| 929 | polyphyllin a | 0.523517692 | B(iii) | 519.092 | 577.3728465 | POS | Steroids | 0.03 | 0.05 | 0.36 |
| 930 | osmanthuside h | 0.523235615 | B(iii) | 221.247 | 431.1595982 | NEG | Phenylethanoids | 0.00 | 0.00 | 0.00 |
| 931 | dioctyl hexanedioate | 0.523224154 | B(iii) | 724.3345 | 371.3153366 | POS | Fatty esters | 0.02 | 0.01 | 0.02 |
| 932 | 1,2-dihydroxyheptadec-16-en-4-yl acetate | 0.522298538 | B(iii) | 628.553 | 351.2518375 | POS | Fatty acyls | 0.20 | 0.00 | 0.00 |
| 933 | 2-phenylethanol | 0.521787308 | B(iii) | 237.78 | 105.0695468 | POS | Phenylethanoids | 0.02 | 0.02 | 0.00 |
| 934 | lapidin | 0.520996615 | B(iii) | 439.091 | 335.2182224 | POS | Sesquiterpenoids | 0.04 | 0.04 | 0.16 |
| 935 | (1~{s},4~{a}~{s},6~{s},7~{r},7~{a}~{s})-6-[(~{e})-3-(4-hydroxyphenyl)prop-2-enoyl]oxy-7-methyl-1-[(2~{s},3~{r},4~{s},5~{s},6~{r})-3,4,5-trihydroxy-6-(hydroxymethyl)oxan-2-yl]oxy-1,4~{a},5,6,7,7~{a}-hexahydrocyclopenta[c]pyran-4-carboxylic acid | 0.520857846 | B(iii) | 406.386 | 523.1736976 | POS |  | 5.10 | 7.07 | 4.93 |
| 936 | glyasperin d | 0.520605462 | B(iii) | 522.906 | 393.1691404 | POS | Isoflavonoids | 6.24 | 0.95 | 0.05 |
| 937 | villol | 0.520269923 | B(iii) | 205.209 | 465.1224285 | POS | Isoflavonoids | 0.02 | 0.02 | 0.15 |
| 938 | luvangetin | 0.520148231 | B(iii) | 455.408 | 281.0781809 | POS | Coumarins | 0.07 | 0.08 | 0.12 |
| 939 | rhodomyrtoxin b | 0.519991923 | B(iii) | 221.289 | 427.1808234 | NEG | Phloroglucinols | 0.00 | 0.00 | 0.01 |
| 940 | 5,6,2'-trimethoxyflavone | 0.519848385 | B(iii) | 48.7057 | 351.067967 | POS | Flavonoids | 0.20 | 0.29 | 0.17 |
| 941 | 3,4-dimethyl-2,5-bis(3,4,5-trimethoxyphenyl)oxolane | 0.519529923 | B(iii) | 385.5325 | 471.1799286 | POS | Lignans | 0.20 | 0.07 | 0.03 |
| 942 | licarin a | 0.519355769 | B(iii) | 374.7015 | 325.1070772 | NEG | Lignans | 0.03 | 0.01 | 0.13 |
| 943 | s-2-propenyl 1-propenesulfinothioate | 0.518437615 | B(iii) | 40.99175 | 163.0266429 | POS | Others | 0.06 | 0.04 | 0.05 |
| 944 | dipropyl phthalate | 0.517845077 | B(iii) | 473.3555 | 249.1124055 | NEG | Phenolic acids | 0.01 | 0.03 | 0.02 |
| 945 | (1r,3r,4s,5r)-1,3,4-trihydroxy-5-[(e)-3-(4-hydroxy-3-methoxyphenyl)prop-2-enoyl]oxycyclohexane-1-carboxylic acid | 0.516742538 | B(iii) | 258.0565 | 369.1146925 | POS | Phenylpropanoids | 0.05 | 0.03 | 0.02 |
| 946 | adipic acid | 0.516641538 | B(iii) | 44.3274 | 147.0648056 | POS | Fatty Acids and Conjugates | 0.18 | 0.41 | 0.41 |
| 947 | sanguinarine | 0.516439154 | B(iii) | 206.5595 | 333.0949615 | POS | Others | 0.02 | 0.02 | 0.02 |
| 948 | euchrenone a7 | 0.516187308 | B(iii) | 301.65 | 341.1369391 | POS | Flavonoids | 0.08 | 0.06 | 0.06 |
| 949 | haploside c | 0.516121308 | B(iii) | 359.498 | 697.188472 | POS | Flavonoids | 0.03 | 0.02 | 0.05 |
| 950 | 8'-apozeaxanthinal | 0.515881231 | B(iii) | 679.422 | 432.2979812 | POS | Apocarotenoids | 0.11 | 0.02 | 0.00 |
| 951 | melongoside h | 0.514627385 | B(iii) | 340.842 | 885.4810172 | POS | Steroids | 0.19 | 0.20 | 0.11 |
| 952 | trans-12-hydroxyjasmonic acid | 0.514271077 | B(iii) | 267.678 | 227.1270187 | POS |  | 0.04 | 0.04 | 0.01 |
| 953 | rubiadin | 0.512558308 | B(iii) | 374.335 | 272.0993544 | POS | Polycyclic aromatic polyketides | 6.00 | 2.21 | 6.59 |
| 954 | butyl paraben | 0.512398 | B(iii) | 382.99 | 195.1009565 | POS | Phenolic acids | 0.14 | 0.27 | 0.01 |
| 955 | 8-epideoxyloganin | 0.511644692 | B(iii) | 546.627 | 375.1692957 | POS | Monoterpenoids | 0.06 | 0.09 | 0.24 |
| 956 | chavicol | 0.511092769 | B(iii) | 594.294 | 135.0801018 | POS | Phenylpropanoids | 0.11 | 0.04 | 0.04 |
| 957 | sempervirenoside b | 0.510945231 | B(iii) | 543.751 | 851.3062655 | POS | Flavonoids | 0.15 | 0.16 | 0.00 |
| 958 | caftaric acid | 0.510890692 | B(iii) | 291.213 | 313.0463324 | POS | Phenylpropanoids | 0.18 | 0.51 | 0.45 |
| 959 | cratoxyarborenone e | 0.510525769 | B(iii) | 495.414 | 433.1635459 | POS | Xanthones | 0.11 | 0.16 | 0.06 |
| 960 | corchorifatty acid f | 0.509382538 | B(iii) | 366.841 | 329.2312302 | POS | Octadecanoids | 0.05 | 0.06 | 0.21 |
| 961 | andrachcinidine | 0.509202769 | B(iii) | 670.306 | 228.1953648 | POS | Lysine alkaloids | 1.54 | 1.13 | 1.51 |
| 962 | 3,4,5-trimethoxycinnamic acid | 0.508953692 | B(iii) | 272.985 | 237.0764696 | NEG | Phenylpropanoids | 0.00 | 0.00 | 0.00 |
| 963 | 5-o-methyllicoricidin | 0.508308154 | B(iii) | 613.5205 | 437.2319235 | NEG | Isoflavonoids | 0.01 | 0.01 | 0.00 |
| 964 | kushenol f | 0.507196 | B(iii) | 523.087 | 447.1784647 | POS | Flavonoids | 0.06 | 0.15 | 0.02 |
| 965 | 2-[3-[2-(1,3-benzodioxol-5-yl)-7-methoxy-1-benzofuran-5-yl]-3-hydroxypropoxy]-6-(hydroxymethyl)oxane-3,4,5-triol | 0.50637 | B(iii) | 48.7057 | 522.2011582 | POS | Lignans | 0.26 | 0.30 | 0.28 |
| 966 | 5,7-dihydroxy-3-(4-hydroxyphenyl)-6,8-bis[3,4,5-trihydroxy-6-(hydroxymethyl)oxan-2-yl]chromen-4-one | 0.506183 | B(iii) | 393.817 | 595.1724241 | POS |  | 0.11 | 0.02 | 0.04 |
| 967 | glutamylphenylalanine | 0.505292231 | B(iii) | 202.9565 | 293.1238612 | NEG | Small peptides | 0.01 | 0.01 | 0.01 |
| 968 | betulafolienetriol | 0.504882308 | B(iii) | 757.0235 | 461.397405 | POS | Triterpenoids | 0.03 | 0.02 | 0.01 |
| 969 | gingerdione | 0.504677231 | B(iii) | 521.79 | 293.1742162 | POS | Others | 0.21 | 0.01 | 0.01 |
| 970 | polygalic acid | 0.504251154 | B(iii) | 678.465 | 489.3207955 | POS | Triterpenoids | 0.10 | 0.24 | 0.20 |
| 971 | ethyl acetate | 0.504224538 | B(iii) | 330.9705 | 89.05947235 | POS | Fatty esters | 0.01 | 0.00 | 0.01 |
| 972 | anthothecol | 0.504118769 | B(iii) | 374.3175 | 481.2349217 | POS | Triterpenoids | 0.27 | 0.39 | 0.04 |
| 973 | 3-[(2s,3r,4s,5r,6r)-3,5-dihydroxy-6-(hydroxymethyl)-4-[(2s,3r,4r,5r,6s)-3,4,5-trihydroxy-6-methyloxan-2-yl]oxyoxan-2-yl]oxy-5,7-dihydroxy-2-(4-hydroxyphenyl)chromen-4-one | 0.503942231 | B(iii) | 286.1215 | 633.1200468 | POS | Flavonoids | 0.02 | 0.02 | 0.00 |
| 974 | 5-{8(z),11(z)-pentadecadienyl}resorcinol | 0.503843923 | B(iii) | 574.131 | 339.2311887 | POS | Aromatic polyketides | 0.05 | 0.04 | 0.03 |
| 975 | 3',4'-dihydroxy-7-methoxy-8-(3-methylbut-2-enyl)-2'''-(1-hydroxy-1-methylethyl)-furano-(4'',5'':6,5)favanone | 0.503610154 | B(iii) | 520.449 | 453.1904991 | POS | Flavonoids | 0.75 | 1.63 | 0.02 |
| 976 | mgmg 18:3 | 0.503598 | B(iii) | 716.765 | 559.3063245 | NEG | Glycerolipids | 0.01 | 0.01 | 0.01 |
| 977 | geranylgeranyl 1,4-hydroquinone | 0.503281 | B(iii) | 637.457 | 383.2931587 | POS | Meroterpenoids | 0.03 | 0.06 | 0.04 |
| 978 | pyridoxine + o-hex | 0.502533692 | B(iii) | 185.6935 | 332.1333859 | POS | Nicotinic acid alkaloids | 0.01 | 0.02 | 0.01 |
| 979 | piscidic acid | 0.501970077 | B(iii) | 343.414 | 255.0560072 | NEG |  | 0.37 | 0.22 | 0.12 |
| 980 | (2r,3s,4s,5r,6r)-5-[(2s,3r,4r)-3,4-dihydroxy-4-(hydroxymethyl)oxolan-2-yl]oxy-2-(hydroxymethyl)-6-(2-phenylethoxy)oxane-3,4-diol | 0.500492538 | B(iii) | 268.139 | 415.1594975 | NEG | Phenylethanoids | 0.00 | 0.00 | 0.00 |
| 981 | 3-(4-hydroxyphenyl)pyruvic acid | 0.500354538 | B(iii) | 244.9185 | 179.0347566 | NEG | Phenolic acids | 0.01 | 0.01 | 0.01 |
| 982 | magnolin | 0.5 | B(iii) | 379.1145 | 439.1749399 | POS | Lignans | 0.22 | 0.12 | 0.04 |
| 983 | methylnissolin-3-o-glucoside | 0.5 | B(iii) | 320.103 | 463.155413 | POS | Isoflavonoids | 0.20 | 0.04 | 0.01 |
| 984 | methylswertianin | 0.5 | B(iii) | 328.387 | 311.0489227 | POS | Xanthones | 0.17 | 0.11 | 0.29 |
| 985 | milleyanaflavone | 0.5 | B(iii) | 4.301585 | 297.0740953 | POS | Flavonoids | 0.02 | 0.03 | 0.03 |
| 986 | phillyrin | 0.5 | B(iii) | 257.923 | 557.1989642 | POS | Lignans | 0.00 | 0.00 | 0.00 |
| 987 | schizantherin a | 0.5 | B(iii) | 430.229 | 559.1967589 | POS | Lignans | 1.29 | 1.44 | 1.05 |
| 988 | prunetin | 0.5 | B(iii) | 405.108 | 285.0752063 | POS | Isoflavonoids | 4.23 | 7.09 | 2.84 |
| 989 | schisandrin c | 0.5 | B(iii) | 468.551 | 407.1489791 | POS | Lignans | 0.18 | 0.41 | 0.22 |
| 990 | benzothiazole | 0.5 | B(iii) | 378.525 | 136.0212404 | POS |  | 0.02 | 0.02 | 0.03 |
| 991 | 6-prenylnaringenin | 0.5 | B(iii) | 446.1875 | 363.1218703 | POS | Flavonoids | 0.20 | 0.07 | 0.06 |
| 992 | 4',7-di-o-methylnaringenin | 0.5 | B(iii) | 382.307 | 323.0889404 | POS | Flavonoids | 0.13 | 0.17 | 0.09 |
| 993 | 4-methylpyrimidine | 0.5 | B(iii) | 46.3545 | 95.06007861 | POS | Nicotinic acid alkaloids | 0.01 | 0.00 | 0.00 |
| 994 | 3-(3,4-dimethoxyphenyl)-7-hydroxy-4h-chromen-4-one | 0.5 | B(iii) | 383.8755 | 299.0903613 | POS | Isoflavonoids | 8.51 | 9.36 | 4.04 |
| 995 | allothreonine | 0.5 | B(iii) | 41.68835 | 120.0651651 | POS | Small peptides | 0.02 | 0.03 | 0.03 |
| 996 | 7-o-methylchrysin | 0.5 | B(iii) | 435.238 | 269.0798761 | POS | Flavonoids | 0.64 | 0.78 | 0.55 |
| 997 | cimifugin | 0.5 | B(iii) | 327.115 | 329.1015988 | POS | Chromanes | 0.04 | 0.12 | 0.20 |
| 998 | isoeugenol acetate | 0.5 | B(iii) | 341.554 | 207.1008298 | POS | Phenylpropanoids | 0.05 | 0.12 | 0.09 |
| 999 | cis-3-hydroxy-dl-proline | 0.5 | B(iii) | 43.4372 | 132.0653483 | POS | Small peptides | 0.06 | 0.08 | 0.06 |
| 1000 | 1-hydroxy-2-naphthoic acid | 0.5 | B(iii) | 45.8438 | 187.037501 | NEG | Naphthalenes | 0.03 | 0.02 | 0.02 |
| 1001 | trans-cinnamic acid | 0.5 | B(iii) | 360.444 | 147.0446649 | NEG | Phenylpropanoids | 0.03 | 0.11 | 0.01 |
| 1002 | maleamic acid | 0.5 | B(iii) | 6.62594 | 114.019291 | NEG | Fatty Acids and Conjugates | 0.03 | 0.03 | 0.03 |
| 1003 | fumaric acid | 0.5 | B(iii) | 74.78915 | 115.0034606 | NEG | Fatty Acids and Conjugates | 0.00 | 0.00 | 0.00 |
| 1004 | dihydrocoumarin | 0.5 | B(iii) | 2.630315 | 147.0447321 | NEG | Flavonoids | 0.01 | 0.01 | 0.01 |
| 1005 | 4'-hydroxy-2',4,6'-trimethoxychalcone | 0.499997308 | B(iii) | 356.434 | 315.121577 | POS | Flavonoids | 2.87 | 3.85 | 1.03 |
| 1006 | guanosine | 0.499899077 | B(iii) | 184.89 | 282.0835992 | NEG | Nucleosides | 0.00 | 0.00 | 0.00 |
| 1007 | tocotrienol | 0.499560769 | B(iii) | 660.653 | 397.3093632 | POS |  | 0.37 | 0.54 | 0.18 |
| 1008 | liquiritigenin | 0.499427385 | B(iii) | 44.7384 | 255.0716075 | NEG | Flavonoids | 0.01 | 0.01 | 0.01 |
| 1009 | nicotinic acid | 0.49937 | B(iii) | 239.827 | 122.0245424 | NEG | Nicotinic acid alkaloids | 0.02 | 0.04 | 0.01 |
| 1010 | 2-methylcitrate | 0.498841538 | B(iii) | 43.5009 | 205.0348668 | NEG | Fatty Acids and Conjugates | 0.03 | 0.02 | 0.02 |
| 1011 | caffeic acid; lc-tdda; ce10 | 0.498630538 | B(iii) | 263.654 | 179.0347509 | NEG | Phenylpropanoids | 0.01 | 0.01 | 0.01 |
| 1012 | (1s,4as,7s,7as)-7-hydroxy-7-methyl-1-[(2s,3r,4s,5s,6r)-3,4,5-trihydroxy-6-(hydroxymethyl)oxan-2-yl]oxy-4a,5,6,7a-tetrahydro-1h-cyclopenta[c]pyran-4-carboxylic acid | 0.498565231 | B(iii) | 398.612 | 377.1379485 | POS | Monoterpenoids | 1.42 | 0.35 | 1.20 |
| 1013 | fraxetin | 0.498364769 | B(iii) | 222.531 | 207.0295356 | NEG | Coumarins | 0.00 | 0.00 | 0.00 |
| 1014 | khelloside | 0.498303269 | B(iii) | 130.133 | 409.1099105 | POS | Chromanes | 0.00 | 0.00 | 0.01 |
| 1015 | xanthosine | 0.497569231 | B(iii) | 48.4854 | 285.0847199 | POS | Nucleosides | 0.01 | 0.02 | 0.01 |
| 1016 | [(3s,4r,5s)-5-[(2s,3r,4s,5s,6r)-4,5-dihydroxy-6-(hydroxymethyl)-2-(4-hydroxyphenoxy)oxan-3-yl]oxy-3,4-dihydroxyoxolan-3-yl]methyl 4-hydroxybenzoate | 0.497143192 | B(iii) | 80.2753 | 523.1436181 | NEG |  | 0.00 | 0.00 | 0.01 |
| 1017 | ceroptene | 0.497093769 | B(iii) | 469.202 | 299.1273595 | POS | Others | 0.19 | 0.21 | 0.39 |
| 1018 | kinetin | 0.497064154 | B(iii) | 376.2815 | 233.1142201 | POS | Pseudoalkaloids | 0.49 | 0.42 | 0.17 |
| 1019 | traumatic acid | 0.496786769 | B(iii) | 266.179 | 229.1429061 | POS | Fatty Acids and Conjugates | 0.06 | 0.03 | 0.01 |
| 1020 | 3-aminoisobutyric acid | 0.496402077 | B(iii) | 465.199 | 104.0702692 | POS | Small peptides | 0.20 | 0.16 | 0.34 |
| 1021 | na-methylhistamine | 0.496147308 | B(iii) | 742.75 | 126.1022043 | POS | Histidine alkaloids | 0.20 | 0.34 | 0.24 |
| 1022 | 4-methoxycinnamic acid | 0.495520731 | B(iii) | 344.8095 | 177.0552753 | NEG | Phenylpropanoids | 0.30 | 0.16 | 0.16 |
| 1023 | epigallocatechin 3-o-p-coumarate | 0.494617308 | B(iii) | 200.199 | 453.1266009 | POS | Flavonoids | 0.00 | 0.00 | 0.11 |
| 1024 | hesperidin | 0.494476885 | B(iii) | 427.7285 | 609.2093375 | NEG | Flavonoids | 0.02 | 0.01 | 0.03 |
| 1025 | polyphyllin vi | 0.493808577 | B(iii) | 344.782 | 739.423652 | POS | Steroids | 0.07 | 0.08 | 0.24 |
| 1026 | 4',5,7-trihydroxy-3,6-dimethoxyflavone | 0.493534231 | B(iii) | 197.677 | 331.0784082 | POS | Flavonoids | 0.32 | 0.26 | 0.39 |
| 1027 | (2s)-4-hydroxy-2-(2-hydroxypropan-2-yl)-7-methyl-2,3-dihydrofuro[3,2-g]chromen-5-one | 0.493217154 | B(iii) | 296.529 | 315.0625881 | POS | Chromanes | 0.20 | 0.60 | 0.13 |
| 1028 | propylparaben | 0.492992154 | B(iii) | 520.9875 | 181.0855816 | POS | Phenolic acids | 1.81 | 4.39 | 0.09 |
| 1029 | 8-acetylharpagide | 0.492597615 | B(iii) | 383.45 | 407.148958 | POS | Monoterpenoids | 0.38 | 0.92 | 0.81 |
| 1030 | 5,6,7,8,3',4',5'-heptamethoxyflavanone | 0.491809538 | B(iii) | 334.354 | 435.1635455 | POS | Flavonoids | 1.23 | 1.13 | 1.98 |
| 1031 | isobrucein a | 0.491179154 | B(iii) | 499.4505 | 523.2097848 | POS | Triterpenoids | 0.61 | 1.79 | 0.47 |
| 1032 | leucinic acid | 0.491026692 | B(iii) | 213.762 | 131.0709358 | NEG | Fatty Acids and Conjugates | 0.01 | 0.01 | 0.00 |
| 1033 | paeoniflorin | 0.490508923 | B(iii) | 383.6105 | 481.1635269 | POS | Monoterpenoids | 0.30 | 0.19 | 0.08 |
| 1034 | angophorol | 0.490177615 | B(iii) | 397.245 | 315.1216399 | POS | Flavonoids | 0.09 | 0.10 | 0.66 |
| 1035 | feruloyl hexoside (isomer of 849) | 0.488220769 | B(iii) | 232.686 | 355.1025795 | NEG | Phenylpropanoids | 0.00 | 0.00 | 0.00 |
| 1036 | methyl 2-[4-ethenyl-2,6-dihydroxy-3-(3-hydroxyprop-1-en-2-yl)-4-methylcyclohexyl]prop-2-enoate | 0.487499 | B(iii) | 290.301 | 319.1507343 | POS | Sesquiterpenoids | 0.08 | 0.07 | 0.07 |
| 1037 | trans-piceid | 0.487455577 | B(iii) | 200.734 | 389.1215798 | NEG | Stilbenoids | 0.01 | 0.01 | 0.14 |
| 1038 | cyanidin-3-o-glucoside | 0.487057962 | B(iii) | 197.2455 | 449.1054435 | POS | Others | 0.01 | 0.00 | 0.00 |
| 1039 | palmitic acid | 0.486775692 | B(iii) | 577.248 | 279.2313654 | POS | Fatty Acids and Conjugates | 0.35 | 0.36 | 0.23 |
| 1040 | ellipticine | 0.486636038 | B(iii) | 376.3155 | 269.1071503 | POS | Tryptophan alkaloids | 0.33 | 0.71 | 0.49 |
| 1041 | nicotinamide; ce10; dfpaksucgfbddf-uhfffaoysa-n | 0.486362692 | B(iii) | 180.947 | 123.0550369 | POS | Nicotinic acid alkaloids | 0.04 | 0.05 | 0.04 |
| 1042 | benzoylgomisin o | 0.486211692 | B(iii) | 488.2155 | 543.1994581 | POS | Others | 0.56 | 1.35 | 0.05 |
| 1043 | verproside | 0.485990077 | B(iii) | 245.465 | 497.1429541 | NEG | Monoterpenoids | 0.00 | 0.00 | 0.00 |
| 1044 | 7,8,3',4',5'-pentamethoxy-6'',6''-dimethylpyrano[2'',3'':5,6]flavone | 0.485711846 | B(iii) | 320.1785 | 455.1686777 | POS | Flavonoids | 2.97 | 0.68 | 0.03 |
| 1045 | (2e)-3-(4-{[(2s,3r,4s,5s,6r)-3-{[(2s,3r,4r)-3,4-dihydroxy-4-(hydroxymethyl)oxolan-2-yl]oxy}-4,5-dihydroxy-6-(hydroxymethyl)oxan-2-yl]oxy}phenyl)-1-(2,4-dihydroxyphenyl)prop-2-en-1-one | 0.485387 | B(iii) | 628.573 | 551.1707415 | POS | Flavonoids | 0.60 | 0.60 | 0.79 |
| 1046 | (1r,4r,6r,10s)-4,12,12-trimethyl-9-methylene-5-oxatricyclo[8.2.0.0~4,6~]dodecane | 0.485016615 | B(iii) | 465.195 | 221.1894502 | POS | Sesquiterpenoids | 0.04 | 0.00 | 0.01 |
| 1047 | cucumerin b | 0.483955308 | B(iii) | 402.5555 | 553.1845066 | POS | Flavonoids | 6.37 | 3.58 | 2.24 |
| 1048 | dopamine [m+h-nh2]+; aif; ce0; ms2dec | 0.483673654 | B(iii) | 519.7325 | 137.0593287 | POS | Tyrosine alkaloids | 12.64 | 17.92 | 0.37 |
| 1049 | cucurbitacin q | 0.482496923 | B(iii) | 605.5065 | 561.3416574 | POS | Triterpenoids | 0.05 | 0.03 | 0.01 |
| 1050 | (4~{a}~{s},6~{a}~{s},6~{b}~{r},10~{s},12~{a}~{r})-10-hydroxy-2,2,6~{a},6~{b},9,9,12~{a}-heptamethyl-1,3,4,5,6,6~{a},7,8,8~{a},10,11,12,13,14~{b}-tetradecahydropicene-4~{a}-carboxylic acid | 0.482217769 | B(iii) | 752.36 | 439.3571394 | POS |  | 0.08 | 0.08 | 0.05 |
| 1051 | triptophenolide | 0.482027 | B(iii) | 487.294 | 311.1677051 | NEG | Diterpenoids | 0.07 | 0.06 | 0.06 |
| 1052 | 1,5,8-trihydroxy-3-methyl-2-prenylxanthone | 0.480967 | B(iii) | 423.409 | 343.116556 | POS | Xanthones | 0.03 | 0.14 | 0.04 |
| 1053 | nomilin | 0.480598538 | B(iii) | 270.907 | 513.2100194 | NEG | Triterpenoids | 0.01 | 0.00 | 0.00 |
| 1054 | narirutin | 0.480181538 | B(iii) | 279.233 | 581.1860983 | POS | Flavonoids | 0.01 | 0.01 | 0.00 |
| 1055 | 2-methylpyridine | 0.479285538 | B(iii) | 728.577 | 94.06486076 | POS | Nicotinic acid alkaloids | 0.03 | 0.03 | 0.03 |
| 1056 | peimine | 0.478593308 | B(iii) | 751.9185 | 432.3543334 | POS | Pseudoalkaloids | 0.09 | 0.12 | 0.08 |
| 1057 | annohexocin | 0.478254385 | B(iii) | 695.689 | 629.4779226 | POS | Linear polyketides | 0.09 | 0.12 | 0.09 |
| 1058 | ovatine | 0.476493308 | B(iii) | 615.427 | 386.2675281 | POS | Diterpenoids | 0.00 | 0.00 | 0.25 |
| 1059 | altenusin | 0.474622346 | B(iii) | 225.343 | 289.0707906 | NEG | Phenolic acids | 0.00 | 0.00 | 0.00 |
| 1060 | pinocembrin 7-rhamnosylglucoside | 0.474532615 | B(iii) | 372.603 | 565.1834046 | POS | Flavonoids | 0.46 | 0.48 | 1.28 |
| 1061 | lindleyin | 0.474429269 | B(iii) | 281.233 | 479.1529355 | POS | Phenolic acids | 0.01 | 0.00 | 0.00 |
| 1062 | jaeschkeanadiol | 0.474307846 | B(iii) | 492.841 | 256.2268651 | POS | Sesquiterpenoids | 0.05 | 0.04 | 0.05 |
| 1063 | kushenol c | 0.474098231 | B(iii) | 335.197 | 439.1745648 | POS | Flavonoids | 0.12 | 0.02 | 0.04 |
| 1064 | 3,7,11,15-tetramethyl-2e,6e,10e,14-hexadecatetraenal | 0.473058077 | B(iii) | 589.463 | 289.2524332 | POS | Fatty acyls | 0.03 | 0.04 | 0.01 |
| 1065 | oxypinnatanine | 0.471185385 | B(iii) | 307.72 | 261.1115961 | POS | Small peptides | 0.02 | 0.05 | 0.02 |
| 1066 | (2r,3r)-3,5-dihydroxy-2-(4-hydroxyphenyl)-8-(3-methylbut-2-en-1-yl)-7-(((2s,3r,4s,5s,6r)-3,4,5-trihydroxy-6-(hydroxymethyl)tetrahydro-2h-pyran-2-yl)oxy)chroman-4-one | 0.470653385 | B(iii) | 313.013 | 541.1671008 | POS | Flavonoids | 0.03 | 0.03 | 0.01 |
| 1067 | c26:1n-9 | 0.469220385 | B(iii) | 702.103 | 395.3880695 | POS | Fatty Acids and Conjugates | 0.05 | 0.22 | 0.07 |
| 1068 | biochanin a | 0.468851385 | B(iii) | 779.3695 | 283.0602649 | NEG | Isoflavonoids | 0.02 | 0.01 | 0.01 |
| 1069 | sinensetin | 0.468678 | B(iii) | 359.59 | 371.1122193 | NEG | Flavonoids | 0.01 | 0.01 | 0.03 |
| 1070 | multifidol 2-[apiosyl-(1->6)-glucoside] | 0.468082769 | B(iii) | 346.755 | 505.1852177 | POS |  | 0.09 | 0.31 | 0.01 |
| 1071 | platyphyllenone | 0.467147538 | B(iii) | 328.507 | 319.130234 | POS | Diarylheptanoids | 0.08 | 0.06 | 0.03 |
| 1072 | dihydrocoumaroyl hexoside | 0.467137692 | B(iii) | 207.631 | 327.1142027 | NEG | Phenolic acids | 0.00 | 0.00 | 0.00 |
| 1073 | s-adenosyl-homocysteine | 0.465758692 | B(iii) | 49.365 | 383.1187776 | NEG | Nucleosides | 0.15 | 0.13 | 0.08 |
| 1074 | 1-[4-hydroxy-3-(3-methylbut-2-enyl)phenyl]ethanone | 0.464939654 | B(iii) | 12.86205 | 205.0855802 | POS | Others | 0.02 | 0.02 | 0.02 |
| 1075 | daphylloside | 0.464075692 | B(iii) | 500.7655 | 447.1587257 | POS | Monoterpenoids | 0.20 | 0.23 | 0.02 |
| 1076 | avenanthramide 1f | 0.462802692 | B(iii) | 344.334 | 314.0998253 | POS | Phenylpropanoids | 1.42 | 1.40 | 1.69 |
| 1077 | gambiriin b3 | 0.462739846 | B(iii) | 394.5915 | 563.1458783 | POS | Flavonoids | 2.69 | 1.65 | 2.23 |
| 1078 | 2-aminobutyric acid; lc-tdda; ce20 | 0.462288077 | B(iii) | 480.2885 | 104.0702392 | POS | Small peptides | 0.33 | 0.24 | 0.16 |
| 1079 | 5-o-methylembelin | 0.462246154 | B(iii) | 418.7155 | 309.2054054 | POS | Aromatic polyketides | 0.05 | 0.03 | 0.21 |
| 1080 | (2r,3r,4s,5s,6r)-2-[(3r)-1,7-bis(3,4-dihydroxyphenyl)heptan-3-yl]oxy-6-(hydroxymethyl)oxane-3,4,5-triol | 0.461926154 | B(iii) | 286.595 | 517.2048981 | POS | Diarylheptanoids | 0.21 | 0.03 | 0.00 |
| 1081 | flindersiachromone | 0.459794615 | B(iii) | 496.279 | 251.1062757 | POS | Chromanes | 0.06 | 0.02 | 0.00 |
| 1082 | chelerythrine | 0.458937231 | B(iii) | 305.515 | 371.1101293 | POS | Others | 0.10 | 0.10 | 0.08 |
| 1083 | duartin | 0.458738615 | B(iii) | 372.643 | 333.1327105 | POS | Isoflavonoids | 0.15 | 0.14 | 0.27 |
| 1084 | 4-methoxybenzyl acetate | 0.458400692 | B(iii) | 784.733 | 181.0853947 | POS | Others | 0.50 | 0.91 | 0.22 |
| 1085 | (2~{r},3~{r})-2-(3,4-dihydroxyphenyl)-5,7-dihydroxy-3-[(2~{s},3~{r},4~{s},5~{s},6~{r})-3,4,5-trihydroxy-6-(hydroxymethyl)oxan-2-yl]oxy-2,3-dihydrochromen-4-one | 0.458386154 | B(iii) | 328.625 | 489.0940662 | POS |  | 0.01 | 0.01 | 0.12 |
| 1086 | fistuloside b | 0.457533846 | B(iii) | 335.726 | 901.4586689 | POS | Steroids | 0.02 | 0.02 | 0.01 |
| 1087 | lethedoside b | 0.456541077 | B(iii) | 518.438 | 521.1573365 | POS | Flavonoids | 0.50 | 1.45 | 0.23 |
| 1088 | theobromine | 0.456132077 | B(iii) | 43.6015 | 219.0172603 | POS | Pseudoalkaloids | 1.00 | 0.64 | 0.57 |
| 1089 | schidigerasaponin c2 | 0.454321154 | B(iii) | 308.168 | 755.4178727 | POS | Steroids | 0.30 | 1.45 | 0.03 |
| 1090 | italidipyrone | 0.454293385 | B(iii) | 470.483 | 543.23727 | POS | Phloroglucinols | 9.64 | 4.32 | 6.41 |
| 1091 | methylophiopogonone a | 0.454261269 | B(iii) | 376.154 | 341.1010786 | POS | Chromanes | 0.12 | 0.20 | 0.19 |
| 1092 | biochanin-7-o-glucoside | 0.453854615 | B(iii) | 336.337 | 445.1128532 | NEG | Isoflavonoids | 0.00 | 0.00 | 0.00 |
| 1093 | karanjin | 0.453710385 | B(iii) | 409.411 | 315.0627477 | POS | Flavonoids | 0.31 | 0.40 | 0.17 |
| 1094 | 3-hydroxy-4-methoxycinnamic acid | 0.453516769 | B(iii) | 283.894 | 193.050043 | NEG | Phenylpropanoids | 0.04 | 0.10 | 0.03 |
| 1095 | dibutylhydroxytoluene | 0.453152846 | B(iii) | 617.406 | 221.189474 | POS | Others | 0.02 | 0.01 | 0.01 |
| 1096 | sinapic acid; plasma id-325 | 0.452512115 | B(iii) | 221.891 | 223.0603981 | NEG | Phenylpropanoids | 0.00 | 0.01 | 0.00 |
| 1097 | dormatinol | 0.452143769 | B(iii) | 628.067 | 419.3519005 | POS | Steroids | 0.60 | 0.53 | 0.93 |
| 1098 | embelin | 0.451063615 | B(iii) | 441.559 | 293.1751477 | NEG | Aromatic polyketides | 0.33 | 0.36 | 0.31 |
| 1099 | ricinine | 0.450092077 | B(iii) | 460.705 | 182.0888943 | POS | Nicotinic acid alkaloids | 19.07 | 35.17 | 0.63 |
| 1100 | 8-desoxygartanin | 0.449454692 | B(iii) | 515.2055 | 403.1542213 | POS | Xanthones | 0.10 | 0.03 | 0.23 |
| 1101 | 2-[2-[1-carboxy-2-(3,4-dihydroxyphenyl)ethoxy]carbonyl-3,4-bis(3,4-dihydroxyphenyl)cyclobutanecarbonyl]oxy-3-(3,4-dihydroxyphenyl)propanoic acid | 0.447538231 | B(iii) | 754.405 | 719.1468969 | NEG |  | 0.00 | 0.00 | 0.00 |
| 1102 | seconeokadsuranic acid a | 0.447522462 | B(iii) | 551.3995 | 469.3292325 | POS | Triterpenoids | 0.02 | 0.01 | 0.01 |
| 1103 | laurinterol | 0.447427846 | B(iii) | 465.246 | 295.0728664 | POS | Sesquiterpenoids | 0.44 | 0.50 | 0.01 |
| 1104 | methyl (1s,4ar,7ar)-4a-hydroxy-7-(hydroxymethyl)-1-[(2s,3r,4s,5s,6r)-3,4,5-trihydroxy-6-(hydroxymethyl)oxan-2-yl]oxy-5,7a-dihydro-1h-cyclopenta[c]pyran-4-carboxylate | 0.447007231 | B(iii) | 384.796 | 405.1320183 | POS | Monoterpenoids | 0.54 | 0.96 | 0.78 |
| 1105 | aspalathin | 0.44607 | B(iii) | 295.194 | 451.1241538 | NEG | Flavonoids | 0.03 | 0.00 | 0.00 |
| 1106 | 8-dimethylallyllisetin | 0.445364077 | B(iii) | 519.25 | 451.1735753 | POS | Isoflavonoids | 0.17 | 0.16 | 0.25 |
| 1107 | (13r,14r)-8-labdene-13,14,15-triol | 0.445232 | B(iii) | 616.109 | 325.2736574 | POS | Diterpenoids | 0.04 | 0.04 | 0.05 |
| 1108 | gomisin a | 0.444235846 | B(iii) | 433.552 | 439.174894 | POS | Lignans | 2.16 | 1.19 | 1.39 |
| 1109 | stevioside | 0.443523846 | B(iii) | 288.323 | 827.3818011 | POS | Diterpenoids | 0.01 | 0.01 | 0.00 |
| 1110 | antiarone c | 0.443444077 | B(iii) | 406.384 | 439.2081161 | POS | Flavonoids | 0.13 | 0.13 | 0.22 |
| 1111 | kuwanone g | 0.440567308 | B(iii) | 397.067 | 693.2303048 | POS | Flavonoids | 0.02 | 0.09 | 0.03 |
| 1112 | 15-keto-prostaglandin e2 | 0.440029692 | B(iii) | 634.435 | 349.2040876 | NEG | Eicosanoids | 0.03 | 0.03 | 0.04 |
| 1113 | onopordopicrin | 0.440026846 | B(iii) | 479.6745 | 387.1216229 | POS | Sesquiterpenoids | 0.19 | 0.24 | 0.00 |
| 1114 | flavone + 2o + 2meo | 0.438443269 | B(iii) | 365.236 | 313.0705871 | NEG | Flavonoids | 0.08 | 0.08 | 0.09 |
| 1115 | luteolinidin | 0.437362846 | B(iii) | 388.697 | 272.0628435 | POS | Others | 1.72 | 3.54 | 0.41 |
| 1116 | 11,12,13-trinor-7-calamenone | 0.437353462 | B(iii) | 375.635 | 175.1114462 | POS |  | 0.50 | 0.42 | 0.15 |
| 1117 | [(3ar,4s,6e,10z,11ar)-10-(hydroxymethyl)-6-methyl-3-methylidene-2-oxo-3a,4,5,8,9,11a-hexahydrocyclodeca[b]furan-4-yl] (z)-4-acetyloxy-2-(hydroxymethyl)but-2-enoate | 0.437350923 | B(iii) | 361.8915 | 443.1663814 | POS | Sesquiterpenoids | 0.15 | 0.06 | 0.07 |
| 1118 | khivorin | 0.435936462 | B(iii) | 610.151 | 587.2981287 | POS | Triterpenoids | 0.57 | 0.72 | 0.58 |
| 1119 | 4-methoxy-9-(3-methylbut-2-enyl)furo[3,2-g]chromen-7-one | 0.435050346 | B(iii) | 358.998 | 285.1111278 | POS | Coumarins | 10.44 | 14.20 | 10.52 |
| 1120 | brosimacutin i | 0.434726154 | B(iii) | 388.178 | 361.164158 | POS | Flavonoids | 0.05 | 0.09 | 0.01 |
| 1121 | pinoresinol 4-o-glucoside | 0.434306231 | B(iii) | 554.527 | 521.196116 | POS | Lignans | 1.57 | 0.57 | 0.29 |
| 1122 | schizantherin b | 0.434201423 | B(iii) | 474.3745 | 515.2339023 | POS | Lignans | 0.17 | 0.04 | 0.01 |
| 1123 | 9z,12z-octadecadienal | 0.433766538 | B(iii) | 649.497 | 265.2517618 | POS | Fatty acyls | 1.65 | 1.43 | 2.13 |
| 1124 | glabranin | 0.43367 | B(iii) | 397.2475 | 347.1269112 | POS | Coumarins | 0.51 | 0.10 | 0.05 |
| 1125 | xanthohumol | 0.432628 | B(iii) | 200.771 | 353.1442425 | NEG | Flavonoids | 0.00 | 0.00 | 0.02 |
| 1126 | artonol c | 0.432337077 | B(iii) | 370.311 | 501.1905723 | POS | Flavonoids | 0.39 | 1.07 | 0.04 |
| 1127 | 1,5-anhydro-glucitol | 0.430289846 | B(iii) | 44.4089 | 165.0754865 | POS | Saccharides | 0.43 | 0.80 | 0.75 |
| 1128 | angelol b | 0.428634769 | B(iii) | 407.217 | 399.1434964 | POS | Coumarins | 0.09 | 0.31 | 0.02 |
| 1129 | bisdemethoxycurcumin | 0.428340385 | B(iii) | 403.1085 | 309.1090074 | POS | Diarylheptanoids | 0.18 | 0.04 | 0.49 |
| 1130 | episyringaresinol 4'-o-beta-d-glncopyranoside | 0.425444192 | B(iii) | 309.767 | 603.2041032 | POS | Lignans | 0.16 | 0.04 | 0.06 |
| 1131 | veratramine | 0.425163 | B(iii) | 758.624 | 410.3134978 | POS | Lysine alkaloids | 0.10 | 0.12 | 0.07 |
| 1132 | 2,3,4,2',4',6'-hexamethoxychalcone | 0.424824154 | B(iii) | 324.064 | 389.1592978 | POS | Flavonoids | 0.11 | 0.05 | 0.03 |
| 1133 | 5'-prenylhomoeriodictyol | 0.424725692 | B(iii) | 384.305 | 371.1487704 | POS | Isoflavonoids | 0.04 | 0.04 | 0.03 |
| 1134 | 3-[5,7-dihydroxy-2-(4-methoxyphenyl)-4-oxo-2,3-dihydrochromen-3-yl]-5,7-dihydroxy-2-(4-methoxyphenyl)-2,3-dihydrochromen-4-one | 0.423020923 | B(iii) | 351.2205 | 569.1445222 | NEG | Flavonoids | 0.09 | 0.04 | 0.02 |
| 1135 | methyl (1~{s},3~{r},4~{r},5~{r})-3-[(~{e})-3-(3,4-dihydroxyphenyl)prop-2-enoyl]oxy-1,4,5-trihydroxycyclohexane-1-carboxylate | 0.422064692 | B(iii) | 482.9305 | 369.1097243 | POS |  | 1.25 | 0.87 | 0.44 |
| 1136 | crotonic acid | 0.422015 | B(iii) | 782.386 | 85.0293179 | NEG | Fatty Acids and Conjugates | 0.02 | 0.02 | 0.02 |
| 1137 | arabinofuranosyluracil | 0.420860923 | B(iii) | 107.3375 | 243.0616767 | NEG | Nucleosides | 0.01 | 0.01 | 0.01 |
| 1138 | malvidin 3-(6-acetylglucoside) | 0.420849769 | B(iii) | 654.086 | 536.1649248 | POS | Others | 0.12 | 0.11 | 0.13 |
| 1139 | yangambin | 0.420306269 | B(iii) | 373.692 | 469.1847581 | POS | Lignans | 1.33 | 0.22 | 0.15 |
| 1140 | 5-hydroxy-8,8-dimethyl-6-(2-methylbutanoyl)-4-phenylpyrano[2,3-h]chromen-2-one | 0.419897846 | B(iii) | 465.863 | 405.16879 | POS |  | 0.08 | 0.13 | 0.43 |
| 1141 | [(1as,1bs,2s,5ar,6s,6as)-1a-(hydroxymethyl)-2-[(2s,3r,4s,5s,6r)-3,4,5-trihydroxy-6-(hydroxymethyl)oxan-2-yl]oxy-2,5a,6,6a-tetrahydro-1bh-oxireno[5,6]cyclopenta[1,3-c]pyran-6-yl] benzoate | 0.419695538 | B(iii) | 464.615 | 489.1299902 | POS | Monoterpenoids | 0.12 | 0.51 | 0.05 |
| 1142 | 8-(2,3-dihydroxy-3-methylbutyl)-7-methoxychromen-2-one | 0.419519385 | B(iii) | 384.957 | 317.0783604 | POS | Coumarins | 0.04 | 0.04 | 0.15 |
| 1143 | garcinone d | 0.418404615 | B(iii) | 442.757 | 451.1734055 | POS | Xanthones | 0.60 | 0.77 | 1.95 |
| 1144 | daphnetin-8-glucoside | 0.417746423 | B(iii) | 193.864 | 339.0657925 | NEG | Coumarins | 0.00 | 0.00 | 0.00 |
| 1145 | 2,4-di-tert-butylphenol | 0.417707385 | B(iii) | 583.109 | 205.1592704 | NEG | Flavonoids | 0.33 | 0.32 | 0.32 |
| 1146 | methyl 1,4~{a}-dimethyl-6-methylidene-5-[2-(5-oxo-2~{h}-furan-4-yl)ethyl]-3,4,5,7,8,8~{a}-hexahydro-2~{h}-naphthalene-1-carboxylate | 0.416821692 | B(iii) | 350.826 | 347.2210836 | POS |  | 0.03 | 0.07 | 0.00 |
| 1147 | cis-11,14-eicosadienoic acid | 0.416732923 | B(iii) | 800.727 | 292.9849621 | POS | Fatty Acids and Conjugates | 0.34 | 0.37 | 0.35 |
| 1148 | epimedoside c | 0.416093462 | B(iii) | 498.3345 | 517.1628835 | POS | Flavonoids | 0.17 | 0.54 | 0.22 |
| 1149 | prostaglandin e1 | 0.415124385 | B(iii) | 705.867 | 690.4784792 | NEG | Eicosanoids | 0.00 | 0.00 | 0.00 |
| 1150 | anofinic acid | 0.413428692 | B(iii) | 273.044 | 205.0856688 | POS | Meroterpenoids | 0.03 | 0.02 | 0.03 |
| 1151 | schleicherastatin 6 | 0.412886615 | B(iii) | 673.715 | 431.3520651 | POS | Steroids | 0.65 | 0.61 | 0.16 |
| 1152 | mahanimbine | 0.412555154 | B(iii) | 516.6075 | 332.1983935 | POS | Tryptophan alkaloids | 0.29 | 0.30 | 0.37 |
| 1153 | gossyvertin | 0.412016 | B(iii) | 354.6055 | 275.1271449 | POS | Sesquiterpenoids | 1.99 | 1.16 | 2.30 |
| 1154 | spinoflavanone a | 0.411756 | B(iii) | 428.66 | 389.1735101 | POS | Flavonoids | 1.61 | 0.12 | 0.02 |
| 1155 | 2-acetyl-6-[(3-butanoyl-2,4,6-trihydroxy-5-methylphenyl)methyl]-3,5-dihydroxy-4,4-dimethylcyclohexa-2,5-dien-1-one | 0.411342692 | B(iii) | 365.315 | 417.1549109 | NEG | Phloroglucinols | 0.58 | 0.52 | 0.47 |
| 1156 | isoscopoletin | 0.411145462 | B(iii) | 273.116 | 191.0346254 | NEG | Coumarins | 0.01 | 0.01 | 0.01 |
| 1157 | agavoside b | 0.410293923 | B(iii) | 349.3675 | 755.4175298 | POS | Steroids | 0.10 | 0.39 | 0.03 |
| 1158 | (1e,4z,6e)-5-hydroxy-1,7-bis(4-hydroxy-3-methoxyphenyl)hepta-1,4,6-trien-3-one | 0.410086385 | B(iii) | 369.599 | 367.1172273 | NEG | Diarylheptanoids | 0.04 | 0.02 | 0.07 |
| 1159 | hovenidulcioside a1 | 0.409039692 | B(iii) | 339.606 | 853.4563538 | POS | Triterpenoids | 0.01 | 0.02 | 0.00 |
| 1160 | 6,7-dimethoxy-2,2-dimethyl-2h-1-benzopyran | 0.408422192 | B(iii) | 310.76 | 243.1008081 | POS | Meroterpenoids | 0.08 | 0.04 | 0.08 |
| 1161 | fa 18:1+1o | 0.408085615 | B(iii) | 600.3335 | 297.243025 | NEG | Octadecanoids | 0.10 | 0.10 | 0.07 |
| 1162 | artobiloxanthone | 0.407510615 | B(iii) | 405.224 | 435.1424918 | POS | Flavonoids | 0.18 | 0.35 | 0.42 |
| 1163 | adunctin a | 0.406692923 | B(iii) | 498.818 | 409.2337283 | POS | Flavonoids | 0.31 | 1.34 | 0.08 |
| 1164 | 2-(3,4-dimethoxyphenyl)-5,6,7,8-tetramethoxy-4h-chromen-4-one | 0.406303269 | B(iii) | 359.263 | 425.1223353 | POS | Flavonoids | 0.01 | 0.02 | 0.04 |
| 1165 | isocitric acid | 0.404202 | B(iii) | 42.2566 | 191.0194369 | NEG | Fatty Acids and Conjugates | 0.02 | 0.03 | 0.03 |
| 1166 | dihydroshikonofuran | 0.403687846 | B(iii) | 323.79 | 261.1481159 | POS | Meroterpenoids | 0.04 | 0.08 | 1.00 |
| 1167 | 11-epi azadirachtin d | 0.402081385 | B(iii) | 434.0965 | 659.2608991 | POS | Triterpenoids | 0.10 | 0.10 | 1.28 |
| 1168 | androstenedione | 0.401367308 | B(iii) | 423.4255 | 287.1996655 | POS | Steroids | 0.01 | 0.26 | 0.97 |
| 1169 | lucidine b | 0.401181462 | B(iii) | 619.274 | 468.388987 | POS | Lysine alkaloids | 0.16 | 0.13 | 0.13 |
| 1170 | artemisinin | 0.399314154 | B(iii) | 393.941 | 305.1280863 | POS | Sesquiterpenoids | 1.00 | 1.36 | 0.27 |
| 1171 | (2z)-3-(4-methoxy-2-{[(2s,3r,4s,5s,6r)-3,4,5-trihydroxy-6-(hydroxymethyl)oxan-2-yl]oxy}phenyl)prop-2-enoic acid | 0.397823154 | B(iii) | 186.797 | 379.0997765 | POS | Phenylpropanoids | 0.07 | 0.18 | 0.00 |
| 1172 | 5-methylcytosine hydrocloride | 0.396655462 | B(iii) | 649.55 | 124.0513136 | NEG | Nicotinic acid alkaloids | 0.03 | 0.03 | 0.04 |
| 1173 | brazilein | 0.394641692 | B(iii) | 359.046 | 302.1094034 | POS | Others | 0.76 | 2.58 | 1.07 |
| 1174 | mesaconitine | 0.393579231 | B(iii) | 579.117 | 632.3224157 | POS | Pseudoalkaloids | 0.03 | 0.04 | 0.00 |
| 1175 | 9-methoxy-7-[4-[3,4,5-trihydroxy-6-[[3,4,5-trihydroxy-6-(hydroxymethyl)oxan-2-yl]oxymethyl]oxan-2-yl]oxyphenyl]-[1,3]dioxolo[4,5-g]chromen-8-one | 0.393555923 | B(iii) | 273.8755 | 659.1939008 | POS | Isoflavonoids | 0.03 | 0.03 | 0.02 |
| 1176 | 2-(2,4-dihydroxyphenyl)-5,7-dihydroxy-3,8-bis(3-methylbut-2-enyl)chromen-4-one | 0.392884 | B(iii) | 474.711 | 421.165538 | NEG |  | 0.03 | 0.03 | 0.08 |
| 1177 | asebogenin | 0.392068308 | B(iii) | 480.331 | 289.106613 | POS | Flavonoids | 0.12 | 0.20 | 0.00 |
| 1178 | persicogenin 3'-glucoside | 0.389653923 | B(iii) | 400.662 | 479.1478575 | POS | Flavonoids | 81.61 | 53.25 | 1.94 |
| 1179 | tingenone | 0.389362077 | B(iii) | 359.6915 | 421.2785745 | POS | Triterpenoids | 0.10 | 0.08 | 0.10 |
| 1180 | isosakuranetin-7-o-neohesperidoside | 0.388635115 | B(iii) | 300.181 | 593.1869024 | NEG | Flavonoids | 0.00 | 0.00 | 0.00 |
| 1181 | musanolone e | 0.388472077 | B(iii) | 281.9665 | 305.0784518 | POS | Terphenyls | 0.03 | 0.02 | 0.03 |
| 1182 | icaritin | 0.387629 | B(iii) | 376.482 | 387.1436791 | POS | Flavonoids | 0.49 | 0.45 | 0.57 |
| 1183 | 6,7,3',4'-tetrahydroxyflavanone | 0.386580885 | B(iii) | 264.308 | 287.0557303 | NEG | Flavonoids | 0.00 | 0.01 | 0.00 |
| 1184 | 3'-o-methylviolanone | 0.385248077 | B(iii) | 453.9745 | 329.1023401 | NEG | Isoflavonoids | 0.12 | 2.79 | 12.25 |
| 1185 | medicagenic acid | 0.384853962 | B(iii) | 501.557 | 525.320994 | POS | Triterpenoids | 0.02 | 0.10 | 0.03 |
| 1186 | sibiricaxanthone b | 0.382883269 | B(iii) | 189.765 | 561.1217776 | POS | Xanthones | 0.00 | 0.00 | 0.00 |
| 1187 | methyl 3-ethenyl-4-[[6-[2-(4-hydroxyphenyl)ethyl]-4-oxooxan-2-yl]methyl]-2-[3,4,5-trihydroxy-6-(hydroxymethyl)oxan-2-yl]oxy-3,4-dihydro-2~{h}-pyran-5-carboxylate | 0.381961385 | B(iii) | 322.4815 | 623.2346232 | NEG |  | 0.07 | 0.02 | 0.03 |
| 1188 | physalin e | 0.381040154 | B(iii) | 411.03 | 545.2150497 | POS | Steroids | 4.20 | 4.42 | 2.15 |
| 1189 | 15-formyl-16-hydroxy-1,2,14,17,17-pentamethyl-8-(prop-1-en-2-yl)pentacyclo[11.7.0.0,.0,.0,icosane-5-carboxylic acid | 0.377322385 | B(iii) | 686.006 | 471.3455737 | POS |  | 0.05 | 0.17 | 0.04 |
| 1190 | 6-deoxodolichosterone | 0.377174538 | B(iii) | 629.422 | 449.3607223 | POS | Steroids | 1.19 | 1.14 | 0.12 |
| 1191 | uvarigranol c | 0.375695692 | B(iii) | 377.637 | 435.1426608 | POS | Phenolic acids | 0.23 | 1.58 | 0.90 |
| 1192 | robinin | 0.374810615 | B(iii) | 381.8295 | 741.2294145 | POS | Flavonoids | 0.02 | 0.05 | 0.00 |
| 1193 | [8]-paradol | 0.374378846 | B(iii) | 521.09 | 307.2260874 | POS | Aromatic polyketides | 0.40 | 0.51 | 1.03 |
| 1194 | santin | 0.374348077 | B(iii) | 344.699 | 343.0811673 | NEG | Flavonoids | 0.00 | 0.02 | 0.01 |
| 1195 | 15-methyl palmitic acid | 0.374334923 | B(iii) | 691.8245 | 269.248081 | NEG | Fatty Acids and Conjugates | 0.88 | 0.95 | 0.65 |
| 1196 | alpha-santonin | 0.371947538 | B(iii) | 467.886 | 269.1170063 | POS | Sesquiterpenoids | 0.44 | 0.30 | 1.01 |
| 1197 | glutamylleucine | 0.371872808 | B(iii) | 184.423 | 261.1440363 | POS | Small peptides | 0.02 | 0.02 | 0.03 |
| 1198 | rhapontin | 0.370832962 | B(iii) | 268.677 | 421.1463989 | POS | Stilbenoids | 0.04 | 0.13 | 0.01 |
| 1199 | 4'-demethylpodophyllotoxin | 0.369673385 | B(iii) | 267.8075 | 423.1061516 | POS | Lignans | 0.00 | 0.01 | 0.00 |
| 1200 | annosquamosin b | 0.367670385 | B(iii) | 628.8365 | 309.2413837 | POS | Diterpenoids | 0.19 | 0.68 | 0.12 |
| 1201 | dendrophenol | 0.366675615 | B(iii) | 381.558 | 305.1377624 | POS | Stilbenoids | 0.36 | 0.23 | 0.05 |
| 1202 | flavokawain a | 0.365981577 | B(iii) | 413.5415 | 315.1216702 | POS | Flavonoids | 1.76 | 3.18 | 0.39 |
| 1203 | isosakuranetin-7-o-rutinoside | 0.365922115 | B(iii) | 323.161 | 639.2051726 | NEG | Flavonoids | 0.00 | 0.00 | 0.00 |
| 1204 | brosimacutin f | 0.365756538 | B(iii) | 374.99 | 357.1321474 | POS | Flavonoids | 0.44 | 0.25 | 1.40 |
| 1205 | geniposide (not validated) | 0.363179577 | B(iii) | 203.9645 | 433.1304939 | NEG | Monoterpenoids | 0.00 | 0.00 | 0.00 |
| 1206 | 2'-methoxyflavone | 0.363046846 | B(iii) | 454.6225 | 251.0707652 | NEG | Flavonoids | 0.04 | 0.23 | 0.00 |
| 1207 | naringenin-7-o-rutinoside | 0.361924769 | B(iii) | 677.119 | 581.1788087 | POS | Flavonoids | 0.13 | 0.65 | 0.10 |
| 1208 | trigofoenoside a | 0.360814692 | B(iii) | 335.8435 | 903.4743037 | POS | Steroids | 0.06 | 0.02 | 0.09 |
| 1209 | holostyligone, (-)- | 0.360447846 | B(iii) | 417.4285 | 379.1533555 | POS | Lignans | 0.65 | 0.22 | 0.12 |
| 1210 | prim-o-glucosylcimifugin | 0.357470385 | B(iii) | 275.381 | 491.1523804 | POS | Chromanes | 0.01 | 0.00 | 0.00 |
| 1211 | 7,8-dihydroxycoumarin | 0.356881385 | B(iii) | 222.5715 | 177.0190449 | NEG | Coumarins | 0.01 | 0.00 | 0.00 |
| 1212 | (+)-pinoresinol | 0.355481615 | B(iii) | 350.015 | 359.148093 | POS | Lignans | 0.15 | 0.09 | 0.03 |
| 1213 | 14,15beta-dihydroxyklaineanone | 0.355152692 | B(iii) | 320.158 | 419.1683915 | POS | Triterpenoids | 0.12 | 0.05 | 0.03 |
| 1214 | rehmannioside a | 0.351881077 | B(iii) | 493.49 | 525.1908983 | POS | Monoterpenoids | 2.02 | 1.29 | 0.58 |
| 1215 | (3s,7r)-iso-jasmonic acid | 0.350910538 | B(iii) | 315.36 | 211.1323487 | POS | Octadecanoids | 0.06 | 0.04 | 0.02 |
| 1216 | cornuside | 0.350900538 | B(iii) | 317.4085 | 565.1456614 | POS | Monoterpenoids | 0.02 | 0.01 | 0.00 |
| 1217 | galangin | 0.348088538 | B(iii) | 323.078 | 269.0452158 | NEG | Flavonoids | 0.16 | 0.13 | 0.22 |
| 1218 | kaempferol 3-p-coumarate | 0.347209231 | B(iii) | 265.995 | 433.0893573 | POS | Flavonoids | 0.03 | 0.01 | 0.02 |
| 1219 | zingiberensis saponin i | 0.346519923 | B(iii) | 305.326 | 1047.535768 | POS | Steroids | 0.02 | 0.08 | 0.01 |
| 1220 | eriodictyol 5-methyl ether 7-o-xylosyl-(1->4)-arabinoside | 0.344964692 | B(iii) | 427.351 | 567.1760977 | POS | Flavonoids | 8.52 | 3.56 | 13.63 |
| 1221 | paeonol | 0.343551385 | B(iii) | 367.118 | 167.0696859 | POS |  | 0.45 | 0.24 | 0.33 |
| 1222 | multigilin | 0.341744231 | B(iii) | 307.689 | 361.1638267 | POS | Sesquiterpenoids | 0.33 | 0.18 | 0.01 |
| 1223 | camellianin a | 0.339687731 | B(iii) | 310.2165 | 621.1786621 | POS | Flavonoids | 0.21 | 0.05 | 0.09 |
| 1224 | filiforminol | 0.332868077 | B(iii) | 44.4504 | 311.0639486 | POS | Sesquiterpenoids | 0.03 | 0.03 | 0.04 |
| 1225 | baohuoside v | 0.325165585 | B(iii) | 442.321 | 809.2927479 | POS | Flavonoids | 0.91 | 0.74 | 0.80 |
| 1226 | lariciresinol | 0.325051692 | B(iii) | 314.152 | 359.1496607 | NEG | Lignans | 0.03 | 0.06 | 0.02 |
| 1227 | brazilin | 0.320077115 | B(iii) | 301.98 | 309.0726089 | POS | Isoflavonoids | 0.02 | 0.04 | 0.03 |
| 1228 | gomisin c | 0.314338769 | B(iii) | 387.477 | 559.1962924 | POS | Lignans | 0.40 | 0.81 | 0.39 |
| 1229 | bavachinin | 0.313237231 | B(iii) | 487.543 | 361.1422373 | POS | Flavonoids | 0.12 | 0.06 | 0.34 |
| 1230 | evodol | 0.312186808 | B(iii) | 328.2755 | 485.1791248 | POS | Triterpenoids | 0.32 | 1.42 | 0.03 |
| 1231 | brosimacutin c | 0.306418615 | B(iii) | 520.4265 | 343.1525352 | POS | Flavonoids | 0.05 | 0.11 | 0.01 |
| 1232 | grayanotoxin i | 0.304764538 | B(iii) | 520.443 | 451.2100538 | POS | Diterpenoids | 0.11 | 0.20 | 0.17 |
| 1233 | swertiajaponin | 0.304572269 | B(iii) | 240.704 | 463.1196321 | POS | Flavonoids | 0.01 | 0.01 | 0.01 |
| 1234 | 10-methylacridone | 0.303824923 | B(iii) | 368.4675 | 210.0837032 | POS | Anthranilic acid alkaloids | 0.05 | 0.02 | 0.03 |
| 1235 | 1,7,7-trimethylbicyclo[2.2.1]hept-2-yl 4-hydroxy-3-methoxybenzoate | 0.301991346 | B(iii) | 585.694 | 303.1619903 | NEG | Phenolic acids | 0.01 | 0.01 | 0.01 |
| 1236 | grossamide or its isomer (not validated) | 0.301957538 | B(iii) | 370.214 | 623.2345539 | NEG | Lignans | 0.04 | 0.07 | 0.01 |
| 1237 | eriodictyol-7-neohesperidoside | 0.301013577 | B(iii) | 336.5875 | 595.1652047 | NEG | Flavonoids | 0.01 | 0.00 | 0.02 |

**Table S3** Differential metabolites of flavonoids and stilbenoids of *D. cochinchinensis* from different origins

| chemical composition | Class | MEAN Gdc | MEAN Hdsp | Difference multiple |
| --- | --- | --- | --- | --- |
| 2-(3,4-dimethoxyphenyl)-5,6,7,8-tetramethoxy-4h-chromen-4-one | Flavonoids | 0.01 | 0.02 | 3.69 |
| oppositin | Flavonoids | 0.02 | 0.43 | 17.77 |
| kuwanone g | Flavonoids | 0.02 | 0.09 | 5.38 |
| 7-hydroxy-5,8,2'-trimethoxyflavanone | Flavonoids | 0.12 | 0.26 | 2.21 |
| hispidulin 7-glucoside | Flavonoids | 0.06 | 0.19 | 3.21 |
| hexamethylquercetagetin | Flavonoids | 0.17 | 0.33 | 1.90 |
| santin | Flavonoids | 0.00 | 0.02 | 5.47 |
| 5,7-dihydroxy-2-(4-hydroxyphenyl)-6-[(2s,3r,4r,5s,6r)-3,4,5-trihydroxy-6-(hydroxymethyl)oxan-2-yl]-8-[(2s,3r,4r,5r,6s)-3,4,5-trihydroxy-6-methyloxan-2-yl]chromen-4-one | Flavonoids | 0.81 | 0.34 | 2.36 |
| benzophenone | Flavonoids | 0.03 | 0.02 | 1.65 |
| dihydroisomilletenone methyl ether | Flavonoids | 0.18 | 0.08 | 2.18 |
| dihydropashanone | Flavonoids | 0.02 | 0.01 | 1.47 |
| spinoflavanone a | Flavonoids | 1.61 | 0.12 | 13.17 |
| 7-hydroxyflavone | Flavonoids | 0.00 | 0.00 | 2.38 |
| anhydroicaritin | Flavonoids | 0.20 | 0.04 | 5.40 |
| camellianin a | Flavonoids | 0.21 | 0.05 | 4.03 |
| aspalathin | Flavonoids | 0.03 | 0.00 | 7.05 |
| pinocembrin 7-o-benzoate | Flavonoids | 0.31 | 0.12 | 2.51 |
| kushenol c | Flavonoids | 0.12 | 0.02 | 6.10 |
| 5,7-dihydroxyflavanone | Flavonoids | 1.49 | 0.61 | 2.44 |
| chrysin | Flavonoids | 1.56 | 0.88 | 1.77 |
| moghanin a | Flavonoids | 10.41 | 5.12 | 2.03 |
| 2,4,4'-trihydroxydihydrochalcone | Flavonoids | 0.99 | 0.44 | 2.25 |

**Table S4** Differential metabolites of flavonoids and stilbenoids between *D. cochinchinensis* and *D. cambodiana*

| chemical composition | Class | MEAN Hdsp | MEAN Hdca | Difference multiple |
| --- | --- | --- | --- | --- |
| bavachinin | Flavonoids | 0.06 | 0.34 | 5.77 |
| naringin | Flavonoids | 0.00 | 0.12 | 41.29 |
| hesperidin | Flavonoids | 0.01 | 0.03 | 2.23 |
| eriodictyol 5-methyl ether 7-o-xylosyl-(1->4)-arabinoside | Flavonoids | 3.56 | 13.63 | 3.83 |
| 2',4'-dihydroxy-4-methoxychalcone | Flavonoids | 12.74 | 38.66 | 3.03 |
| hispidulin | Flavonoids | 0.76 | 1.13 | 1.48 |
| isosakuranetin-7-o-neohesperidoside | Flavonoids | 0.00 | 0.00 | 2.95 |
| rhamnazin 3-rutinoside | Flavonoids | 0.07 | 0.18 | 2.67 |
| sinensetin | Flavonoids | 0.01 | 0.03 | 2.24 |
| 2'-hydroxy-3',4',6',3,4-pentamethoxychalcone | Flavonoids | 0.21 | 0.77 | 3.57 |
| angophorol | Flavonoids | 0.10 | 0.66 | 6.80 |
| citromitin | Flavonoids | 0.43 | 0.51 | 1.20 |
| 1-[2,4-dihydroxy-6-[(2s,3r,4s,5s,6r)-3,4,5-trihydroxy-6-(hydroxymethyl)oxan-2-yl]oxyphenyl]-3-(4-methoxyphenyl)propan-1-one | Flavonoids | 0.00 | 0.03 | 6.82 |
| brosimacutin f | Flavonoids | 0.25 | 1.40 | 5.54 |
| eriodictyol-7-neohesperidoside | Flavonoids | 0.00 | 0.02 | 6.30 |
| sanggenol l | Flavonoids | 1.40 | 0.02 | 64.84 |
| 6-hydroxy-2-(4-methoxyphenyl)-4h-chromen-4-one | Flavonoids | 10.70 | 0.67 | 15.99 |
| 3'-methyl-2',4',6'-trihydroxydihydrochalcone | Flavonoids | 0.41 | 0.11 | 3.61 |
| 7,3'-dihydroxy-4'-methoxy-8-methylflavan | Flavonoids | 0.94 | 0.31 | 3.05 |
| (2z)-6-hydroxy-2-[(4-hydroxy-3-methoxyphenyl)methylidene]-1-benzofuran-3-one | Flavonoids | 1.71 | 0.47 | 3.65 |
| salicifolioside a | Flavonoids | 0.85 | 0.18 | 4.86 |
| 3',4'-dihydroxy-5,7-dimethoxyflavan | Flavonoids | 1.59 | 0.45 | 3.53 |
| 5,4'-dihydroxy-6,7-dimethoxyflavanone | Flavonoids | 0.77 | 0.24 | 3.20 |
| 2-methoxy-4-hydroxydihydrochalcone | Flavonoids | 1.81 | 0.66 | 2.72 |
| artonol c | Flavonoids | 1.07 | 0.04 | 28.90 |
| 6-methoxy-[2'',3'':7,8]furanoflavanone | Flavonoids | 0.56 | 0.10 | 5.66 |
| (2s)-4'-hydroxy-5,7,3'-trimethoxyflavan | Flavonoids | 239.53 | 4.27 | 56.11 |
| flavone base + 2o, 2meo, c-hex | Flavonoids | 0.07 | 0.01 | 8.52 |
| 7-methoxyflavonol | Flavonoids | 107.75 | 3.71 | 29.06 |
| 7-hydroxy-5,4'-dimethoxyflavan | Flavonoids | 0.29 | 0.16 | 1.78 |
| brosimacutin i | Flavonoids | 0.09 | 0.01 | 12.12 |
| 5,7-dihydroxy-2'-methoxyflavone | Flavonoids | 2.99 | 0.46 | 6.44 |
| nicotiflorin | Flavonoids | 0.01 | 0.00 | 2.35 |
| lethedoside b | Flavonoids | 1.45 | 0.23 | 6.43 |
| 6,7,3',4'-tetrahydroxyflavanone | Flavonoids | 0.01 | 0.00 | 2.96 |
| 2',4',6'-trihydroxy-4-methoxydihydrochalcone | Flavonoids | 1.54 | 0.84 | 1.84 |
| karanjin | Flavonoids | 0.40 | 0.17 | 2.31 |
| oroxylin a | Flavonoids | 1.76 | 0.21 | 8.28 |
| farrerol | Flavonoids | 2.75 | 0.06 | 42.97 |
| 5,7-dihydroxy-2-(4-hydroxyphenyl)-8-[3,4,5-trihydroxy-6-(hydroxymethyl)oxan-2-yl]-6-(3,4,5-trihydroxyoxan-2-yl)chromen-4-one | Flavonoids | 0.03 | 0.00 | 16.54 |
| 7-hydroxy-2-(4-hydroxy-3,5-dimethoxyphenyl)-5-[(2s,3r,4s,5s,6r)-3,4,5-trihydroxy-6-(hydroxymethyl)oxan-2-yl]oxychromen-4-one | Flavonoids | 1.00 | 0.03 | 36.63 |
| (+)-8-hydroxy-5,5-dimethylpeltogynan | Flavonoids | 0.30 | 0.16 | 1.83 |
| sempervirenoside b | Flavonoids | 0.16 | 0.00 | 45.18 |
| phloridzin | Flavonoids | 0.00 | 0.00 | 2.10 |
| robinin | Flavonoids | 0.05 | 0.00 | 125.18 |
| didymin | Flavonoids | 0.36 | 0.02 | 14.95 |
| 3-[(2s,3r,4s,5r,6r)-3,5-dihydroxy-6-(hydroxymethyl)-4-[(2s,3r,4r,5r,6s)-3,4,5-trihydroxy-6-methyloxan-2-yl]oxyoxan-2-yl]oxy-5,7-dihydroxy-2-(4-hydroxyphenyl)chromen-4-one | Flavonoids | 0.02 | 0.00 | 4.07 |
| angoletin | Flavonoids | 5.41 | 0.17 | 32.03 |
| 2'-methoxyflavone | Flavonoids | 0.23 | 0.00 | 1187.54 |
| typhaneoside | Flavonoids | 0.14 | 0.02 | 5.76 |
| cardamonin | Flavonoids | 1.01 | 0.08 | 12.08 |
| flavokawain a | Flavonoids | 3.18 | 0.39 | 8.07 |
| fisetinidol | Flavonoids | 0.28 | 0.09 | 3.25 |
| piceid | Stilbenoids | 0.02 | 0.03 | 1.70 |
| trans-resveratrol | Stilbenoids | 30.65 | 0.16 | 195.44 |
| pinosylvin | Stilbenoids | 4.13 | 0.00 | 1337.71 |
| 3,4,5-trihydroxystilbene | Stilbenoids | 0.07 | 0.00 | 58.93 |
| resveratrol 4'-methyl ether | Stilbenoids | 0.05 | 0.02 | 2.86 |
| e-resveratrol trimethyl ether | Stilbenoids | 0.16 | 0.05 | 3.37 |
| oxyresveratrol | Stilbenoids | 0.03 | 0.02 | 1.79 |
| cis-mulberroside a | Stilbenoids | 1.23 | 0.45 | 2.76 |
| combrestatin a4 | Stilbenoids | 0.60 | 0.10 | 5.74 |

**Table S5** UHPLC-PDA gradient elution schemes

| Scheme (number) | time（min） | The proportion of mobile phase A,% | The proportion of mobile phase B,% |
| --- | --- | --- | --- |
| 1 | 0 | 28 | 72 |
|  | 20 | 38 | 62 |
|  | 50 | 48 | 52 |
|  | 59 | 95 | 5 |
|  | 60 | 28 | 72 |
| 2 | 0 | 25 | 75 |
|  | 75 | 50 | 50 |
|  | 90 | 95 | 5 |
| 3 | 0 | 23 | 77 |
|  | 80 | 43 | 57 |
| 4 | 0 | 20 | 80 |
|  | 5 | 25 | 75 |
|  | 30 | 30 | 70 |
|  | 62 | 38 | 64 |
|  | 75 | 95 | 5 |
|  | 76 | 20 | 80 |

**Table S6** Quantitative analytical results of the 12 analytes in 51 batches CDB samples (mg/g,n=3)

| Sample Code | Compounds content | | | | | | | | | | | |
| --- | --- | --- | --- | --- | --- | --- | --- | --- | --- | --- | --- | --- |
|  | X_1_ | X_2_ | X_3_ | X_4_ | X_5_ | X_6_ | X_7_ | X_8_ | X_9_ | X_10_ | X_11_ | X_12_ |
| Gdc-1 | 4.72 | 4.19 | 1.66 | 0.63 | 0.73 | 9.59 | 1.00 | 1.67 | 0.56 | 7.51 | 1.09 | 2.99 |
| Gdc-2 | 5.50 | 4.63 | 1.52 | 0.38 | 2.34 | 8.02 | 6.25 | 2.36 | <LOQ | 9.44 | 1.87 | 2.79 |
| Gdc-3 | 2.25 | 1.75 | 0.42 | 0.36 | 0.35 | 2.57 | 0.68 | 0.95 | 0.71 | 3.54 | 1.01 | 2.07 |
| Gdc-4 | 3.05 | 2.59 | 0.61 | 0.31 | 0.86 | 2.53 | 3.49 | 0.90 | <LOQ | 1.79 | 1.59 | 1.99 |
| Gdc-5 | 10.00 | 5.66 | 4.03 | 1.92 | 3.17 | 9.24 | 7.49 | 2.60 | <LOQ | 4.78 | 2.15 | 4.51 |
| Gdc-6 | 9.38 | 8.76 | 3.68 | 1.30 | 4.01 | 21.05 | 3.46 | 4.03 | <LOQ | 4.05 | 2.61 | 5.90 |
| Gdc-7 | 5.61 | 4.42 | 0.52 | 0.40 | 1.67 | 3.89 | 1.97 | 2.81 | <LOQ | 1.18 | 0.88 | 5.10 |
| Gdc-8 | 3.07 | 2.92 | 0.59 | 0.44 | 0.43 | 3.75 | 0.49 | 1.42 | <LOQ | 10.67 | 3.94 | 7.60 |
| Gdc-9 | 2.21 | 3.62 | 0.39 | 0.25 | 0.16 | 1.23 | 0.05 | 0.64 | 0.10 | 2.58 | 1.21 | 4.18 |
| Gdc-10 | 5.15 | 17.87 | 0.64 | 0.69 | 0.90 | 7.87 | 1.49 | 3.13 | 0.22 | 4.44 | 1.42 | 9.00 |
| Gdc-11 | 4.59 | 4.00 | 2.43 | 1.08 | 1.11 | 5.38 | 1.14 | 1.29 | 0.78 | 3.28 | 2.49 | 5.49 |
| Gdc-12 | 3.00 | 2.19 | 0.62 | 0.33 | 0.24 | 2.12 | 0.34 | 0.96 | 0.36 | 1.09 | 1.77 | 3.53 |
| Gdc-13 | 4.65 | 4.09 | 0.67 | 0.19 | 0.45 | 2.40 | 0.90 | 1.83 | <LOQ | 1.74 | 1.41 | 7.94 |
| Gdc-14 | 3.51 | 10.60 | 0.74 | 0.46 | 0.54 | 3.41 | 0.93 | 2.77 | 0.36 | 2.33 | 1.29 | 5.75 |
| Gdc-15 | 4.93 | 6.41 | 4.10 | 5.62 | 0.97 | 9.17 | 3.27 | 4.59 | <LOQ | 3.36 | 3.44 | 9.07 |
| Gdc-16 | 2.33 | 1.63 | 0.45 | 0.35 | 0.42 | 1.38 | 0.51 | 0.52 | 0.36 | 1.76 | 1.21 | 1.34 |
| Gdc-17 | 2.07 | 2.81 | 0.39 | 0.42 | 0.44 | 3.22 | 0.46 | 0.91 | 0.17 | 3.60 | 2.78 | 2.23 |
| Gdc-18 | 3.07 | 2.41 | 1.32 | 0.46 | 0.49 | 2.36 | 0.81 | 1.06 | <LOQ | 4.99 | 7.06 | 4.29 |
| Gdc-19 | 4.40 | 0.94 | 0.52 | 0.20 | 0.34 | 0.76 | 0.08 | 0.25 | <LOQ | 0.34 | 6.42 | 1.14 |
| Gdc-20 | 2.81 | 3.35 | 0.26 | 0.37 | 0.18 | 2.26 | 0.26 | 0.83 | 0.15 | 3.56 | 3.27 | 8.85 |
| Gdc-21 | 2.85 | 2.61 | 0.48 | 0.43 | 0.53 | 2.27 | 0.33 | 0.71 | 0.47 | 3.30 | 4.26 | 3.14 |
| Gdc-22 | 3.55 | 5.37 | 0.41 | 0.31 | 1.35 | 6.38 | 0.56 | 2.70 | <LOQ | 2.68 | 2.23 | 7.44 |
| Gdc-23 | 5.29 | 9.45 | 2.39 | 1.14 | 1.96 | 6.96 | 1.11 | 4.85 | <LOQ | 3.11 | 2.52 | 8.98 |
| Gdc-24 | 3.04 | 3.50 | 0.41 | 0.26 | 0.44 | 1.25 | 2.48 | 1.29 | <LOQ | 2.73 | 0.83 | 1.97 |
| Gdc-25 | 5.76 | 8.82 | 1.23 | 0.58 | 2.28 | 8.29 | 4.84 | 5.80 | <LOQ | 4.08 | 8.59 | 16.24 |
| Gdc-26 | 3.99 | 2.20 | 0.98 | 0.49 | 1.03 | 3.08 | 2.62 | 1.60 | <LOQ | 2.37 | 3.88 | 3.22 |
| Gdc-27 | 2.43 | 1.70 | 0.42 | 0.32 | 0.41 | 1.41 | 0.31 | 0.42 | <LOQ | 1.14 | 3.73 | 1.38 |
| Gdc-28 | 3.15 | 2.26 | 0.80 | 0.40 | 1.20 | 1.26 | 3.38 | 1.64 | <LOQ | 0.48 | 1.55 | 2.82 |
| Gdc-29 | 3.72 | 2.20 | 0.58 | 0.31 | 0.93 | 2.86 | 2.21 | 1.03 | <LOQ | 3.86 | 2.34 | 1.62 |
| Gdc-30 | 3.36 | 3.66 | 0.84 | 0.66 | 1.63 | 3.16 | 2.46 | 2.66 | <LOQ | 0.97 | 4.90 | 4.09 |
| Gdc-31 | 3.93 | 8.04 | 1.02 | 0.24 | 1.42 | 9.32 | 5.22 | 3.09 | <LOQ | 1.86 | 2.65 | 3.00 |
| Hdsp-1 | 2.78 | 2.18 | 0.46 | 0.19 | 3.17 | 1.01 | 0.26 | 0.58 | 0.55 | 0.26 | 3.89 | 0.25 |
| Hdsp-2 | 2.71 | 2.04 | 0.49 | 0.21 | 1.70 | 1.55 | 0.28 | 1.30 | 0.66 | 1.14 | 3.33 | 0.45 |
| Hdsp-3 | 2.95 | 1.29 | 0.77 | 0.40 | 0.92 | 2.07 | 0.76 | 1.19 | 0.77 | 2.31 | 7.21 | 0.39 |
| Hdsp-4 | 3.54 | 6.58 | 0.60 | 0.38 | 4.91 | 4.76 | 1.78 | 3.75 | 1.76 | 2.03 | 11.59 | 1.80 |
| Hdsp-5 | 3.31 | 2.04 | 0.61 | 0.33 | 0.91 | 2.50 | 2.07 | 1.60 | 1.12 | 2.42 | 5.42 | 1.05 |
| Hdsp-6 | 4.89 | 4.59 | 1.09 | 0.77 | 1.38 | 4.81 | 0.68 | 0.94 | 1.02 | 3.46 | 5.52 | 1.50 |
| Hdsp-7 | 5.20 | 8.11 | 1.09 | 0.44 | 3.42 | 5.33 | 3.12 | 2.64 | 1.41 | 1.30 | 13.33 | 1.48 |
| Hdsp-8 | 4.46 | 11.98 | 1.42 | 1.29 | 9.28 | 8.17 | 3.26 | 4.91 | 4.09 | 3.56 | 14.97 | 2.84 |
| Hdsp-9 | 3.33 | 3.22 | 0.19 | 0.17 | 1.05 | 2.78 | 1.43 | 2.00 | 0.45 | 2.92 | 17.97 | 1.48 |
| Hdsp-10 | 2.51 | 2.48 | 0.48 | 0.25 | 1.30 | 1.25 | 2.00 | 1.33 | 0.75 | 0.80 | 1.20 | 1.21 |
| Hdsp-11 | 3.15 | 4.20 | 0.73 | 0.57 | 3.11 | 2.35 | 3.27 | 2.83 | 2.39 | 1.25 | 5.43 | 3.01 |
| Hdsp-12 | 2.91 | 2.02 | 0.61 | 0.27 | 2.68 | 1.55 | 1.66 | 1.48 | 1.35 | 0.58 | 1.86 | 0.96 |
| Hdca-1 | 2.08 | 0.36 | 0.08 | 0.09 | 0.14 | 0.68 | 0.12 | 0.03 | 0.20 | 0.45 | 0.10 | 0.10 |
| Hdca-2 | 2.38 | 0.63 | 0.20 | 0.18 | 0.26 | 2.88 | 0.20 | 0.13 | 0.24 | 1.23 | 0.14 | 0.25 |
| Hdca-3 | 3.57 | 1.05 | 0.57 | 0.43 | 0.52 | 2.46 | 0.39 | 0.22 | 0.64 | 1.45 | 0.31 | 0.22 |
| Hdca-4 | 1.50 | 0.31 | 1.21 | 0.35 | 0.39 | 5.24 | 0.43 | 0.25 | 0.04 | 0.03 | 0.65 | 0.37 |
| Hdca-5 | 1.69 | 0.12 | 0.16 | 0.41 | 0.50 | 2.15 | 0.08 | 0.06 | 0.18 | 0.09 | 0.10 | 0.09 |
| Hdca-6 | 1.93 | 0.42 | 0.80 | 0.34 | 0.80 | 4.09 | 0.17 | 0.10 | 0.02 | 0.01 | 0.20 | 0.24 |
| Hdca-7 | 1.32 | 0.45 | 0.20 | 0.14 | 0.22 | 1.18 | 0.12 | 0.08 | 0.05 | 0.04 | 1.69 | 0.06 |
| Hdca-8 | 1.86 | 0.24 | 0.48 | 0.17 | 0.33 | 0.96 | 0.19 | 0.08 | 0.07 | 0.21 | 0.43 | 0.11 |

**Note:**<LOQ indicated that the content was less than the quantitative limit.

**Table S7** The compound names represented by each serial number in Figure 3

| Gdc vs Hdsp | | Hdsp VS Hdca | |
| --- | --- | --- | --- |
| No. | chemical composition | No. | chemical composition |
| 1 | pro | 1 | rhein |
| 2 | multifidol 2-[apiosyl-(1->6)-glucoside] | 2 | plumieride |
| 3 | methysticin | 3 | bavachinin |
| 4 | methyl 3,4,5-trimethoxycinnamate | 4 | octadecanedioic acid |
| 5 | sinapic acid; plasma id-325 | 5 | hexadecanedioic acid |
| 6 | khellin | 6 | 6,7-dimethoxy-4-methylcoumarin |
| 7 | caftaric acid | 7 | 4-o-methylphloracetophenone |
| 8 | 2-(3,4-dimethoxyphenyl)-5,6,7,8-tetramethoxy-4h-chromen-4-one | 8 | naringin |
| 9 | triptonide | 9 | isoeugenitol |
| 10 | 5-[6-(3-hydroxy-4-methoxyphenyl)-1,3,3a,4,6,6a-hexahydrofuro[3,4-c]furan-3-yl]-2-methoxyphenol | 10 | paulownin |
| 11 | testosterone | 11 | eudesmin |
| 12 | arachidic acid | 12 | hesperidin |
| 13 | dulxanthone d | 13 | jaeschkeanadiol |
| 14 | 1,5,8-trihydroxy-3-methyl-2-prenylxanthone | 14 | eriodictyol 5-methyl ether 7-o-xylosyl-(1->4)-arabinoside |
| 15 | n6-(delta2-isopentenyl)-adenine | 15 | 13z-docosenamide |
| 16 | brazilein | 16 | 2-(2-oxo-8,9-dihydrofuro[2,3-h]chromen-8-yl)propan-2-yl acetate |
| 17 | paclitaxel | 17 | 2',4'-dihydroxy-4-methoxychalcone |
| 18 | methyl (1s,4as,7as)-4'-[(1s)-1-[(e)-3-(4-hydroxyphenyl)prop-2-enoyl]oxyethyl]-5'-oxo-1-[(2s,3r,4s,5s,6r)-3,4,5-trihydroxy-6-(hydroxymethyl)oxan-2-yl]oxyspiro[4a,7a-dihydro-1h-cyclopenta[c]pyran-7,2'-furan]-4-carboxylate | 18 | rubiadin |
| 19 | hastatoside | 19 | 10-methylacridone |
| 20 | (2~{s},3~{r},4~{s},5~{s},6~{r})-2-[4-[(1~{s},2~{s},3~{s})-3,7-dihydroxy-2,3-bis(hydroxymethyl)-6-methoxy-2,4-dihydro-1~{h}-naphthalen-1-yl]-2-methoxyphenoxy]-6-(hydroxymethyl)oxane-3,4,5-triol | 20 | dodecanamide |
| 21 | 7-methoxy-6-(1,2,3-trihydroxy-3-methylbutyl)chromen-2-one | 21 | 9z,12z-octadecadienal |
| 22 | tephrorianin | 22 | 8-dimethylallyllisetin |
| 23 | 3,4-di-o-caffeoylquinic acid | 23 | hispidulin |
| 24 | garciduol b | 24 | isosakuranetin-7-o-neohesperidoside |
| 25 | 1',2'-dihydro-2',6-dihydroxyrotenone | 25 | 5,6,7-trimethoxycoumarin |
| 26 | c26:1n-9 | 26 | boeravinone c |
| 27 | [(1as,1bs,2s,5ar,6s,6as)-1a-(hydroxymethyl)-2-[(2s,3r,4s,5s,6r)-3,4,5-trihydroxy-6-(hydroxymethyl)oxan-2-yl]oxy-2,5a,6,6a-tetrahydro-1bh-oxireno[5,6]cyclopenta[1,3-c]pyran-6-yl] benzoate | 27 | feruloyl hexoside (isomer of 849) |
| 28 | beta-glucose pentaacetic acid | 28 | 1-acetyl beta carboline |
| 29 | [3,4,5-trihydroxy-6-(hydroxymethyl)oxan-2-yl] 9-(hydroxymethyl)-2,2,6a,6b,9,12a-hexamethyl-10-[3,4,5-trihydroxy-6-[(3,4,5-trihydroxy-6-methyloxan-2-yl)oxymethyl]oxan-2-yl]oxy-1,3,4,5,6,6a,7,8,8a,10,11,12,13,14b-tetradecahydropicene-4a-carboxylate | 29 | phrymarolin i |
| 30 | oppositin | 30 | luvangetin |
| 31 | kuwanone g | 31 | mukoenine a |
| 32 | calomelanol h | 32 | rhamnazin 3-rutinoside |
| 33 | uvarigranol c | 33 | sinensetin |
| 34 | duartin (-) | 34 | oleuropein aglycon derivative |
| 35 | 5'-hydroxy-3'-methoxysativan | 35 | kaempferol 3-p-coumarate |
| 36 | propylparaben | 36 | citrusin |
| 37 | grayanotoxin i | 37 | 2'-hydroxy-3',4',6',3,4-pentamethoxychalcone |
| 38 | brosimacutin c | 38 | prim-o-glucosylcimifugin |
| 39 | 2'-hydroxy-3',4',6'-trimethoxydihydrochalcone | 39 | estrone |
| 40 | 3',4'-dihydroxy-7-methoxy-8-(3-methylbut-2-enyl)-2'''-(1-hydroxy-1-methylethyl)-furano-(4'',5'':6,5)favanone | 40 | dioscin |
| 41 | 7-hydroxy-5,8,2'-trimethoxyflavanone | 41 | hydroquinone |
| 42 | (r,e)-8,8-dimethyl-2-oxo-2,8,9,10-tetrahydropyrano[2,3-f]chromen-9-yl 2-methylbut-2-enoate | 42 | polyphyllin a |
| 43 | kushenol f | 43 | eugenitin |
| 44 | lotisoflavan | 44 | 1,3,6-trihydroxy-2-(3-methylbut-2-enyl)xanthen-9-one |
| 45 | coniferyl alcohol | 45 | angophorol |
| 46 | 3,7-dimethoxyflavone | 46 | piceid |
| 47 | asebogenin | 47 | diosgenin |
| 48 | 4'-hydroxyacetophenone 4'-[4-hydroxy-3,5-dimethoxybenzoyl-(->5)-apiosyl-(1->2)-glucoside] | 48 | 7-hydroxy-3-(3-hydroxy-4-methoxybenzyl)-5-methoxy-4-chromanone |
| 49 | 2-[2-(3,4-dimethoxyphenyl)ethyl]-4-methoxy-2,3-dihydropyran-6-one | 49 | histamine |
| 50 | isosclerone | 50 | anofinic acid |
| 51 | eudesmic acid | 51 | glyasperin c |
| 52 | (2~{r},3~{s},4~{s},5~{r},6~{r})-2-[[(2~{r},3~{r},4~{r})-3,4-dihydroxy-4-(hydroxymethyl)oxolan-2-yl]oxymethyl]-6-phenylmethoxyoxane-3,4,5-triol | 52 | 3,4,5-trimethoxycinnamic aldehyde |
| 53 | ethyl vanillin | 53 | resorcinol |
| 54 | (2s,3r,4r,5r,6s)-2-[(2r,3r,4s,5r,6r)-2-[2-(3,4-dihydroxyphenyl)ethoxy]-3,5-dihydroxy-6-(hydroxymethyl)oxan-4-yl]oxy-6-methyloxane-3,4,5-triol | 54 | (2s,3r)-3-hydroxy-4-methoxy-2-(2-phenylethyl)-2,3-dihydropyran-6-one |
| 55 | ferulic acid | 55 | (3~{r},5~{s},8~{e})-5,9,14-trimethyl-4,12-dioxatricyclo[9.3.0.0^{3,5}]tetradeca-1(11),8,13-trien-2-one |
| 56 | (3r)-3-(3,4-dimethoxyphenyl)-8-hydroxy-3,4-dihydroisochromen-1-one | 56 | citromitin |
| 57 | oxypinnatanine | 57 | syringic acid |
| 58 | evodol | 58 | pachyrrhizin |
| 59 | d-mannitol | 59 | pomiferin |
| 60 | harmine | 60 | rhodomyrtoxin b |
| 61 | sorbitol | 61 | scutellarioside ii |
| 62 | hispidulin 7-glucoside | 62 | (1s,4as,7s,7as)-7-hydroxy-7-methyl-1-[(2s,3r,4s,5s,6r)-3,4,5-trihydroxy-6-(hydroxymethyl)oxan-2-yl]oxy-4a,5,6,7a-tetrahydro-1h-cyclopenta[c]pyran-4-carboxylic acid |
| 63 | adipic acid | 63 | gambiriin b3 |
| 64 | 1,5-anhydro-glucitol | 64 | garcinone d |
| 65 | (9a-hydroxy-3,8a-dimethyl-5-methylidene-2-oxo-4,4a,6,7,8,9-hexahydrobenzo[f][1]benzofuran-8-yl) acetate | 65 | 1-[2,4-dihydroxy-6-[(2s,3r,4s,5s,6r)-3,4,5-trihydroxy-6-(hydroxymethyl)oxan-2-yl]oxyphenyl]-3-(4-methoxyphenyl)propan-1-one |
| 66 | garciduol a | 66 | phenylacetic acid + 2o, o-hex |
| 67 | 5,7-dihydroxy-2-phenyl-6-[3,4,5-trihydroxy-6-(hydroxymethyl)oxan-2-yl]-8-(3,4,5-trihydroxyoxan-2-yl)chromen-4-one | 67 | trigofoenoside a |
| 68 | calycosin | 68 | physalin b |
| 69 | tangeritin | 69 | brosimone g |
| 70 | 5,7,8-trimethoxyflavanone | 70 | dioctyl hexanedioate |
| 71 | glycitin | 71 | 5-o-methylembelin |
| 72 | 2-methoxyhomopterocarpin | 72 | [(1s,3ar,5r,5ar,8ar,9s,9ar)-1,5,8a-trimethyl-2,8-dioxo-3a,4,5,5a,9,9a-hexahydro-1h-azuleno[6,5-b]furan-9-yl] (z)-2-methylbut-2-enoate |
| 73 | gibberellic acid | 73 | sterebin a |
| 74 | [2-[(2~{s},3~{r},4~{s},5~{s},6~{r})-3,4,5-trihydroxy-6-(hydroxymethyl)oxan-2-yl]oxyphenyl]methyl 2-hydroxy-6-[(2~{s},3~{r},4~{s},5~{s},6~{r})-3,4,5-trihydroxy-6-(hydroxymethyl)oxan-2-yl]oxybenzoate | 74 | corchorifatty acid f |
| 75 | hydrocortisone | 75 | methyl 3-(3-methylbut-2-enyl)-1,4-bis[[3,4,5-trihydroxy-6-(hydroxymethyl)oxan-2-yl]oxy]naphthalene-2-carboxylate |
| 76 | histidine | 76 | lapidin |
| 77 | dehydrodihydrorotenone | 77 | (1r,4r,6r,10s)-4,12,12-trimethyl-9-methylene-5-oxatricyclo[8.2.0.0~4,6~]dodecane |
| 78 | pratol | 78 | villol |
| 79 | ethyl 3-(methylthio)propanoate | 79 | 1',2'-dihydro-2',6-dihydroxyrotenone |
| 80 | (-)-mucronulatol | 80 | (2~{r},3~{r})-2-(3,4-dihydroxyphenyl)-5,7-dihydroxy-3-[(2~{s},3~{r},4~{s},5~{s},6~{r})-3,4,5-trihydroxy-6-(hydroxymethyl)oxan-2-yl]oxy-2,3-dihydrochromen-4-one |
| 81 | 3,5-dimethoxyphenol | 81 | butyramide |
| 82 | 4-acetamidobutanoate | 82 | 3-(4-hydroxy-2-methoxyphenyl)-2-propenal |
| 83 | (e)-3-(2-hydroxyphenyl)-2-propenal | 83 | pinoresinol dimethyl ether |
| 84 | 3-hydroxyphenylacetic acid | 84 | brosimacutin f |
| 85 | (-)-gallocatechin | 85 | coniferyl aldehyde |
| 86 | trans-cinnamic acid | 86 | licarin a |
| 87 | fragransol b | 87 | garcilivin b |
| 88 | isoamericanin a | 88 | (1e,4z,6e)-5-hydroxy-1,7-bis(4-hydroxy-3-methoxyphenyl)hepta-1,4,6-trien-3-one |
| 89 | 5,7-dimethoxy-2-phenyl-4h-chromen-4-one | 89 | 3-aminoisobutyric acid |
| 90 | crotonoside | 90 | eriodictyol-7-neohesperidoside |
| 91 | norlichexanthone | 91 | p-anisaldehyde |
| 92 | maltose | 92 | isosativan |
| 93 | hexamethylquercetagetin | 93 | sanggenol l |
| 94 | schisandrin c | 94 | veratric acid |
| 95 | tomatine | 95 | verimol a |
| 96 | (+)-dihydrowighteone | 96 | 6-hydroxy-2-(4-methoxyphenyl)-4h-chromen-4-one |
| 97 | lucuminic acid | 97 | 3-(2-hydroxy-4-methoxyphenyl)-3,4-dihydro-2h-1-benzopyran-7-ol |
| 98 | epiafzelechin (2r,3r)(-) | 98 | 3'-methyl-2',4',6'-trihydroxydihydrochalcone |
| 99 | methyl 3-ethenyl-4-(2-oxoethyl)-2-[3,4,5-trihydroxy-6-(hydroxymethyl)oxan-2-yl]oxy-3,4-dihydro-2~{h}-pyran-5-carboxylate | 99 | 7,3'-dihydroxy-4'-methoxy-8-methylflavan |
| 100 | spirostane-3,6-dione | 100 | 1-phenylethyl formate |
| 101 | sn-glycero-3-phosphocholine | 101 | magnolin |
| 102 | santin | 102 | e-resveratrol trimethyl ether |
| 103 | isobrucein a | 103 | (2z)-6-hydroxy-2-[(4-hydroxy-3-methoxyphenyl)methylidene]-1-benzofuran-3-one |
| 104 | artoindonesianin a | 104 | daidzein 7-o-apiosyl-(1->6)-glucoside |
| 105 | epimedoside c | 105 | guanine; ce30; uytpupdqbnuygx-uhfffaoysa-n |
| 106 | triacetin (c2:0) | 106 | guanosine |
| 107 | na-methylhistamine | 107 | 2-methoxycinnamic acid |
| 108 | angoroside a | 108 | multifidol 2-[apiosyl-(1->6)-glucoside] |
| 109 | beta-sinensal | 109 | beta-hydroxymyristic acid |
| 110 | leonoside b | 110 | p-anisic acid |
| 111 | allothreonine | 111 | oxyresveratrol |
| 112 | 2-[[3,4-dihydroxy-4-(hydroxymethyl)oxolan-2-yl]oxymethyl]-6-[(5-hydroxy-1,7,7-trimethyl-2-bicyclo[2.2.1]heptanyl)oxy]oxane-3,4,5-triol | 112 | (r)-(+)-1,2-dithiolane-3-pentanoic acid |
| 113 | (2~{r},3~{r},4~{s},5~{s},6~{r})-2-[2-(4-methoxyphenyl)ethoxy]-6-[[(2~{s},3~{r},4~{s},5~{s})-3,4,5-trihydroxyoxan-2-yl]oxymethyl]oxane-3,4,5-triol | 113 | pro |
| 114 | 1-acetyl beta carboline | 114 | mesaconitine |
| 115 | 3,4-dimethyl-2,5-bis(3,4,5-trimethoxyphenyl)oxolane | 115 | reynosin |
| 116 | daidzein 7-o-apiosyl-(1->6)-glucoside | 116 | dopamine [m+h-nh2]+; aif; ce0; ms2dec |
| 117 | plumieride | 117 | asebogenin |
| 118 | ureidosuccinic acid | 118 | 2-[2-(3,4-dimethoxyphenyl)ethyl]-4-methoxy-2,3-dihydropyran-6-one |
| 119 | pinoresinol dimethyl ether | 119 | lotisoflavan |
| 120 | glyasperin d | 120 | 4'-hydroxyacetophenone 4'-[4-hydroxy-3,5-dimethoxybenzoyl-(->5)-apiosyl-(1->2)-glucoside] |
| 121 | 5,7-dihydroxy-2-(4-hydroxyphenyl)-6-[(2s,3r,4r,5s,6r)-3,4,5-trihydroxy-6-(hydroxymethyl)oxan-2-yl]-8-[(2s,3r,4r,5r,6s)-3,4,5-trihydroxy-6-methyloxan-2-yl]chromen-4-one | 121 | coniferyl alcohol |
| 122 | benzophenone | 122 | (2~{s},3~{r},4~{s},5~{s},6~{r})-2-[4-[(1~{s},2~{s},3~{s})-3,7-dihydroxy-2,3-bis(hydroxymethyl)-6-methoxy-2,4-dihydro-1~{h}-naphthalen-1-yl]-2-methoxyphenoxy]-6-(hydroxymethyl)oxane-3,4,5-triol |
| 123 | italidipyrone | 123 | kushenol f |
| 124 | (5s)-5-hydroxy-1,7-diphenylheptan-3-one | 124 | choline; ce10; oeyiohpdsnjkls-uhfffaoysa-n |
| 125 | tutin | 125 | isokobusone |
| 126 | guanine; ce30; uytpupdqbnuygx-uhfffaoysa-n | 126 | alpha-cyperone |
| 127 | haplopappin | 127 | momordenol |
| 128 | prostaglandin e1 | 128 | 5-[6-(3-hydroxy-4-methoxyphenyl)-1,3,3a,4,6,6a-hexahydrofuro[3,4-c]furan-3-yl]-2-methoxyphenol |
| 129 | 2-[[7-hydroxy-1-(4-hydroxy-3,5-dimethoxyphenyl)-3-(hydroxymethyl)-6,8-dimethoxy-1,2,3,4-tetrahydronaphthalen-2-yl]methoxy]-6-(hydroxymethyl)oxane-3,4,5-triol | 129 | combrestatin a4 |
| 130 | dihydroisomilletenone methyl ether | 130 | propylparaben |
| 131 | dihydropashanone | 131 | brosimacutin c |
| 132 | (2e)-3,7-dimethyl-2,6-octadienyl acetate | 132 | 2'-hydroxy-3',4',6'-trimethoxydihydrochalcone |
| 133 | 3,4,5-trimethoxycinnamic aldehyde | 133 | 3',4'-dihydroxy-7-methoxy-8-(3-methylbut-2-enyl)-2'''-(1-hydroxy-1-methylethyl)-furano-(4'',5'':6,5)favanone |
| 134 | sphinganine | 134 | 5'-hydroxy-3'-methoxysativan |
| 135 | 1-(3,4-dimethoxyphenyl)-1,2-ethanediol 2-o-b-d-glucoside | 135 | 3-(3,4,5-trimethoxyphenyl)propanoic acid |
| 136 | spinoflavanone a | 136 | undecanoate |
| 137 | dioctyl hexanedioate | 137 | (2s)-4-hydroxy-2-(2-hydroxypropan-2-yl)-7-methyl-2,3-dihydrofuro[3,2-g]chromen-5-one |
| 138 | 1,9b-dihydroxy-6,6,9a-trimethyl-1,5,5a,7,8,9-hexahydrobenzo[e][2]benzofuran-3-one | 138 | benzoylgomisin o |
| 139 | baccatin iii | 139 | 9-hode |
| 140 | osajin | 140 | oleanoic acid |
| 141 | quebrachitol | 141 | p-hydroxyphenethyl trans-ferulate |
| 142 | schizantherin b | 142 | 7-[(2s,3r,4s,5s,6r)-6-[[(2s,3r,4r)-3,4-dihydroxy-4-(hydroxymethyl)oxolan-2-yl]oxymethyl]-3,4,5-trihydroxyoxan-2-yl]oxy-5-hydroxy-3-(4-hydroxyphenyl)-6-methoxychromen-4-one |
| 143 | aurantiamide acetate | 143 | schleicherastatin 6 |
| 144 | n-acetyl-dl-glutamic acid | 144 | resveratrol 4'-methyl ether |
| 145 | (3~{r},5~{s},8~{e})-5,9,14-trimethyl-4,12-dioxatricyclo[9.3.0.0^{3,5}]tetradeca-1(11),8,13-trien-2-one | 145 | 2-methoxyresorcinol |
| 146 | sinapyl alcohol | 146 | 4-methoxy-9,10-dihydrophenanthrene-2,7-diol |
| 147 | 4-hydroxydihydrocinnamaldehyde | 147 | 2',4'-dihydroxydihydrochalcone |
| 148 | cathine | 148 | aesculin |
| 149 | thiamine | 149 | salicifolioside a |
| 150 | 5,5-dimethyl-2(5h)-furanone | 150 | artemisinin |
| 151 | (2s,3r,4s,5r)-2-[(2r,3r,4s,5s,6r)-4,5-dihydroxy-6-(hydroxymethyl)-2-(2-phenylethoxy)oxan-3-yl]oxyoxane-3,4,5-triol | 151 | eudesmic acid |
| 152 | lapachol | 152 | (2s,3r,4r,5r,6s)-2-[(2r,3r,4s,5r,6r)-2-[2-(3,4-dihydroxyphenyl)ethoxy]-3,5-dihydroxy-6-(hydroxymethyl)oxan-4-yl]oxy-6-methyloxane-3,4,5-triol |
| 153 | licarin a | 153 | ferulic acid |
| 154 | 7-hydroxyflavone | 154 | 3,4-di-o-caffeoylquinic acid |
| 155 | 2',4'-dihydroxydihydrochalcone | 155 | garciduol b |
| 156 | cearoin | 156 | shanzhiside methyl ester |
| 157 | 2-aminobutyric acid; lc-tdda; ce20 | 157 | ethyl vanillin |
| 158 | anhydroicaritin | 158 | 3',4'-dihydroxy-5,7-dimethoxyflavan |
| 159 | 12:4+3o fatty acyl hexoside | 159 | evodol |
| 160 | (2r,3s,4s,5r,6s)-2-(hydroxymethyl)-6-(3,4,5-trimethoxyphenoxy)oxane-3,4,5-triol | 160 | [3,4-dihydroxy-4-(7-methoxy-2-oxochromen-8-yl)-2-methylidenebutyl] 3-methylbutanoate |
| 161 | gomisin a | 161 | cearoin |
| 162 | coniferylaldehyde | 162 | 3,4-dihydroxybenzaldehyde |
| 163 | 4-hydroxyphenylacetaldehyde | 163 | benzoic acid |
| 164 | kaempferol 3-p-coumarate | 164 | dulxanthone d |
| 165 | physalin b | 165 | 1,5,8-trihydroxy-3-methyl-2-prenylxanthone |
| 166 | 6,7-dimethoxy-2,2-dimethyl-2h-1-benzopyran | 166 | 5,4'-dihydroxy-6,7-dimethoxyflavanone |
| 167 | methylnissolin-3-o-glucoside | 167 | (2~{r},3~{s},4~{s},5~{r},6~{r})-2-[[(2~{r},3~{r},4~{r})-3,4-dihydroxy-4-(hydroxymethyl)oxolan-2-yl]oxymethyl]-6-phenylmethoxyoxane-3,4,5-triol |
| 168 | anofinic acid | 168 | 2-methoxy-4-hydroxydihydrochalcone |
| 169 | (2~{s},3~{r},4~{s},5~{s},6~{r})-2-[4-[(3~{s},3~{a}~{r},6~{s},6~{a}~{r})-3-(4-hydroxy-3,5-dimethoxyphenyl)-1,3,3~{a},4,6,6~{a}-hexahydrofuro[3,4-c]furan-6-yl]-2,6-dimethoxyphenoxy]-6-(hydroxymethyl)oxane-3,4,5-triol | 169 | isosclerone |
| 170 | episyringaresinol 4'-o-beta-d-glncopyranoside | 170 | 1-acetoxy-4,6-tetradecadiene-8,10,12-triyne |
| 171 | camellianin a | 171 | demethylvestitol |
| 172 | shanzhiside methyl ester | 172 | artonol c |
| 173 | secoisolariciresinol | 173 | sinapic acid; plasma id-325 |
| 174 | rosmadial | 174 | 6-methoxy-[2'',3'':7,8]furanoflavanone |
| 175 | brosimone g | 175 | 5-o-methyllicoricidin |
| 176 | farnesoic acid | 176 | loganic acid |
| 177 | 3-aminoisobutyric acid | 177 | fa 18:2+1o |
| 178 | sinapaldehyde | 178 | beta-glucose pentaacetic acid |
| 179 | phenylacetic acid | 179 | [(1as,1bs,2s,5ar,6s,6as)-1a-(hydroxymethyl)-2-[(2s,3r,4s,5s,6r)-3,4,5-trihydroxy-6-(hydroxymethyl)oxan-2-yl]oxy-2,5a,6,6a-tetrahydro-1bh-oxireno[5,6]cyclopenta[1,3-c]pyran-6-yl] benzoate |
| 180 | 2,4,6-trimethoxyphenyl acetate | 180 | calomelanol h |
| 181 | myristicin | 181 | [3,4,5-trihydroxy-6-(hydroxymethyl)oxan-2-yl] 9-(hydroxymethyl)-2,2,6a,6b,9,12a-hexamethyl-10-[3,4,5-trihydroxy-6-[(3,4,5-trihydroxy-6-methyloxan-2-yl)oxymethyl]oxan-2-yl]oxy-1,3,4,5,6,6a,7,8,8a,10,11,12,13,14b-tetradecahydropicene-4a-carboxylate |
| 182 | (s)-beta-aminoisobutyric acid | 182 | butyl paraben |
| 183 | [(3ar,4s,6e,10z,11ar)-10-(hydroxymethyl)-6-methyl-3-methylidene-2-oxo-3a,4,5,8,9,11a-hexahydrocyclodeca[b]furan-4-yl] (z)-4-acetyloxy-2-(hydroxymethyl)but-2-enoate | 183 | duartin (-) |
| 184 | verimol a | 184 | confertifoline |
| 185 | myrigalone h | 185 | onopordopicrin |
| 186 | 5,7-dioxa-12-azapentacyclo[10.6.1.0^{2,10}.0^{4,8}.0^{13,18}]nonadeca-2,4(8),9,17-tetraene-15,16-diol | 186 | (~{e})-1-(1,3,6,8-tetramethoxynaphthalen-2-yl)but-2-en-1-one |
| 187 | 3-(2-hydroxy-4-methoxyphenyl)-3,4-dihydro-2h-1-benzopyran-7-ol | 187 | chlorogenate |
| 188 | gambiriin b3 | 188 | 4-methoxybenzyl acetate |
| 189 | 8-desoxygartanin | 189 | fraxin |
| 190 | paeonilactone b | 190 | otobanone |
| 191 | [(2s,3r,4s,5s,6r)-3,4,5-trihydroxy-6-(hydroxymethyl)oxan-2-yl] (2e,6e)-8-hydroxy-2,6-dimethylocta-2,6-dienoate | 191 | 5,7-dihydroxy-2-methyl-8-[(2~{s},3~{r},4~{r},5~{s},6~{r})-3,4,5-trihydroxy-6-(hydroxymethyl)oxan-2-yl]chromen-4-one |
| 192 | aspalathin | 192 | 7-methoxy-6-(1,2,3-trihydroxy-3-methylbutyl)chromen-2-one |
| 193 | glabranin | 193 | (2s)-4'-hydroxy-5,7,3'-trimethoxyflavan |
| 194 | trans-pterostilbene | 194 | ricinine |
| 195 | citrinin | 195 | epicalyxin j |
| 196 | curcumin | 196 | rescinnamine |
| 197 | tephrosin | 197 | tephrorianin |
| 198 | pinocembrin 7-o-benzoate | 198 | bungeiside c |
| 199 | isosafrole | 199 | methyl (1s,4as,7as)-4'-[(1s)-1-[(e)-3-(4-hydroxyphenyl)prop-2-enoyl]oxyethyl]-5'-oxo-1-[(2s,3r,4s,5s,6r)-3,4,5-trihydroxy-6-(hydroxymethyl)oxan-2-yl]oxyspiro[4a,7a-dihydro-1h-cyclopenta[c]pyran-7,2'-furan]-4-carboxylate |
| 200 | oleuropein aglycon derivative | 200 | paclitaxel |
| 201 | kushenol c | 201 | riboflavin |
| 202 | 7,8,3',4',5'-pentamethoxy-6'',6''-dimethylpyrano[2'',3'':5,6]flavone | 202 | flavone base + 2o, 2meo, c-hex |
| 203 | yangambin | 203 | 7-methoxyflavonol |
| 204 | calycosin-7-o-beta-d-glucoside | 204 | pratol |
| 205 | (1e,4z,6e)-5-hydroxy-1,7-bis(4-hydroxy-3-methoxyphenyl)hepta-1,4,6-trien-3-one | 205 | 7-hydroxy-5,4'-dimethoxyflavan |
| 206 | 5,7-dihydroxyflavanone | 206 | lycorine |
| 207 | chrysin | 207 | (3r)-3-(3,4-dimethoxyphenyl)-8-hydroxy-3,4-dihydroisochromen-1-one |
| 208 | moghanin a | 208 | 3,4-dimethyl-2,5-bis(3,4,5-trimethoxyphenyl)oxolane |
| 209 | 7,4'-dihydroxyflavone | 209 | haplopappin |
| 210 | piscidic acid | 210 | 3-(2-hydroxy-3,4-dimethoxyphenyl)-3,4-dihydro-2~{h}-chromen-7-ol |
| 211 | 2,4,4'-trihydroxydihydrochalcone | 211 | 3-phenyllactic acid |
| 212 | (1s,4as,7s,7as)-7-hydroxy-7-methyl-1-[(2s,3r,4s,5s,6r)-3,4,5-trihydroxy-6-(hydroxymethyl)oxan-2-yl]oxy-4a,5,6,7a-tetrahydro-1h-cyclopenta[c]pyran-4-carboxylic acid | 212 | lariciresinol |
| 213 | s(8-8)s hexoside | 213 | 3,7-dimethyluric acid |
| 214 | 10-methylacridone | 214 | 4-hydroxy-3-tetratrenylbenzoic acid |
|  |  | 215 | 2'-hydroxydaidzein |
|  |  | 216 | luteolinidin |
|  |  | 217 | ethyl 3-(methylthio)propanoate |
|  |  | 218 | (-)-mucronulatol |
|  |  | 219 | 3,5-dimethoxyphenol |
|  |  | 220 | 4-acetamidobutanoate |
|  |  | 221 | dehydrorotenone |
|  |  | 222 | (e)-3-(2-hydroxyphenyl)-2-propenal |
|  |  | 223 | 3-hydroxyphenylacetic acid |
|  |  | 224 | (-)-gallocatechin |
|  |  | 225 | trans-cinnamic acid |
|  |  | 226 | tangeritin |
|  |  | 227 | brosimacutin i |
|  |  | 228 | (9a-hydroxy-3,8a-dimethyl-5-methylidene-2-oxo-4,4a,6,7,8,9-hexahydrobenzo[f][1]benzofuran-8-yl) acetate |
|  |  | 229 | 2-methoxyhomopterocarpin |
|  |  | 230 | 5,7,8-trimethoxyflavanone |
|  |  | 231 | calycosin |
|  |  | 232 | 5,7-dihydroxy-2'-methoxyflavone |
|  |  | 233 | glycitin |
|  |  | 234 | methylophiopogonanone b |
|  |  | 235 | nicotiflorin |
|  |  | 236 | lethedoside b |
|  |  | 237 | grossamide or its isomer (not validated) |
|  |  | 238 | [8-[2-(3-methylbutanoyloxy)propan-2-yl]-2-oxo-8,9-dihydrofuro[2,3-h]chromen-9-yl] 3-methylbutanoate |
|  |  | 239 | cratoxyarborenone e |
|  |  | 240 | 12:4+3o fatty acyl hexoside |
|  |  | 241 | ougenin |
|  |  | 242 | 3,5-dihydroxybenzoic acid |
|  |  | 243 | methyl (4~{s},5~{e},6~{s})-5-ethylidene-4-[2-oxo-2-[(2~{s},3~{r},4~{s},5~{s},6~{r})-3,4,5-trihydroxy-6-(hydroxymethyl)oxan-2-yl]oxyethyl]-6-[(2~{s},3~{r},4~{s},5~{s},6~{r})-3,4,5-trihydroxy-6-(hydroxymethyl)oxan-2-yl]oxy-4~{h}-pyran-3-carboxylate |
|  |  | 244 | osajin |
|  |  | 245 | herniarin |
|  |  | 246 | 6,7,3',4'-tetrahydroxyflavanone |
|  |  | 247 | curcumin |
|  |  | 248 | (2z)-3-(4-methoxy-2-{[(2s,3r,4s,5s,6r)-3,4,5-trihydroxy-6-(hydroxymethyl)oxan-2-yl]oxy}phenyl)prop-2-enoic acid |
|  |  | 249 | homopisatin |
|  |  | 250 | 2',4',6'-trihydroxy-4-methoxydihydrochalcone |
|  |  | 251 | (2~{r},3~{r},4~{s},5~{s},6~{r})-2-[[(2~{r},3~{r},4~{s})-6-hydroxy-4-(4-hydroxy-3-methoxyphenyl)-3-(hydroxymethyl)-7-methoxy-1,2,3,4-tetrahydronaphthalen-2-yl]methoxy]-6-(hydroxymethyl)oxane-3,4,5-triol |
|  |  | 252 | 1,5-dihydroxy-2,3-dimethoxy-10-methylacridin-9-one |
|  |  | 253 | physalin e |
|  |  | 254 | aspartate |
|  |  | 255 | epishyobunone |
|  |  | 256 | 2-(3-hydroxy-5-methoxyphenoxy)-6-(hydroxymethyl)oxane-3,4,5-triol |
|  |  | 257 | myrigalone h |
|  |  | 258 | 5,7-dioxa-12-azapentacyclo[10.6.1.0^{2,10}.0^{4,8}.0^{13,18}]nonadeca-2,4(8),9,17-tetraene-15,16-diol |
|  |  | 259 | karanjin |
|  |  | 260 | oroxylin a |
|  |  | 261 | apocynin |
|  |  | 262 | 5-hydroxymethyl-2-furancarboxylic acid |
|  |  | 263 | indole |
|  |  | 264 | 3,9-dimethoxy-6~{a},11~{a}-dihydro-6~{h}-[1]benzofuro[3,2-c]chromene |
|  |  | 265 | leonoside b |
|  |  | 266 | isoamericanin a |
|  |  | 267 | pinosylvin |
|  |  | 268 | ligustilide |
|  |  | 269 | lapachol |
|  |  | 270 | fragransol b |
|  |  | 271 | salviaflaside |
|  |  | 272 | farrerol |
|  |  | 273 | 5,7-dihydroxy-2-(4-hydroxyphenyl)-8-[3,4,5-trihydroxy-6-(hydroxymethyl)oxan-2-yl]-6-(3,4,5-trihydroxyoxan-2-yl)chromen-4-one |
|  |  | 274 | trans-pterostilbene |
|  |  | 275 | austrobailignan 7 |
|  |  | 276 | aschantin |
|  |  | 277 | 7-hydroxy-2-(4-hydroxy-3,5-dimethoxyphenyl)-5-[(2s,3r,4s,5s,6r)-3,4,5-trihydroxy-6-(hydroxymethyl)oxan-2-yl]oxychromen-4-one |
|  |  | 278 | tomatine |
|  |  | 279 | citrinin |
|  |  | 280 | 2'-hydroxy-4',6'-dimethoxy-3'-methylacetophenone |
|  |  | 281 | cis-mulberroside a |
|  |  | 282 | multigilin |
|  |  | 283 | 3,4,5-trihydroxystilbene; plasma id-333 |
|  |  | 284 | trans-resveratrol |
|  |  | 285 | isotaxiresinol |
|  |  | 286 | 7,4'-dihydroxyflavone |
|  |  | 287 | piscidic acid |
|  |  | 288 | 3-methoxybenzaldehyde |
|  |  | 289 | picroside ii |
|  |  | 290 | rehmannioside a |
|  |  | 291 | (+)-8-hydroxy-5,5-dimethylpeltogynan |
|  |  | 292 | sempervirenoside b |
|  |  | 293 | methyl 1,4~{a}-dimethyl-6-methylidene-5-[2-(5-oxo-2~{h}-furan-4-yl)ethyl]-3,4,5,7,8,8~{a}-hexahydro-2~{h}-naphthalene-1-carboxylate |
|  |  | 294 | 1-methoxyindole-3-carbaldehyde |
|  |  | 295 | tocotrienol |
|  |  | 296 | isomaltose |
|  |  | 297 | columbianetin |
|  |  | 298 | 5,7-dimethoxy-3-(4-methoxyphenyl)-4h-chromen-4-one |
|  |  | 299 | angoroside a |
|  |  | 300 | trans-12-hydroxyjasmonic acid |
|  |  | 301 | 3-(3,4-dihydroxyphenyl)-5,7-dihydroxy-6,8-bis(3-methylbut-2-enyl)chromen-4-one |
|  |  | 302 | beta-sinensal |
|  |  | 303 | adenosine |
|  |  | 304 | benzoic acid + 1o, 1meo, o-hex |
|  |  | 305 | 2-phenylethanol |
|  |  | 306 | isobrucein a |
|  |  | 307 | 5'-prenylhomoeriodictyol |
|  |  | 308 | stictic acid |
|  |  | 309 | palmitic acid |
|  |  | 310 | alpha-tocopherol |
|  |  | 311 | n~1~,n~4~-bis(3-aminopropyl)-1,4-butanediamine |
|  |  | 312 | 9-hydroxy-10,12-octadecadienoic acid |
|  |  | 313 | betulafolienetriol |
|  |  | 314 | 4-methylvaleric acid |
|  |  | 315 | schisandrin c |
|  |  | 316 | ergostane-3,6-dione |
|  |  | 317 | tomatidine |
|  |  | 318 | (4~{a}~{s},6~{a}~{s},6~{b}~{r},10~{s},12~{a}~{r})-10-hydroxy-2,2,6~{a},6~{b},9,9,12~{a}-heptamethyl-1,3,4,5,6,6~{a},7,8,8~{a},10,11,12,13,14~{b}-tetradecahydropicene-4~{a}-carboxylic acid |
|  |  | 319 | linoleic acid |
|  |  | 320 | 12(r)-hepe |
|  |  | 321 | epimedoside c |
|  |  | 322 | 3,7-dimethoxyflavone |
|  |  | 323 | 5,7-dimethoxy-2-phenyl-4h-chromen-4-one |
|  |  | 324 | crotonoside |
|  |  | 325 | phloridzin |
|  |  | 326 | tanshinone iia |
|  |  | 327 | artoindonesianin a |
|  |  | 328 | robinin |
|  |  | 329 | (2~{s},3~{r},5~{r},10~{r},13~{r},14~{s},17~{s})-2,3,14-trihydroxy-10,13-dimethyl-17-[(2~{r},3~{r})-2,3,6-trihydroxy-6-methylheptan-2-yl]-2,3,4,5,9,11,12,15,16,17-decahydro-1~{h}-cyclopenta[a]phenanthren-6-one |
|  |  | 330 | 17-[2,3-dihydroxy-6-methyl-6-[3,4,5-trihydroxy-6-(hydroxymethyl)oxan-2-yl]oxyheptan-2-yl]-2,3,14-trihydroxy-10,13-dimethyl-2,3,4,5,9,11,12,15,16,17-decahydro-1~{h}-cyclopenta[a]phenanthren-6-one |
|  |  | 331 | 5,7-dihydroxy-2-phenyl-6-[3,4,5-trihydroxy-6-(hydroxymethyl)oxan-2-yl]-8-(3,4,5-trihydroxyoxan-2-yl)chromen-4-one |
|  |  | 332 | anthothecol |
|  |  | 333 | didymin |
|  |  | 334 | 2-[[3,4-dihydroxy-4-(hydroxymethyl)oxolan-2-yl]oxymethyl]-6-[(5-hydroxy-1,7,7-trimethyl-2-bicyclo[2.2.1]heptanyl)oxy]oxane-3,4,5-triol |
|  |  | 335 | (2r,3r,4s,5s,6r)-2-[(3r)-1,7-bis(3,4-dihydroxyphenyl)heptan-3-yl]oxy-6-(hydroxymethyl)oxane-3,4,5-triol |
|  |  | 336 | 7,8,3',4',5'-pentamethoxy-6'',6''-dimethylpyrano[2'',3'':5,6]flavone |
|  |  | 337 | histidine |
|  |  | 338 | catalposide |
|  |  | 339 | 3-[(2s,3r,4s,5r,6r)-3,5-dihydroxy-6-(hydroxymethyl)-4-[(2s,3r,4r,5r,6s)-3,4,5-trihydroxy-6-methyloxan-2-yl]oxyoxan-2-yl]oxy-5,7-dihydroxy-2-(4-hydroxyphenyl)chromen-4-one |
|  |  | 340 | oxalic acid |
|  |  | 341 | [2-[(2~{s},3~{r},4~{s},5~{s},6~{r})-3,4,5-trihydroxy-6-(hydroxymethyl)oxan-2-yl]oxyphenyl]methyl 2-hydroxy-6-[(2~{s},3~{r},4~{s},5~{s},6~{r})-3,4,5-trihydroxy-6-(hydroxymethyl)oxan-2-yl]oxybenzoate |
|  |  | 342 | yangonin |
|  |  | 343 | atractylenolide iii |
|  |  | 344 | gibberellic acid |
|  |  | 345 | (+)-dihydrowighteone |
|  |  | 346 | angoletin |
|  |  | 347 | tetrahydropalmatine |
|  |  | 348 | stevioside |
|  |  | 349 | homoferreirin |
|  |  | 350 | 2'-methoxyflavone |
|  |  | 351 | feruloyltyramine |
|  |  | 352 | daphylloside |
|  |  | 353 | angelol b |
|  |  | 354 | typhaneoside |
|  |  | 355 | samaderin a |
|  |  | 356 | cardamonin |
|  |  | 357 | flavokawain a |
|  |  | 358 | myristicin |
|  |  | 359 | bryacarpene 5 |
|  |  | 360 | 6,11-dihydroxy-2,2-dimethylpyrano[3,2-c]xanthen-7(2h)-one |
|  |  | 361 | xanthyletin |
|  |  | 362 | methylnissolin-3-o-glucoside |
|  |  | 363 | fisetinidol |
|  |  | 364 | gossypol |
|  |  | 365 | epiafzelechin (2r,3r)(-) |
|  |  | 366 | 2-aminobutyric acid; lc-tdda; ce20 |
